# Natural selection and recombination at host-interacting lipoprotein loci drive genome diversification of Lyme disease and related bacteria

**DOI:** 10.1128/mbio.01749-24

**Published:** 2024-08-15

**Authors:** Saymon Akther, Emmanuel F. Mongodin, Richard D. Morgan, Lia Di, Xiaohua Yang, Maryna Golovchenko, Natalie Rudenko, Gabriele Margos, Sabrina Hepner, Volker Fingerle, Hiroki Kawabata, Ana Cláudia Norte, Isabel Lopes de Carvalho, Maria Sofia Núncio, Adriana Marques, Steven E. Schutzer, Claire M. Fraser, Benjamin J. Luft, Sherwood R. Casjens, Weigang Qiu

**Affiliations:** 1 Graduate Center and Hunter College, City University of New York, New York, New York, USA; 2 University of Maryland School of Medicine, Baltimore, Maryland, USA; 3 New England BioLabs, Ipswich, Massachusetts, USA; 4 Department of Medicine, Renaissance School of Medicine, Stony Brook University (SUNY), Stony Brook, New York, USA; 5 Biology Centre Czech Academy of Sciences, Institute of Parasitology, České Budějovice, Czech Republic; 6 Bavarian Health and Food Safety Authority and German National Reference Centre for Borrelia, Oberschleissheim, Bavaria, Germany; 7 National Institute of Infectious Diseases, Tokyo, Japan; 8 Department of Life Sciences, University of Coimbra, MARE-Marine and Environmental Sciences Centre, Coimbra, Portugal; 9 Centre for Vector and Infectious Diseases Research, Águas de Moura, Portugal; 10 National Institute of Allergy and Infectious Diseases, Bethesda, Maryland, USA; 11 New Jersey Medical School, Newark, New Jersey, USA; 12 University of Utah School of Medicine and School of Biological Sciences, Salt Lake City, Utah, USA; 13 Weill Cornell Medical College, New York, New York, USA; University of Pittsburgh School of Medicine, Pittsburgh, Pennsylvania, USA

**Keywords:** *Borrelia burgdorferi*, recombination, Lyme disease, evolution, genome diversification, plasmids

## Abstract

Lyme disease, caused by spirochetes in the *Borrelia burgdorferi sensu lato* clade within the *Borrelia* genus, is transmitted by *Ixodes* ticks and is currently the most prevalent and rapidly expanding tick-borne disease in Europe and North America. We report complete genome sequences of 47 isolates that encompass all established species in this clade while highlighting the diversity of the widespread human pathogenic species *B. burgdorferi*. A similar set of plasmids has been maintained throughout *Borrelia* divergence, indicating that they are a key adaptive feature of this genus. Phylogenetic reconstruction of all sequenced *Borrelia* genomes revealed the original divergence of Eurasian and North American lineages and subsequent dispersals that introduced *B. garinii, B. bavariensis, B. lusitaniae, B. valaisiana,* and *B. afzelii* from East Asia to Europe and *B. burgdorferi* and *B. finlandensis* from North America to Europe. Molecular phylogenies of the universally present core replicons (chromosome and cp26 and lp54 plasmids) are highly consistent, revealing a strong clonal structure. Nonetheless, numerous inconsistencies between the genome and gene phylogenies indicate species dispersal, genetic exchanges, and rapid sequence evolution at plasmid-borne loci, including key host-interacting lipoprotein genes. While localized recombination occurs uniformly on the main chromosome at a rate comparable to mutation, lipoprotein-encoding loci are recombination hotspots on the plasmids, suggesting adaptive maintenance of recombinant alleles at loci directly interacting with the host. We conclude that within- and between-species recombination facilitates adaptive sequence evolution of host-interacting lipoprotein loci and contributes to human virulence despite a genome-wide clonal structure of its natural populations.

**IMPORTANCE:**

Lyme disease (also called Lyme borreliosis in Europe), a condition caused by spirochete bacteria of the genus *Borrelia*, transmitted by hard-bodied *Ixodes* ticks, is currently the most prevalent and rapidly expanding tick-borne disease in the United States and Europe. *Borrelia* interspecies and intraspecies genome comparisons of Lyme disease-related bacteria are essential to reconstruct their evolutionary origins, track epidemiological spread, identify molecular mechanisms of human pathogenicity, and design molecular and ecological approaches to disease prevention, diagnosis, and treatment. These Lyme disease-associated bacteria harbor complex genomes that encode many genes that do not have homologs in other organisms and are distributed across multiple linear and circular plasmids. The functional significance of most of the plasmid-borne genes and the multipartite genome organization itself remains unknown. Here we sequenced, assembled, and analyzed whole genomes of 47 *Borrelia* isolates from around the world, including multiple isolates of the human pathogenic species. Our analysis elucidates the evolutionary origins, historical migration, and sources of genomic variability of these clinically important pathogens. We have developed web-based software tools (BorreliaBase.org) to facilitate dissemination and continued comparative analysis of *Borrelia* genomes to identify determinants of human pathogenicity.

## INTRODUCTION

Microbial species tend to be widely distributed wherever habitats allow, hence the Bass-Becking hypothesis of microbial biogeography that “everything is everywhere—the environment selects” (1). A lack of biogeographic structures, promiscuous genetic exchanges, and strong natural selection conspire to hinder reliable reconstruction of the history and mechanisms of microbial evolution in nature (2). For example, bacterial genomes are compact and lack selectively neutral polymorphisms necessary for accurately estimating population sizes, testing natural selection, and reconstructing timed phylogenies (3). Furthermore, frequent dispersals, a lack of species barriers to gene flow, genome-wide linkage disequilibrium, and periodic selective sweeps quickly erase or confound genetic signatures of bacterial population history (2, 4–6). Geographically structured bacterial populations—due to a parasitic association with a host—like the Lyme Disease agent and related bacteria studied here offer rare opportunities for reconstructing the history and mechanisms of bacterial evolution in nature.

Lyme disease, termed Lyme borreliosis in Europe, is the most common tick-borne illness in the United States and Europe (7–12). The etiological agents of Lyme disease are spirochetes belonging to the genus *Borrelia*. These bacteria, which are transmitted by the bite of infected ticks of the *Ixodes ricinus* complex, are also known collectively as the *Borrelia burgdorferi sensu lato* (Bbsl, the term we use here) species group or the Lyme agent borrelias (13, 14). While the genus *Borrelia* was tentatively split into relapsing fever (*Borrelia*) and Lyme borreliosis-related (*Borreliella*) genera, it has been argued that the split was not adequately scientifically supported (15–19). However, NCBI GenBank uses the *Borreliella* nomenclature to refer to this group. The Bbsl group currently contains 23 established species and two recently proposed South American species (14, 20, 21). While four species in this group—*B. burgdorferi*, *B. afzelii*, *B. bavariensis,* and *B. garinii—*cause most cases of human Lyme disease, *B. bissettiae*, *B. lusitaniae*, *B. mayonii,* and *B. spielmanii* have also been reported to infect humans (22–27).

The Bbsl species maintain an enzootic transmission cycle between *Ixodes* tick vectors and vertebrate reservoir hosts (28–31). Humans are incidental hosts and do not contribute to the transmission or spread of disease. It has been hypothesized that ongoing climate change and declining biodiversity have contributed to the observed expansion of the geographic range of Lyme disease-associated pathogens as well as their tick and vertebrate host populations across North America and Europe (30, 32–35). The continued rise of Lyme and other tick-borne infectious diseases is an emerging public health issue (30, 36).

Critical knowledge gaps remain in our understanding of Bbsl species divergence and range expansion. The evolutionary history of this genus has not been fully explored due to under-sampling and the lack of high-quality reference genome sequences of a majority of the formally described species. Although the genome of *B. burgdorferi* strain B31 was among the first bacterial genomes to be sequenced, genomes of members of this genus remain among the most challenging to fully sequence and assemble because of their numerous circular and linear plasmids (up to 23 distinct plasmids in a single cell) that harbor numerous sequence repeats (37–46). Their genomes include three universally present replicons: the ~900 kbp linear main chromosome, the linear plasmid lp54 (typically 53–60 kbp), and the circular plasmid cp26 (typically 26–27 kbp) (42, 44, 47–49).

Bbsl phylogeny based on chromosomal multi-locus sequence typing (MLST) (47, 50–54) and on the analysis of single-nucleotide variants (SNVs; also called single-nucleotide polymorphisms or SNPs) has previously revealed the presence of three major clades, one that includes species found mainly in Eurasia, one with species found mainly in North America, and a third represented by the single isolate from South America (44, 47, 55, 56). Furthermore, Bbsl species exhibit diversity within natural populations; for example, there are currently about 20 genomically and serologically distinct subtypes of *B. burgdorferi,* most of which co-exist in the northeastern United States (52, 57–60). The lack of genome sequence information from a majority of the species in the genus has severely limited the study of its phylogeny (14).

To remedy this dearth of information, we sequenced the complete genomes of 47 Bbsl isolates chosen to maximize geographic, phylogenetic, and antigenic diversity. In this report, we use this new information to perform a comprehensive and detailed phylogeographic analysis of the Bbsl branch of the *Borrelia* genus. Our results bring new understandings of the evolutionary histories and ecological mechanisms associated with intercontinental dispersal, intra-species recombination, cross-species introgression, and natural selection targeting host-interacting genes.

## RESULTS

### Genome sequences and structural diversity

#### Species and isolates

**Fig 1:**
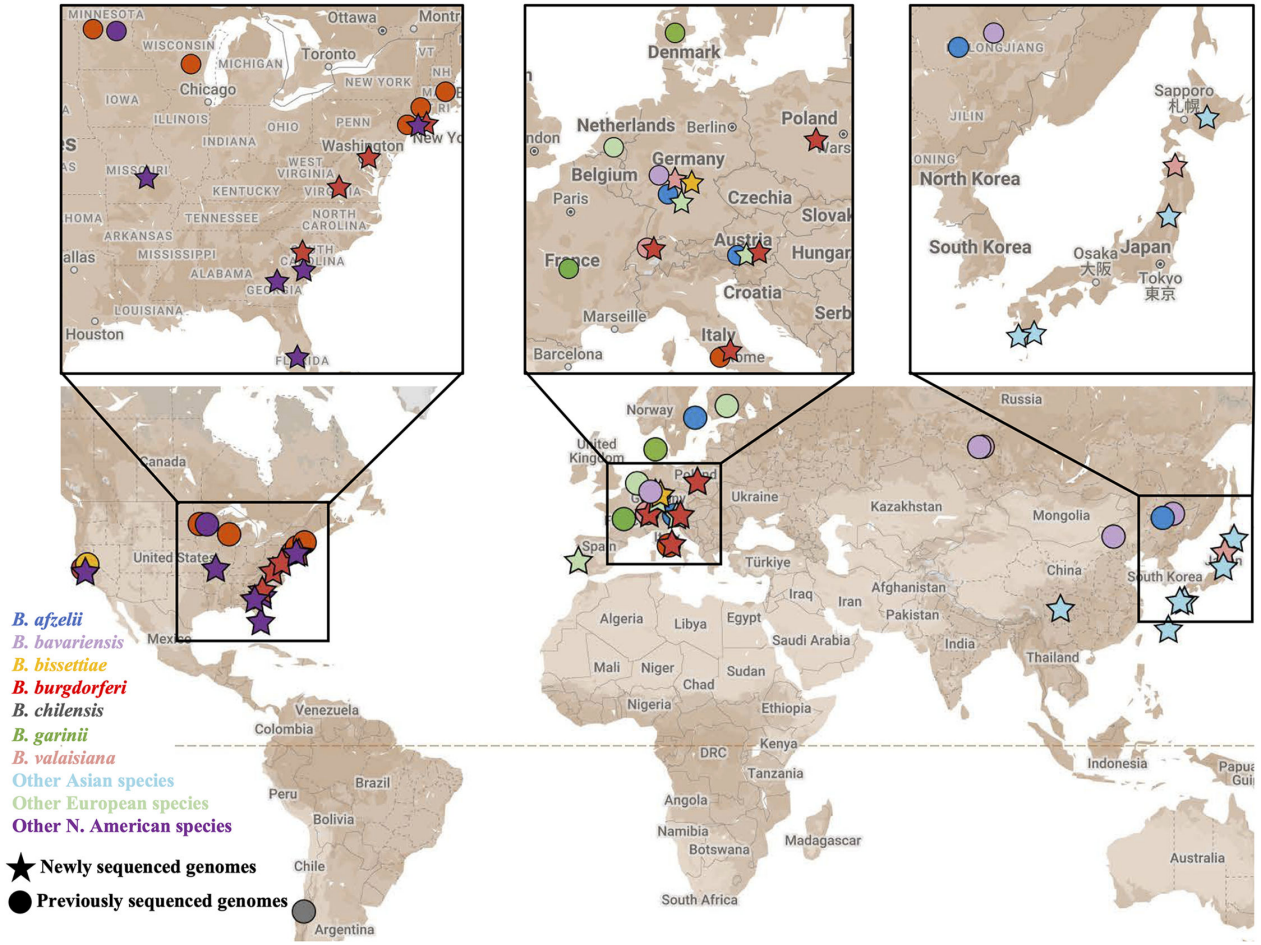
Geographic origins of *Bbsl* isolates with complete genome sequences. Each point on the map represents a sequenced *Borrelia* genome and is colored according to major species groups (see key). Stars and circles indicate newly (*n* = 47) or previously (*n* = 31) sequenced genomes (Tables S1A and S1B, respectively). Multiple genomes of the same species from the same geographic location are shown only once. Map data ©2024 Google, INEGI.

At the start of this project, full-genome sequences were available for only 10 of the 23 Bbsl species, *B. afzelii, B. bavariensis, B. bissettiae, B. burgdorferi, B. chilensis, B. finlandensis, B. garinii, B. mayonii, B. spielmanii,* and *B. valaisiana* (37–39, 41, 42, 61–63). We therefore determined whole-genome sequences of 47 Bbsl isolates that include the first whole-genome sequences for the following 13 species: *B. americana, B. andersonii, B. californiensis, B. carolinensis, B. kurtenbachii, B. lanei, B. maritima, B. japonica, B. lusitaniae, B. sinica*, *B. tanukii*, *B. turdi,* and *B. yangtzensis* [during this work, genome sequences for *B. maritima* and *B. turdi* isolates were independently reported (64, 65)]. We also obtained genome sequences of additional geographically distinct isolates of *B. bissettiae, B. finlandensis, B. spielmanii, B. valaisiana,* and *B. burgdorferi*. The latter includes 13 geographically diverse isolates that encompass 6 OspC major-group allele types (H, K, N, L, S, and a new allele “NE”) (57, 66, 67). Thus, complete genome sequences are now available for all 23 currently established Bbsl species and for 20 major OspC types of *B. burgdorferi*. These 47 additional completely sequenced isolates are listed in Table 1, and Table S1A gives their sequence accession number and isolation information. Table S1B gives information for the 31 other genome sequences used in this study. Figure 1 shows the collection location of these isolates on a world map. The new sequences and comparative information are also available in the updated online Bbsl genome database (https://borreliabase.org). This web-based database facilitates visualization and retrieval of comparative information and includes genome phylogeny, plasmid composition, gene synteny, sequence alignments, and phylogenetic trees of individual gene families (68). The most constant “core” replicons of the genome—the main chromosome, the lp54, and cp26 plasmids—which are universally present, and the plasmid lp17, which is present in all but one (*B. carolinensis* SCW-22) of the available complete genomes—are included in the analyses in this report. Genomic analysis of the variable “non-core” plasmids that comprise the rest of the genome is complicated by their rapid evolution, the presence of numerous pseudogenes, and frequent rearrangements (47–49); their analysis is beyond the scope of this report and will be the subject of subsequent publications.

**TABLE 1:** Genome sequences reported in this study

Species	Isolates^*a*^
*B. americana* ^*b*^	SCW-41(T), SCW-30h
*B. andersonii* ^*b*^	21038(T), MOD-5
*B. bissettiae*	PGeb
*B. burgdorferi*	217_5, 80a, Bol29, Fr-93-1, NE5248, NE5261, NE5267, NIH3, NIH5, NIH8, Sh-2-82, Z9
*B. californiensis* ^*b*^	CA446(T), CA443
*B. carolinensis* ^*b*^	SCW-22(T), SCGT-18
*B. finlandensis*	Z11
*B. japonica* ^*b*^	HO14(T), Miyazaki2E
*B. kurtenbachii* ^*b*^	25015(T)
*B. lanei^b^*	CA-28–91(T)
*B. lusitaniae* ^*b*^	PotiB2(T), PotiB3, PoHL1
*B. maritima* ^*b*^	CA690(T)
*B. sinica* ^*b*^	CMN3(T)
*B. spielmanii*	PMew
*B. tanukii* ^*b*^	Hk501(T), Koshiki4E, TanegashimaAS9, TanegashimaAS13
*B. turdi* ^*b*^	Ya501(T), 047–3
*B. valaisiana*	Am501, 100B40
*B. yangtzensis* ^*b*^	Okinawa-CW62(T)*,* Okinawa-CW61, Miyako4E

^*a*^ See Table S1A for isolate details and accession information; (T), species type strain.

^*b*^ Species whose genome has not been previously sequenced. Since the start of this project *B. maritima* CA690 was sequenced independently by Margos et al. (64) and several *B. turdi* isolates were sequenced by Margos et al. (65). *B. andersonii* remains an unvalidated provisional species at present (69).

#### Chromosome structure

The Bbsl chromosomes all exhibit very similar gene contents and are fully syntenic in all 23 Bbsl species, with only a small number of exceptions. Among the exceptions are some chromosomes that have 1–22 kbp plasmid-like extensions at their ends. Previously sequenced genomes have chromosomal extensions either on the left end (*B. mayonii* and *B. valaisiana*) or the right end (e.g., *B. burgdorferi*, *B. valaisiana*, and *B. turdi*) (44, 45, 49, 65). Among the newly sequenced chromosomes presented here, previously undescribed >1 kbp extensions are present at chromosome left ends in *B. japonica, B. lusitaniae,* and *B. valaisiana*, and at chromosome right ends in *B. americana, B. burgdorferi, B. japonica, B. lanei, B. maritima, B. tanukii, B. turdi, B. valaisiana,* and *B. yangtzensis*. These newly identified extensions are shown in Fig. S1A and B, where the Bbsl protein families (PFam’s; previously “paralogous protein families”) (38, 48) are indicated for the major open reading frames (ORFs) in the extensions. As in previously analyzed extensions, nearly all the gene types in these extensions are plasmid-borne in other isolates. We also note that (i) the two *japonica* isolates, HO14 and Miyazaki2E, have the same chromosomal “constant region” rearrangement near the left end of the chromosome and as a result are missing genes *bb_002* and *bb_003* and have gene *bb_004* inverted relative to the other genomes, and (ii) the European *B. valaisiana* genomes, VS116 and 100B40, constant region chromosomal gene *bb_001* is at the right, rather than the left end of the chromosome, while the Asian *valaisiana* genome does not have this rearrangement (Fig. S1B). A more detailed gene content analysis will be presented in a subsequent publication.

#### Plasmid diversity

The 47 newly sequenced genomes presented here contain a total of 608 plasmids. Of these, 521 were completely sequenced with linear plasmid sequences that extend to the tips of both telomeres and circularized cp9, cp26, and cp32 plasmid sequences. Plasmids in these genomes were named based on their encoded PFam32 partition proteins as is traditional in this field (38, 47, 49). The following four new plasmid PFam32 compatibility types were identified in these genomes: (i) type lp28-12 was identified in *B. turdi* isolates Ya501 and 047–3. Margos et al. (65) independently identified this type in their *B. turdi* genome sequences of isolates T2084, TPT2017, and T1190A where it was called lp30; (ii) lp28-13 was identified in *B. turdi* isolate Ya501; (iii) lp28-14 was identified in *B. andersonii* isolate MOD-5; and (iv) a new cp32 type designated cp32-14 was identified in *B. carolinensis* BUL-H-2. Figure S2 shows a PFam32 protein neighbor-joining tree that demonstrates these new PFam32 categories. We also found the lp28-4 PFam32 gene type, which was previously known only on linear plasmids, present as the only PFam32 gene on several circular, otherwise cp32-like plasmids in the newly sequenced genomes. We name such circular plasmids cp32-28-4, and they are present in isolates of *B. japonica*, *B. lusitaniae*, *B. sinica, B. tanukii*, *B. valaisiana,* and *B. yangtzensis*.

**Fig 2:**
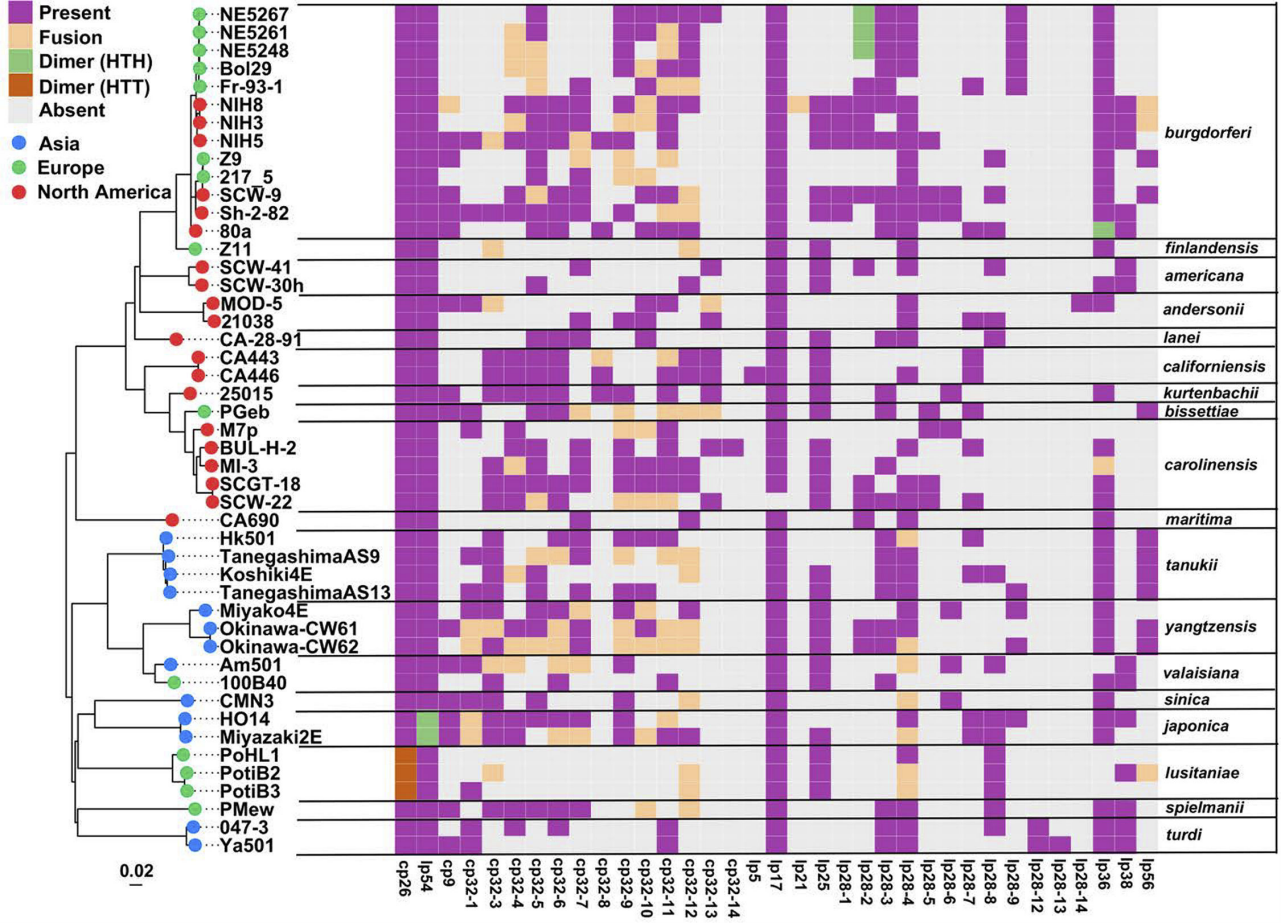
Plasmids in the newly sequenced genomes. The heatmap depicts the presence and absence of 35 plasmid types (columns) classified according to the encoded PFam32 protein type (49) in the 47 newly sequenced *Borreliella* genomes (rows). The continent of isolation and plasmid status is indicated in the key (HTH, head-to-head; HTT, head-to-tail). Genomes are arranged according to the chromosomal phylogeny determined in this report (tree on the *left*; see Fig. 4) with branch tips colored according to geographic origins. Previously identified plasmid lp28-11 is not shown since it is not present in any of these isolates. Linear and circular plasmids of the same compatibility group are not distinguished in the figure; so circular plasmid cp32-28-4 (see text) is listed under lp28-4, and linear lp32 plasmids are listed under their cognate cp32 plasmid. We note that some cp9 and all lp5 *Borreliella* plasmids encode no PFam32 protein. Lp5-like plasmids carry only a PFam57 partitioning gene (38); the “lp5” in strain CA446 encodes a cp32-like PFam57 protein that may impart different compatibility from lp5 itself (see text).

Figure 2 and Table S2 show the 35 plasmid types present in the 47 genomes reported here. The total plasmid numbers in these isolates range from 6 to 20. Plasmids are most abundant in the *B. burgdorferi* genomes with a range of 11 to 20 plasmids, and they are least abundant in the *B. finlandensis, B. maritima,* and *B. lusitaniae* genomes (7, 8, and 6 to 8 plasmids, respectively); however, it is unclear if isolate numbers are large enough to consider these relative plasmid abundances to be universal species properties. The core plasmids cp26 and lp54 are present in all these genomes and are largely syntenic; the few exceptional syntenies are discussed below in the “Plasmid dynamics” section. Although it is present in all other completely sequenced Bbsl genomes, lp17 is absent from the *B. carolinensis* SCW-22 genome sequence reported here (it is not known if this lp17 could have been lost in post-isolation culture in the laboratory). We do not consider lp17 to be a true “core” plasmid since its left half is widely variable (45, 49) (see below), but it is nonetheless included in the analyses of this report. The remaining 468 non-core, “variable” plasmids are present sporadically without obvious phylogenetic consistency in the genomes presented here; they are not considered in detail in this analysis and will be analyzed in subsequent publications.

Some cp9’s and all lp5 Bbsl plasmids do not carry a PFam32 gene and so cannot be categorized by their PFam32 proteins; however, in all cases, they do encode a PFam57 partition protein. It has been suggested that the universally plasmid-encoded PFam57 proteins might be involved in the compatibility and/or replication of these plasmids (45), but this remains somewhat speculative. Thus, in Fig. 2, all circular cp9s are placed in the same column regardless of whether they encode a PFam32 protein. Previously, the only known lp5’s were closely related, very short ~5 kbp linear plasmids in *B. burgdorferi* and *B. mayonii* isolates. Among the newly sequenced genomes presented here, only *B. californiensis* CA446 carries a linear plasmid with no PFam32 gene; however, at 17 kbp, it is substantially larger than the previously known lp5’s and has no gene content other than a PFam57 gene in common with them (its map is shown in Fig. S3). Although its compatibility relationship to the canonical lp5’s is not known, we provisionally call this plasmid “lp5” until a more biologically informative classification system is devised for such plasmids. We note that other linear plasmids with only a PFam57 partition gene are also present in non-Bbsl species *B. miyamotoi* (70) and the relapsing fever clade (71).

### Horizontal exchange of homologous DNA within and between Bbsl species

Horizontal DNA exchange of genetic material is a major source of genome diversity in bacteria that can interfere with the accurate determination of phylogenies (72–74). To increase the accuracy of Bbsl global and local phylogenies, two aspects of the studies reported here are significant improvements over previous studies: the number of genomes and species they represent is substantially increased, and genomic regions most strongly affected by the past horizontal exchange of homologous sequences were identified and excluded from the phylogenetic analyses discussed below. To facilitate such analyses, BCFTools and VCFTools (75) were used to identify bi-allelic single-nucleotide variants (SNVs) in the chromosome and plasmids cp26, lp54, and lp17 of the 78 Bbsl genomes listed in Table S1A and B. The SNVs were mapped individually to the homologous reference B31 core replicons using a BWA aligner (76) (see METHODS). These SNVs by definition reside in homologous regions that are present in all of the sequences being compared and so their use ignores gene losses and gains and other indels; these loses and gains are considered in the “Plasmid dynamics” section below. Intra-species exchange of homologous sequences is called “recombination” here, and inter-species exchange is called “introgression.”

#### Intra-species recombination

The SNVs identified above were used to estimate the rates of intra-species genetic exchange of homologous sequences among 28 currently available *B. burgdorferi* genome sequences (Table S1A and B). Intra-specific recombination analysis requires a comparison of a number of genomes coexisting in the same populations, so it can only be performed on *B. burgdorferi* at present. LDhat, which detects recombination between pairs of bi-allelic SNVs based on the presence of four-gamete genotypes (77), found plasmid-specific rates of recombination (*ρ* = *2* Nr, where *N* is the effective population size and *r* is the per-site recombination rate), with the highest average recombination rates on lp54, and the lowest on the lp17 constant region (Fig. S4A right panel). Notably, recombination occurs on the main chromosome at a rate comparable to the plasmids. In addition, the LDhat analysis identified sites with elevated recombination rates (*P* < 2.2×10^−16^ by *t*-test) (Fig. S4A left panels). These 60 recombination hotspots include 48 on the chromosome, 6 on lp54, 1 on lp17, and 5 on cp26 (listed in Table S3). Recombination hotspots identified on the chromosome include a region surrounding the *bb_0158* locus that encodes the S2-like lipoprotein (78) and loci that encode flagellar proteins (*bb_0283*/*flgE* and *bb_0550*/*fliS*) (Fig.S4A[I]). The most prominent recombination hotspot on cp26 encompasses *bb_b17*, *bb_b19* (*ospC*) and *bb_b22* (Fig. S4A[II]). This finding is consistent with the results of an earlier analysis of 13 *B. burgdorferi* genomes (79). A single-nucleotide recombination hotspot was identified on lp17 at *bb_d21* (*parA*) (Fig. S4A[III]), and a single hotspot was identified on plasmid lp54 in the region surrounding the *bb_a24* (*dbpA*) and *bb_a25* (*dbpB*) loci (Fig. S4A[iv]). The *ospC* and *dbpA* genes are highly expressed during the initial invasion into the mammalian host and are highly polymorphic within natural populations (44, 57, 80).

**Fig 3:**
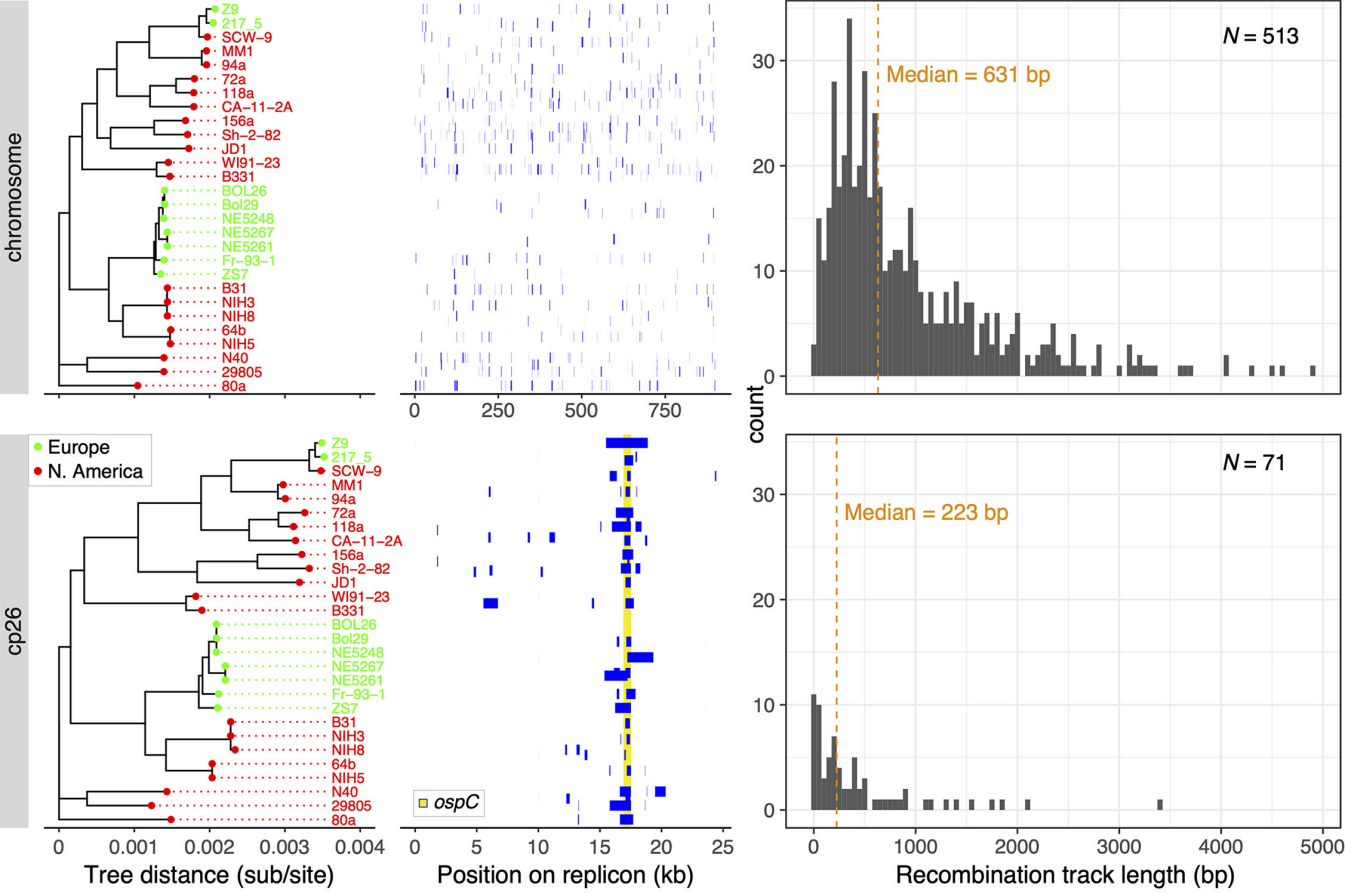
Intra-specific recombination. Recombination tracks (in blue) were identified on the main chromosome (top) and cp26 plasmid (bottom) in 28 *B. burgdorferi* genomes (as well as in hypothetical genomes represented by the internal nodes) using ClonalFrameML (81). Both trees were based on the maximum-likelihood tree reconstructed using the chromosomal SNVs (see Fig. 4). The density plots (right) show the length distribution of the recombination tracks. Recombination occurs across the chromosome, while *ospC* and *dbpA* (not shown) are recombination hotspots on cp26 and lp54, respectively, consistent with the results of LDhat analysis (Fig. S4A).

We also analyzed the *B. burgdorferi* intra-specific SNV data sets with ClonalFrameML (81), which is specifically tailored to the analysis of bacterial genome recombination under the assumption of a predominantly clonal population structure (see METHODS). Consistent with the results of LDhat analysis, ClonalFrameML analysis showed *ospC* and *dbpA* as recombination hotspots on cp26 (Fig. 3) and lp54 (not shown), respectively. By contrast, ClonalFrameML found no similarly prominent recombination hotspots on the chromosome (Fig. 3). Furthermore, this analysis estimated the distributions of recombination track lengths, as well as the recombination rates (*ρ = 2* Nr) relative to the mutation rates (*θ = 2Nμ*), where *N* is the effective population size and *r* and *μ*, are recombination and mutation rates, respectively (Fig. 3). The average recombination to mutation ratios (*ρ/θ*) are 0.426, 0.364, 0.606, and 0.462 for the chromosome, lp17, cp26, and lp54, respectively. These recombination rates result in the proportion of recombinant polymorphisms (*r*) that is either higher than or on par with mutational polymorphisms (*m*) (*r/m* = 1.21, 3.28, 3.25, and 0.792 for the chromosome, lp17, cp26, and lp54, respectively). Thus, the LDhat and ClonalFrameML tests showed that intra-species recombination occurs about as frequently as a mutation on both the chromosome and plasmids, while recombinant alleles are preferentially retained at these two lipoprotein-encoding loci. Previously, an estimated *ρ/θ* = 0.182 was obtained with ClonalFrameML based on a set of 114 orthologs of single-copy orthologous genes on the main chromosome from 111 Bbsl strains encompassing 14 named species (82). This latter estimate may be considered an average of both between-species and within-species recombination. By contrast, here the estimates were based on more replicons, more genes, and 28 strains within a single species. Both geographic segregation and increased genetic distances between species reduce recombination rates (53, 83).

#### Inter-species introgression

To detect genetic exchange (“introgression”) between co-existing North American Bbsl species, we calculated the *D*-statistics (also known as the ABBA-BABA test) at individual gene loci using the software Dfoil (84). This test examines phylogenetic patterns at biallelic SNV sites based on ancestral and derived alleles in four closely related genomes. Dfoil was used to examine inter-species exchange between *B. burgdorferi* (“P1”) and the other North American species (“P3”), with the European *B. finlandensis* isolate SV1 (“P2”) and South American isolate VA1 (“O”) homologs as sister- and out-groups, respectively (see METHODS). The four-allele patterns ABBA and BABA are expected to occur at the same frequency (*D* = 0) due to the random sorting of ancestral alleles into descendant P1 and P2 lineages (85). An excess of either four-allele pattern indicates cross-species gene flow and its direction: P1 toward P3 for genes showing *D* < 0 and P3 toward P1 for those with *D* > 0. We found no significant differences between the average *D* values for the chromosomes (*D* = 16.2) and the plasmids (cp26, *D* = 1.4; lp54, *D* = 16.3; and lp17, *D* = 17.7). The generally positive *D* scores indicate that the *B. burgdorferi* genomes have experienced significant introgression from other North American species. We chose *B. burgdorferi* as the focal species for the above introgression analysis because it has the greatest number of genomes available and is also the most common species in North America. Future studies will be able to estimate the level and rate of introgression in other species as more conspecific genomes become available.

The introgressed regions identified by the above analysis are listed in Table S4. Selected regions identified as introgressed were validated by an examination of sequence alignments followed by an independent statistical analysis based on an excess of homoplasies. For example, introgression was previously inferred at the chromosomal *bb_0082* locus by the homoplasy test (55). Loci involved in inter-species introgression are expected to show consecutive runs of SNVs that are incompatible with simple linear descent from a common ancestor (i.e*.,* are homoplastic) (6, 86). An alignment of a section of the *bb_0082* chromosomal locus displays such phylogenetic inconsistencies that were previously identified as an introgression event that originated from a *B. bissettiae*-like strain into the *B. burgdorferi* N40 lineage (55). However, the newly sequenced genomes allow a more precise determination that the donor was a *B. americana* strain represented by the SCW-30h and SCW-41 genomes (Fig. S4B). In this region, the N40 sequence differs at six nucleotide sites from homologous sequences in conspecific isolates, while there are only 0 or 1 differences between N40 and its homologous sequence in the *B. americana* strains. The four-taxon *D*-statistics analysis successfully identified this inter-species exchange (*P* = 1.57×10^−14^) from the *B. americana* SCW-30h lineage to the N40 *B. burgdorferi* lineage (Table S4). Furthermore, specific examples of intra- and inter-specific DNA exchanges are evidenced by gene-tree and genome-tree inconsistencies, as shown below.

### Genome phylogenies and biogeographic histories of Bbsl species

#### SNV trees

The overall accuracy of bacterial phylogeny determinations based on whole-genome sequences is improved if sites of frequent horizontal exchange of genetic information are removed (74). Therefore, sites identified above as having high intra-specific recombination or inter-specific introgression were excluded from the SNV alignments in subsequent phylogenetic analyses (see METHODS). Although high recombination and introgression sites could not be determined for all species, this nonetheless serves to significantly lower interference by such sites.

**Fig 4:**
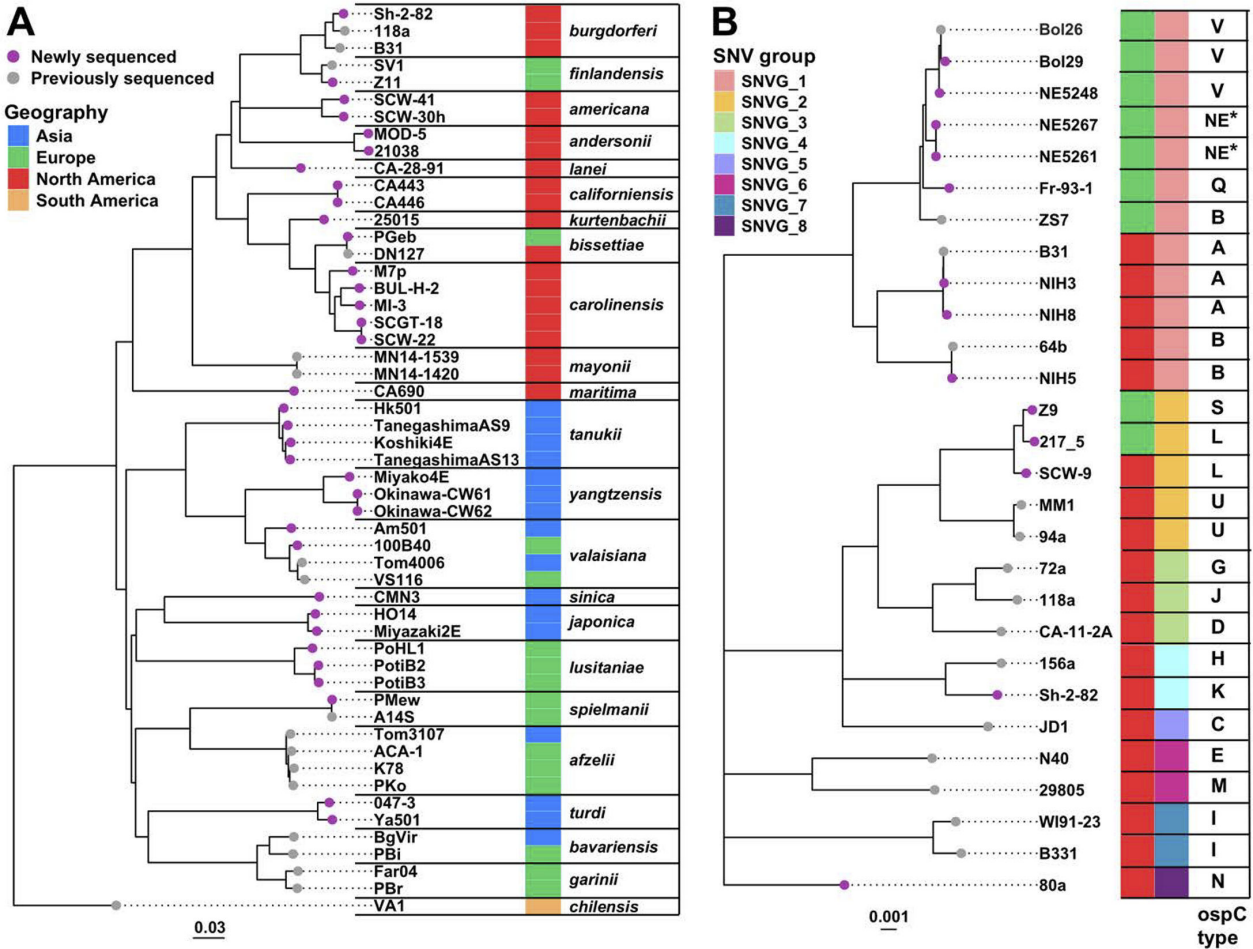
Comprehensive chromosome phylogeny of the *Bbsl* species. (**A**) Phylogeny of sequenced chromosomes from 23 species. The maximum-likelihood tree was constructed with IQ-Tree (87) using the alignment of the concatenated SNVs identified in the *Borreliella* chromosome after excluding the loci identified as subject to significant recombination and introgression as well as the unique terminal extensions as described in the text and Materials and Methods. Although they were included in the tree’s construction, to simplify presentation not all of the previously available genomes are shown in this tree. The tree was rooted using the chromosome of *B. chilensis* isolate VA1 according to the previously published genome trees (47). All branches are supported by bootstrap values of 80% or higher. The branch tips are colored according to genome sequencing status (presented here or previously sequenced). The heatmap column to the right of this panel indicates the continental origins of the sequenced strains. Species designations of *Borreliella* strains are shown on the right. (**B**) Phylogeny of 28 sequenced *B. burgdorferi* chromosomes. The expanded *B. burgdorferi* branch of the IQ-Tree SNV tree is displayed. It includes all 28 currently known complete *B. burgdorferi* genomes. The first and second heatmap columns on the right indicate the continental origins and the within-species SNV groups (clustered by the algorithm described in Materials and Methods), respectively; the rightmost column gives the OspC type.

To analyze the complete Bbsl phylogeny, both maximum likelihood and multispecies coalescent methods were used. The alignment of the 164,100 concatenated chromosomal SNVs was used to construct a maximum likelihood phylogeny of all 78 Bbsl genomes in Table S1A and B using IQ-Tree (87). The resulting SNV tree robustly supports the separation of the Bbsl group into the 23 existing species (Fig. 4A). With a few exceptions that suggest intercontinental dispersal events (see below), the chromosomal tree—with more than twice as many species as previous genome-based trees—strongly confirms the division of the Bbsl genus into two clades associated primarily with Eurasia and North America (see “Historic dispersal of Bbsl Species” section below for further details). This robust and comprehensive phylogeny is largely consistent with previously published relationships based on substantially fewer genomes (16, 44, 55, 56, 88, 89) and places 13 additional species in the tree.

The most closely related pairs of species are *bissettiae-carolinensis, burgdorferi-finlandensis,* and *garinii-bavariensis*. Among the genomes in Fig. 4A, those of *B. yangtzensis* Miyako4E, *B. valaisiana* Am501, and *B. lusitaniae* PoHL1 are most distantly related to other members of their species. Should they be given species status? None of these is as distant from other members of its current species as the separation between the above-mentioned closely related “sister” species. Thus, their positions in the tree do not seem to warrant new species status at this time, even though *B. lustitaniae*, for example, appears to have two geographically distinct populations (54, 90).

Maximum likelihood trees of plasmids cp26, lp54, and lp17 were also inferred using the recombination-minimized SNV alignments described above. The cp26, lp54, and lp17 phylogenetic plasmid trees shown in Fig. S5 to S7 are broadly similar to one another and to the chromosomal tree (exceptions are discussed below), and thus provide substantial additional support for the division into Eurasian and North American clades. In addition to these major continental clades, all four SNV trees show essentially the same subclades. The Eurasian clade is made up of five subgroups: *sinica-japonica*, *afzelii-spielmanii*, *valaisiana-yangtzensis-tanukii,* and *garinii-bavariensis-turdi*, while *lusitaniae* forms its own subgroup. The North American clade is made up of four subgroups: *burgdorferi-americana-lanei-andersonii-finlandensis* and *carolinensis-californiensis-bissettiae-kurtenbachii*, while *mayonii* and *maritima* each form their own subgroup.

The larger number of *B. burgdorferi* isolates that have sequenced genomes makes analyses of the substructure within this species robust and informative. Previously, four subtypes within this species were defined by genome-wide SNV analysis (44). Here, with substantially more genomes, we re-define eight such genomic groups (SNVG1 through 8) based on the intra-specific phylogeny of *B. burgdorferi* (Fig. 4B) and delineated by a tree-trimming algorithm that identifies leaf nodes within a cutoff distance of 0.26% substitutions per site from their common ancestor as a single group.

#### Multispecies coalescent tree

**Fig 5:**
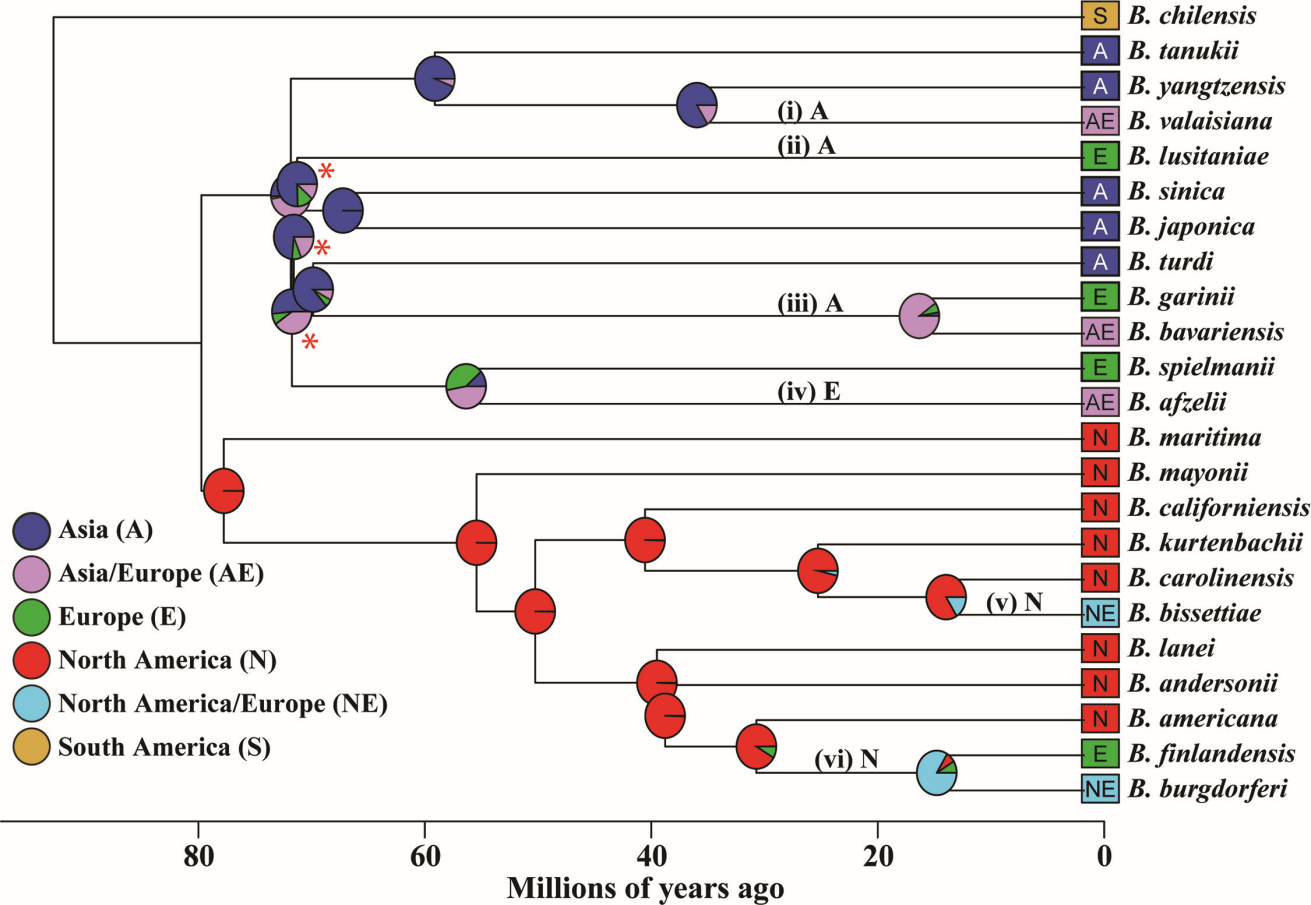
*Bbsl* species inter-continental dispersal and crude divergence times. The timed phylogeny of 23 *Bbsl* species’ chromosomes was estimated using BPP with a multi-species coalescence model (93) as described in Materials and Methods. The colors of the squares at the branch tips on the right indicate the geographic origins of the isolates. The ancestral state reconstructions of species’ geographic origins were obtained using BioGeoBears (94). Except for the two deepest nodes where geographic origins are poorly resolved, colored wedges in the pie charts at the internal nodes show probabilities of ancestral geographic origins. Six major intercontinental dispersal events—on branches where ancestral probabilities show the greatest differences between the child and parent nodes—are labeled with Roman numerals (i through vi) and N, A, and E indicating North American, European, and Asian origins, respectively. All branches have posterior probability support of >0.95, except for the three marked by red asterisks (with 0.8 > branch support > 0.65).

We also estimated a Bbsl species phylogeny using a multispecies coalescent model. This model can account for intra-species recombination events as well as stochastic inconsistencies between the gene trees and the underlying species phylogeny (91, 92). For this purpose, we assembled a data set consisting of 304 individually aligned chromosomal gene sequences (see Data Availability). A maximum Bayesian posterior maximum clade credibility (MCC) species tree based on a multispecies coalescent model was generated using the software Bayesian Phylogenetics and Phylogeography (BPP) (Materials and Methods) (91, 93). This BPP tree is shown in Fig. 5; it identifies the 23 species with >95% posterior probability except for three branches with posterior probability support values <80%. No significant topological inconsistencies were observed between the chromosomal SNV phylogeny inferred by IQ-Tree (above) and the species MCC tree inferred by BPP.

### Historic dispersal of Bbsl species

BioGeoBEARS (1) analysis based on the best-fitting model [the Dispersal-Extinction-Cladogenesis with jump dispersal at speciation (DEC + J) model; Table S5B] and the BPP phylogeny (above; Materials and Methods) was used to reconstruct the historical geographic spread of Bbsl species. The results suggest six major dispersal events after the initial geographic separation of the Eurasian and North American clades (Fig. 5). In the North American clade, several of these dispersal events occurred between North America and Europe, similar to the conclusions of previous studies of European and North American *B. burgdorferi* isolates (51, 52, 66). Current European populations of *B. burgdorferi*, *B. bissettiae,* and *B. finlandensis* are all part of the ancestrally North American clade and thus represent independent dispersal events originating from North America (Fig. 5, branches labeled (v) and (vi)), in spite of the fact that *B. burgdorferi* and *B. bissettiae* isolates are currently found on both continents and *B. finlandensis* isolates have only been found in Europe. This analysis indicates that European *B. burgdorferi* lineages descended from the SNVG1 subgroup well after *B. burgdorferi* diversification in North America (Fig. 5). By contrast, *B. finlandensis* is a sister group of *B. burgdorferi* and represents an ancient introduction to Europe that predates the diversification of *B. burgdorferi* in North America. Initial analyses of North American and European *B. burgdorferi* and *B. finlandensis* isolates, using MLST without the use of other North American species, suggested a European origin of these species (52, 66). A recent biogeographic study of *B. burgdorferi* based on whole-chromosome sequences but without the use of isolates representing extensive North American species diversity indicated multiple trans-Atlantic dispersal events but did not resolve the geographic origins of this major human-pathogenic species (95). Previously, a phylogeographic study of 14 named Bbsl species inferred migration events but was based on 114 chromosomal orthologs from un-assembled short reads (82). The results presented here, based on the fully assembled genome sequences of isolates covering global inter- and intra-species diversities, should be considerably more robust than the previous analyses.

Phylogeographic reconstruction also supports an East Asian origin of the Eurasian clade (Fig. 5), in broad consistency with results of a recent study concluding Asian origins of European lineages of *B. garinii* and *B. bavariensis* (branch labeled (iii)), both of which are a part of the ancestral Asian clade (88). This geographic reconstruction suggests additional ancestral migration events from Asia to Europe, which gave rise to *B. valaisiana* (i)*, B. lusitaniae* (ii), and *B. afzelii* (iv). Back migrations have occurred from Europe to Asia as well, giving rise to Asian lineages of *B. valaisiana* (i) and *B. afzelii* (iv).

### Plasmid dynamics

The cp26, lp54, and lp17 plasmids from all 23 Bbsl species generally form three highly syntenic groups, but there are a few exceptions. When the plasmid and chromosome SNV trees are compared, several major phylogenetic tree inconsistencies are present. These are discussed in the following sections.

#### Cp26

In terms of gene synteny and content, the cp26’s are the least variable of the Bbsl plasmids, with only the following few examples of imperfect synteny among known genomes, all of which were discovered in the newly sequenced genomes presented here. (i) *B. andersonii* 21038 and MOD-5, as well as (ii) *B. sinica* CMN3 cp26 plasmids have apparently debilitating deletions in their *guaA* and *guaB* genes (Fig. S8), suggesting that they occupy niches in which this purine metabolism is not important. (iii) Both *B. turdi* Ya501 and 047–3 cp26 plasmids have an apparent transposon insertion about 2,500 bp long between genes *bb_b14* and *b16* (strain B31 names) that contains degraded fragments of an IS605 OrfB transposase gene. (iv) Finally, all three *B. lusitaniae* cp26 plasmids are circular dimers of a “normal” cp26 with two divergent and partially degraded halves (details to be described elsewhere). There are no convincing inconsistencies between the Bbsl cp26 and chromosome trees, and within *B. burgdorferi,* the chromosome and cp26 trees are strikingly similar (Fig. S9). Thus, there is no evidence for past whole plasmid transfer of cp26 between species, although genetic exchange involving *ospC* is not unusual (Fig. 3; see the OspC discussion below).

#### Lp54

The lp54 plasmids are also all essentially syntenic with the following major exceptions discovered in the newly sequenced plasmids: (i) Like the chromosomes (above), lp54 plasmids have constant central regions but can have a variety of terminal extensions that carry other plasmid-like genes. Figure S10 shows that these extensions are usually unique to the species or clade of species that bear them and are usually (but not always) quite similar in different isolates of the same species. (ii) *B. lusitaniae* lp54 plasmids are missing the rightmost gene *thyX* (96); the right telomere is present in these sequences so this is not a failure of sequence assembly to reach the plasmid end. A *thyX* gene is present in all other species except *B. japonica* (see below); however, we note that *thyX* is a damaged pseudogene in the *B. americana* and *B. turdi* genomes that were sequenced. (iii) All three *B. lusitaniae* lp54 plasmids have a quadruplication of the *bb_a36 gene*. (iv) *B. sinica* CMN3 lacks orthologs of *bb_a60* and *a61*. (v) The *B. tanukii* isolates lack an ortholog of the B31 *bb_a65* gene in the “constant” portion (see below) of the PFam54 array. (vi) Numerous gene losses and gains have occurred in the variable portion of the lp54 PFam54 array [Clade IV in reference (97)] that are discussed in more detail below in the “CspA and PFam54 array proteins” section. (vii) The *B. japonica* lp54 plasmids have a unique structure that has been experimentally documented in HO14 (98). In isolates HO14 and Miyazki2E, the left majority portion of a “normal” lp54 has been duplicated as a long head-to-head inverted repeat linear dimer with two “left” ends. The HO14 and Miyazki2E lp54s are thus missing genes normally in the right portion of the PFam54 array (homologs of B31 *bb_a66* through *bb_a74* and *bb_a67* through *bb_a74*, respectively), as well as the right-end genes *oms28* (99) and *thyX* (96), and they have 78 and 24 bp of non-duplicated DNA between the two halves of the inverted repeat, respectively (shown diagrammatically in Fig. S11). This lp54 structure is likely a characteristic of all *B. japonica* isolates since the only other isolate of this species that has been examined, IKA2, has a similar, unusually large lp54 (98). This type of inverted repeat plasmid structure is not limited to these lp54 plasmids; we have found that the following non-core “variable” plasmids also have such a whole-plasmid inverted repeat structure: lp28-2 plasmids in *B. burgdorferi* strains NE5248, NE5261, and NE5267, the lp36 plasmids in *B. burgdorferi* strains 80 a and 118 a, and lp28-7 in *B. andersonii* 21038. All of the above rearrangements are species-specific and, as such, almost certainly occurred within species lineages.

Chromosome SNV tree-lp54 SNV tree inconsistencies suggest that major DNA transfers involving these plasmids appear to have occurred as follows: (i) The *B. lanei* CA-28–91 lp54 is quite closely related to those in *B. mayonii*, whereas the chromosomal tree places *B. lanei* CA-28–9 in the subclade that includes *B. burgdorferi* (*cf*. Fig. 4A; Fig. S6). This inconsistency suggests that horizontal genetic exchange of possibly the whole lp54 plasmid occurred between *B. lanei* and *B. mayonii*. (ii) The *B. lusitaniae* lp54s are more closely associated with the North American clade than are their chromosomes, but the deep branches involved make it less certain that this indicates plasmid exchange. (iii) In addition, there are at least six lp54-chromosome inconsistencies within the *B. burgdorferi* portion of the trees (marked by red stars in Fig. S12), suggesting more frequent partial or whole plasmid exchange of lp54 DNA within species than between species.

#### Lp17

Although it is present in essentially all Bbsl isolates (above), linear plasmid lp17 is a “partly variable” plasmid that has an ~11.5 kbp “constant region”; however, in different isolates, it has different other-plasmid-like sequences fused to the left end of this constant region [see analyses of previously sequenced lp17s in Fig. 3 of Casjens et al. (49) and Fig. 4 of Casjens et al. (45)]. We note that although these extensions are very variable, they are not all unrelated; for example, nine of the 34 newly sequenced non-*B*. *burgdorferi* genomes carry homologs of the fibronectin-binding protein-encoding *B. burgdorferi* B31 plasmid lp36 gene *bb_k32* (100, 101) in different contexts in their lp17 left end extensions (*B. americana* SCW-30h, *B. carolinensis* MI-3 and M7p, *B. kurtenbachii* 25015, *B. spielmanii* PMew, *B. turdi* Ya501 and 047-3, and *B. yangtzensis* Okinawa-CW62 and -CW61). When multiple genomes are known for a given species, they typically have several different lp17 left-end variations; for example, among the genomes analyzed here, the two genomes of *B. bissettiae* (DN127 and PGeb), *B. finlandensis* (SV1 and Z11), and *B. americana* (SCW-41 and SCW-30h) have quite different left-end regions in each case. On the other hand, there are a number of cases where nearly identical lp17s are present in independent isolates of a given species, for example, *B. californiensis* CA443 and CA446, *B. carolinensis* M7p and MI-3, as well as *B. tanukii* Koshiki4E, TanegashimaAS9, and TanegashimaAS13. Thus, there appears to be limited number of such “organizational subtypes” in each species, with little overlap among species (see the single exception below). The previously known 15 *B. burgdorferi* lp17s (Table S1B) carry 7 such organizational subtypes (45), and the 13 new *B. burgdorferi* lp17 sequences reported here (Table S1A) fall into these categories except that of isolate 80a, which forms an eighth subtype (Fig. S13). The finding of only one new subtype, while nearly doubling the number of isolates and even more substantially increasing genetic and geographic diversity in the strain panel, suggests that there may be few, if any, additional *B. burgdorferi* lp17 organizational subtypes that have not been identified. This gives an idea of the extent of this kind of diversity.

The lp17 SNV tree (Fig. S7A) points out a clear example of interspecies horizontal lp17 exchange. The *B. spielmanii* A14S and PMew lp17 plasmids are nearly identical to each other and to the lp17 of *B. afzelii* K78, one of the four known *B. afzelii* lp17 organizational subtypes [Fig. 3 of Casjens et al. (49)]. The currently most parsimonious explanation for these relationships is the past transfer and replacement of a whole *B. spielmanii* lp17 into the K78 *B. afzelii* lineage (but not into other *B. afzelii* lineages).

To deduce the history of the *B. burgdorferi* lp17s more robustly, we extracted their constant regions (homologous to bp 4546–15961 of B31 lp17) to remove any anomalous contribution to the analysis by the variable ends and used IQ-Tree to build a maximum likelihood SNV tree (Fig. S7B). Figure S14 compares this tree with the chromosome tree and shows that within the *B. burgdorferi* species, (i) the constant region of lp17 has diverged in parallel with the chromosome with no evidence for large-scale horizontal exchange and (ii) recombination between the constant region and the left-end extension has not been found. These results suggest that the lp17 left-end extensions formed rather long ago (but after species divergence) and have been modified over time since then. The fact that the *B. burgdorferi* lp17 left-end extensions correlate perfectly with the branches of the lp17 constant region tree (Fig. S14) indicates that they formed before the divergence of these intraspecies clades.

In addition, the novel sequence joints at the boundary between the constant region and the extension (the apparent site of original extension addition compared to B31, for example) within a species are often the same even though there can be more terminally located differences; this supports a single original addition event followed by modification and/or partial replacement of the added DNA. The latter is true in the following lp17 intra-species sets: *B. afzelii* ACA-1, PKo, and K78; *B. lusitaniae* PotiB2, PotiB3, and PoHL1; *B. finlandensis* Z11 and SV1; *B. bissettiae* DN127 and PGeb; *B. valaisiana* VS116, Am501 and 100B40; *B. yangtzensis* CW16, CW61, and Miyako4E; *B. turdi* Ya501 and 047–3; *B. japonica* HO14 and Miyazaki2E. These *B. bissettiae* and *B. valaisiana* relationships are shown in Fig. S15, where geographically distant isolates from California and Germany plus Germany and Japan, respectively, have the same original novel joints.

We previously noted that lp17s in four European clade species have an approximately 5 kbp inversion in their constant region relative to *B. burgdorferi* [see Fig. 3 in Casjens et al. (49)]. The 46 lp17 plasmids sequenced in this report confirm this relationship for 13 additional species with one exception. *B. maritima* CA690 lp17 has the “European” inversion orientation. *B. maritima* represents the most ancestral lineage of the North American clade in the chromosome tree and the cp26 and lp54 plasmid SNV trees (Fig. 4; Fig. S5 to S7). The most parsimonious interpretation is that the inversion happened in the North American clade just after the divergence from *B. maritima*, which retained the ancestral state.

#### Plasmid fusions

Another form of rearrangement is the fusion of different whole plasmids. One clear type is fusions of whole cp32 plasmids (~30 kbp) that were apparently merged by homologous recombination to form dimer circles (~60 kbp). These have been discussed previously (40, 45, 102). Fusions also include the integration of circular plasmids into linear plasmids, such as the previously noted integration of whole cp32 circles into an lp56 and into an lp54 in isolates B31 and SV1, respectively (38, 49). Among the genomes presented here, we discovered a whole cp9 integrated into an lp21 plasmid in *B. burgdorferi* isolate NIH8. Forty-four of the 607 plasmids reported here carry more than one partition gene cluster that includes apparently functional PFam32 genes of different types. A majority of these appear not to be neat whole plasmid fusions between known plasmids. They are either the result of the transfer of part of one plasmid to another or the fusion of whole plasmids followed by further deletions and rearrangements.

### Accelerated evolution of surface lipoproteins

**Fig 6:**
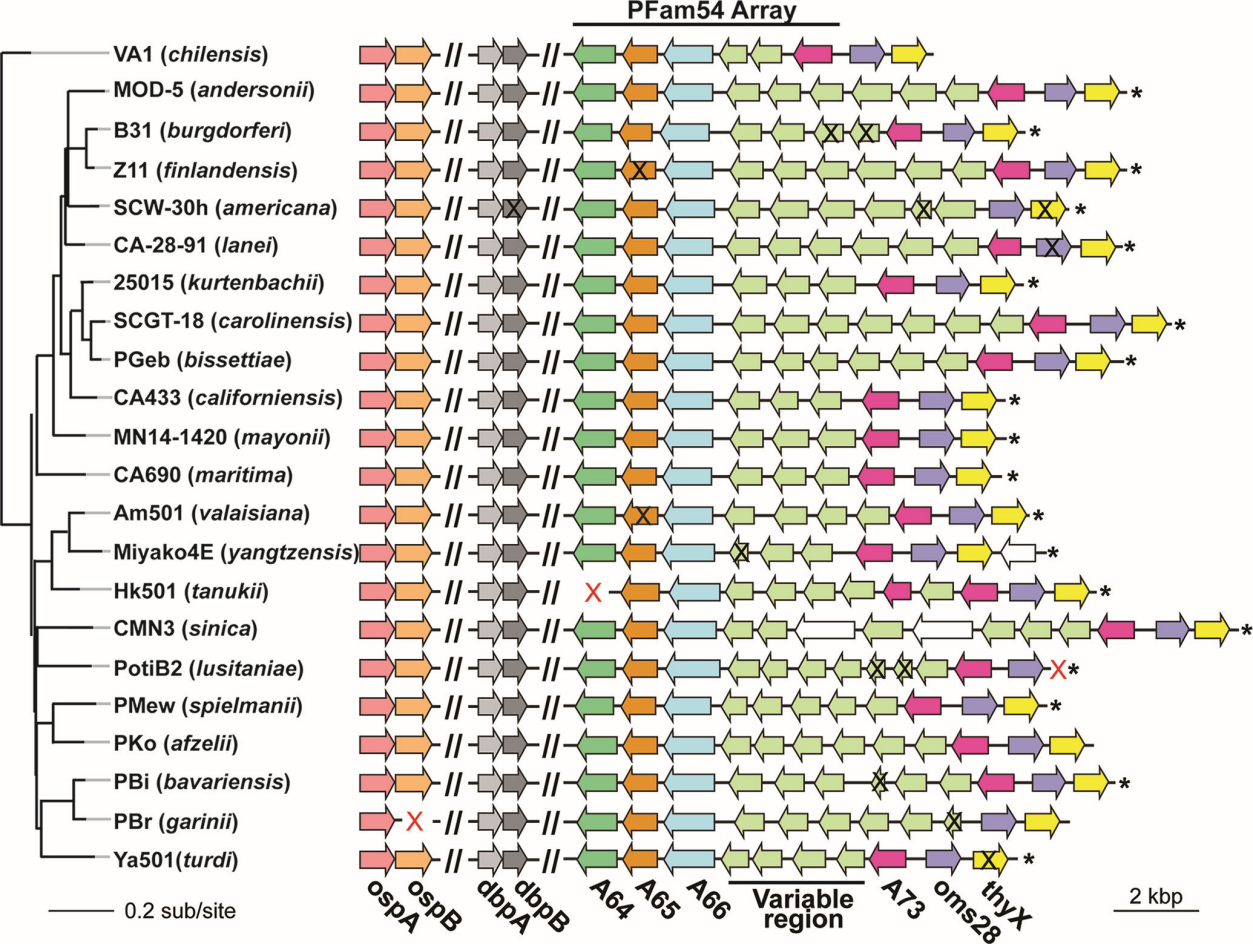
Gene duplication and losses on the linear plasmid lp54. Selected plasmid lp54 genes are shown as arrows. The rows are arranged according to the chromosomal SNV phylogeny tree (see Fig. 4). The left-end region (see Fig. S10) and conserved internal syntenic regions are omitted (“//”). One representative genome from each species is shown. Each arrow represents an open reading frame, the arrowhead depicts the transcription direction. Orthologous gene sets, defined by gene phylogenies, are unique colors except for variable region genes which represent multiple orthologous sets but are all light green. Part of the PFam54 array and the canonical lp54 right end have been lost in *B. japonica* so its lp54 is not shown in the figure (see text and Fig. S11). Asterisks mark right termini in which the sequence includes the right telomere; terminal gene presence/absence is uncertain in the cases without asterisks. Black “X”s mark pseudogenes (deduced from their apparent truncation), and red “X”s indicate missing genes.

Genome phylogenies inform not only species evolution but also gene evolution and function. Here we analyze the gene evolution of three key host-interacting loci—*ospC* on the cp26 plasmid and *dbpA* and the PFam54 gene array [non-universal Clade IV homologs (97)] on the lp54 plasmid. With one exception (*B. japonica* in PFam54 section, see above), all three gene families are present in all species in this genus. Similar comparative analysis is ongoing and pending the complete analysis of the hypervariable lipoprotein loci residing on replicons other than the chromosome, lp17, cp26, and lp54, for example, the multi-copy *vls* genes (103). The *ospC* gene is essential for tick salivary gland invasion or host colonization and is expressed at the initial stage of mammalian infection (80, 104–107). OspC is the most variable single-locus protein in the Bbsl proteome and the serotype-determining antigen of Bbsl strains (57, 67, 79, 108). The tight genetic linkage between major *ospC* alleles and genome lineages makes *ospC* a key clinical marker for invasive Lyme disease (52, 109–111). The *dbpA* locus encodes a decorin-binding protein and is, like *ospC*, expressed early during host invasion (108, 112). Sequence variation of DbpA is associated with tissue specificity (113). Near the right end of lp54 is a tandem array of genes whose protein products are members of the PFam54 family (97) (Fig. 6). One of these PFam54 genes, *cspA* (also called *bb_a68*), encodes a protein that inhibits host complement-mediated killing by binding the complement regulatory protein Factor H (114, 115). CspA sequence variability has been associated with host specificity among Bbsl species (116). Below, we reconstruct gene trees using the protein sequences for these three loci and compare them to the genome-based phylogeny. Significant inconsistencies between a gene and a species tree—in tree topology and/or tree distance—are indicative of non-phylogenetic processes (migration, horizontal gene transfer, and recombination) and may reflect selective advantages (74, 83, 117). In addition, phylogenomic analysis based on the reconciliation of gene and species trees helps inference of gene functions by distinguishing between paralogous and orthologous genes (118–120).

We designed a “treemap” display to aid the combined analysis of gene and species trees. First, we consolidated the gene tree by trimming the branch tips based on tree distances [with the “biotree” utility of the BbWrapper software package (121); see Methods], thereby reducing the total number of branch tips. Second, we sorted strains according to both the gene tree (in rows) and the species tree (in columns). The treemap display has the benefit of minimizing the gene tree size by highlighting major antigen variants while listing all genomes containing a gene variant. The treemap also allows visual identification of phylogenetic inconsistencies, achieved under the evolutionary expectations that closely related species should harbor similar gene variants and, *vice versa*, that similar gene variants should be found in closely related species. As described below, departures from these phylogenetic expectations enabled us to confirm dispersal events, reveal horizontal gene transfers and gene duplications, and identify orthologous genes.

#### OspC

**Fig 7:**
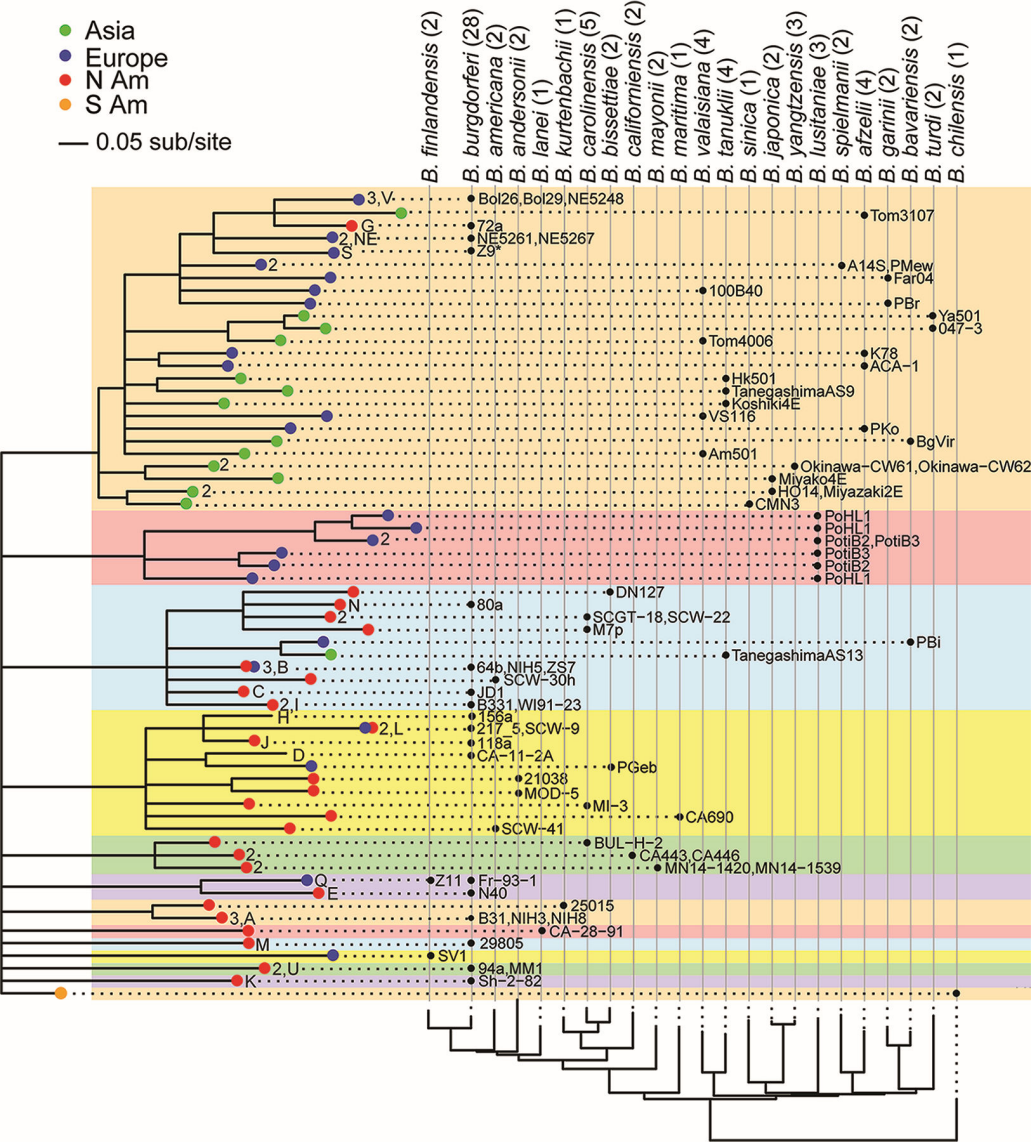
Major OspC variants from sequenced genomes. On the left, the maximum likelihood tree was constructed by IQTree and consists of a set of all 82 OspC sequences from fully sequenced genomes (47 from the present study). Branches with less than 90% bootstrap support values are collapsed. Branch tips (*n* = 64) with ≤0.05 amino-acid substitutions per site were merged (see tip-consolidating algorithm described in Materials and Methods) and are colored according to the continents where the isolates carrying them were found. Where there is more than one allele in the branch, numerals at the tip indicate the number of alleles present in that branch. Letters at the tips indicate the *B. burgdorferi* OspC type (types have not been defined for the other species). In the right portion of the figure, OspC alleles are grouped in columns according to the species that carries them, as indicated by black circles with the names of the isolates that carry them to the right. Species are shown above with the number of isolates analyzed in that species in parentheses; the columns are ordered according to the chromosomal SNV tree shown below (see Fig. 4). Background color highlights the major OspC allelic lineages. The distance bar indicates the number of amino acid substitutions per site. Note that strains are listed once, except for the three *B. lusitaniae* strains, each of which contains two or three OspC alleles.

A total of 82 full-length OspC protein sequences from the newly and previously sequenced Bbsl genomes were used to infer a maximum likelihood tree with IQ-Tree (87). These were consolidated into a set of 64 OspC variant types whose sequences are separated by ≥0.05 amino-acid substitutions/site, a cutoff chosen to disregard variability within major-group alleles (57). In Fig. 7, OspC variants are sorted in rows according to their tree relationships, while isolates that harbor them are sorted in columns according to the chromosome tree in Fig. 4. The OspC proteins fall into 13 quite different phylogenetic groups indicated by colored background boxes in the figure that are 60%–80% identical to one another. Some of the OspC branches are geographically uniform and some are not. We note several points of interest as follows:

- *B. burgdorferi* European isolates Bol26, NE5621, Z9, and their relatives in the same branches in the upper part of the top orange box in Fig. 7 (OspC types S, V, and NE) have chromosomes in the *B. burgdorferi* European subclade (Fig. 4B) and have OspC types whose closest relatives are from Eurasian isolates of other species. The most parsimonious explanation for such a gene tree-species tree inconsistency is a single gene conversion event (followed by divergence) that gave these European strains their novel OspCs early after their ancestor migrated from North America. Presumably, the donor was a Eurasian strain in the lineage of *B. afzelii* Tom3107.
- Similar parsimony inference suggests that the OspC G variant in North American *B. burgdorferi* isolate 72a, whose chromosome is clearly in the North American subclade (Fig. 4B), may have originated from Europe or Asia. It could be that a Eurasian OspC allele was introduced to North America, after which its host strain went extinct. It might also be a case of convergent evolution between North American and Eurasian OspC variants. A third, more plausible possibility is that the OspC G variant represents an ancestral OspC variant in North America. Indeed, many European lineages (e.g., ZS7, 217_5, Fr-93-1, and Z11) have retained their North American OspC variants long after migrating into Europe. The notion that the OspC G variant is a deeply ancestral allele is supported by the fact that uniquely among all OspC variants, it is associated with all *ospA* alleles in northeast USA *B. burgdorferi* populations (58). Since genetic linkage among loci decays over evolutionary time, a lack of linkage of the *ospC* G variant with specific *ospA* alleles suggests its old age. Immunologically, the OspC G variant is the most specific (i.e., least cross-reactive) in reactions with human and mouse sera, consistent with its deep ancestry (122).
- The phylogenetic positions of the chromosomes of European isolates *B. bissettiae* PGeb, *B. burgdorferi* 217_5 (OspC type L), and *B. burgdorferi* ZS7 (OspC type B2, a subgroup of type B found only in Europe (66, 123, 124)) in Fig. 7 suggest a trans-Atlantic migration of these lineages from North America (above) while retaining their North American OspC ancestral variants.
- Eurasian isolates *B. tanukii* TanegashimaAS13 and *B. bavariensis* PBi (in central light blue box in Fig. 7) cluster with mostly North American OspC variants, suggesting their OspC origins were in North America.
- The close relationship between OspC B in North American *B. burgdorferi* isolates 64b and NIH5 to OspC B2 (above) in European *B. burgdorferi* isolate ZS7 indicate within-species transfer events that may have occurred recently in Europe.
- The *identical* type Q OspC variant shared by *B. finlandensis* Z11 and *B. burgdorferi* Fr-93-1 indicates a cross-species transfer event that also likely occurred recently in Europe.
- Uniquely, the three *B. lusitaniae* genomes each contain two or three *ospC* genes (shaded in pink in Fig. 7) due in part to the dimer structure of their cp26 plasmids (above). These OspCs form a self-contained branch with no predicted exchanges with other branches, indicating that the cp26 dimerization occurred in the common ancestor of this species (to be described in more detail elsewhere).

Overall, the treemap shows as much OspC sequence variability within-genome (in *B. lusitaniae*) as within-species and between-species, highlighting diversifying selection operating on this hypervariable lipoprotein that is driven by host immunity, host specificity, or both (57, 67, 79, 110).

#### DbpA

**Fig 8:**
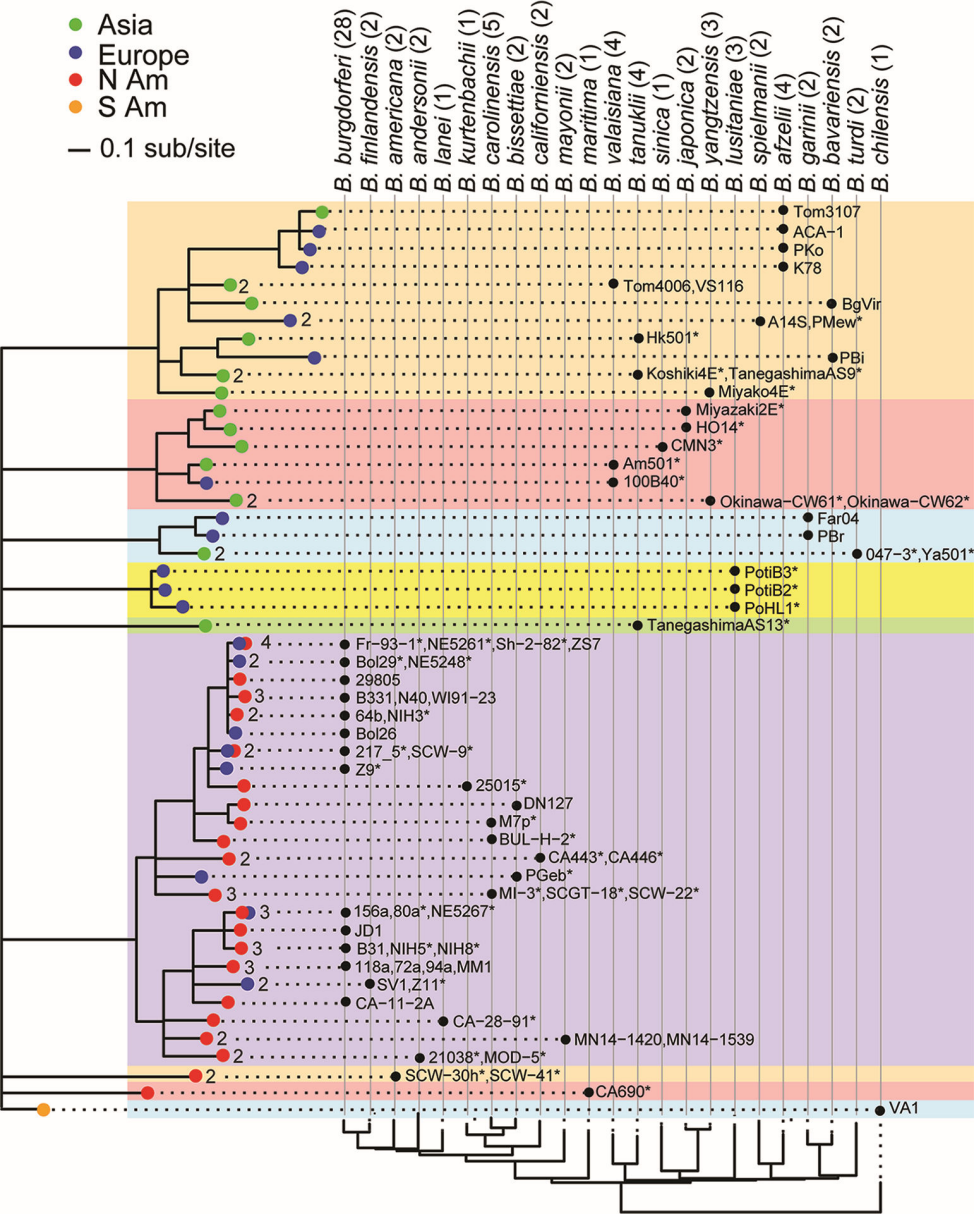
Major DbpA variants from sequenced genomes. The DbpA gene tree (left) consists of 52 non-redundant DbpA sequence types from 78 sequenced genomes. It was constructed and displayed as described in Fig. 7.

All Bbsl species carry paralogous *dbpA* and *dbpB* decorin-binding protein genes on lp54. However, in both *B. americana* SCW-41 and SCW-30h strains and *B. lusitaniae* PotiB3, the *dbpB* gene is a degraded pseudogene. Seventy-eight full-length DbpA protein sequences from the newly and previously sequenced genomes were used to create a maximum likelihood tree with IQ-Tree (87). The tree consists of 52 non-redundant variants after the tips were trimmed as described above (Fig. 8). These DbpA proteins fall into 13 very different phylogenetic groups with 40%–60% sequence identity and are indicated by colored background boxes in the figure. The gene tree at the left in the figure shows mostly well-resolved Eurasian clades (orange, pink, blue, yellow, and green backgrounds in the upper half of the tree) and North American clades (purple, orange, and pink in the lower half of the tree). Species are mostly restricted to a single major DbpA group, consistent with the results of an earlier study showing species-specific DbpA variants (125). Only *B. carolinensis, B. tanukii, B. valaisiana,* and *B. yangtzensis* isolates are split among multiple branches, and like OspC, DbpA is as variable within-species as between-species in these cases. On the other hand, most of the major DbpA clades/groups contain more than one species as follows: *B. afzelii, B. bavariensis,* and *B. spielmanii* reside in the topmost (orange) clade in Fig. 8; *B. japonica* and *B. sinica* reside in the pink clade; *B. garinii* and *B. turdi* reside in the upper blue clade; and finally, *B. andersonii, B. burgdorferi, B. bissettiae, B. californiensis, B. finlandensis, B. lanei, B. kurtenbachii,* and *B. mayonii* are all contained within the purple clade. The latter clade, unlike the other clades in Fig. 8, has interspersed isolates from Eurasia (twelve isolates) and North America (33 isolates). The Eurasian isolates include eight *B. burgdorferi*, one *B. bissettiae*, and two *B. finlandensis* isolates that carry chromosomes that clearly reflect independent North American ancestries (Fig. 5). There are three apparent chromosome-DbpA phylogenetic inconsistencies as very different DbpA allele types are present in different isolates of the same species as follows: (i) *B. valaisiana* (orange and pink), (ii) *B. yangtzensis* (orange and pink), and (iii) *B. tanukii* (orange and green). The TanegashimaAS13 OspC variant forms a more basal rather than a robustly Eurasian lineage, consistent with the phylogenetic position of the OspC variant from this strain and suggests a possible North American origin. Horizontal transfer of DbpA alleles between Bbsl species appears to be less common than transfer of OspC, although it is nonetheless an intra-specific recombination hotspot in *B. burgdorferi* (Fig. S4D).

#### CspA and PFam54 array proteins

*B. burgdorferi* PFam54 protein family contains a number of distantly related homologs that are found on several plasmids. For example, in *B. burgdorferi* B31, such genes reside on three different plasmids, lp28-4, lp38, and lp54. The PFam54 genes not on lp54 are more distantly related to the subfamily of genes in the PFam54 array on lp54. Lp54 plasmids carry from 6 to 11 such genes that lie in a single head-to-tail tandem array near its right end. The genes at both ends of the array (*bb_a64, _65,* and *_66* at the left end and *_73* at the right end) each form a set of conserved orthologs. Between *bb_a66* and *bb_a73* lie a variable number (2 to 7) of less conserved “variable region” PFam54 paralogs. This part of the PFam54 array does not show universal one-to-one orthology among all strains and is also known as the Clade IV homologs (97). One of these genes, *cspA* (*bb_a68*), has been studied and encodes a protein that inhibits host complement-mediated killing. Gene maps of these arrays are displayed in Fig. 6, where a selected member of each species is shown. *B. japonica* HO14 and Miyazaki2E do not have a complete PFam54 array (above), and the sequence of *B. carolinensis* M7p lp54 did not include the array, so these isolates are not included in this analysis.

**Fig 9:**
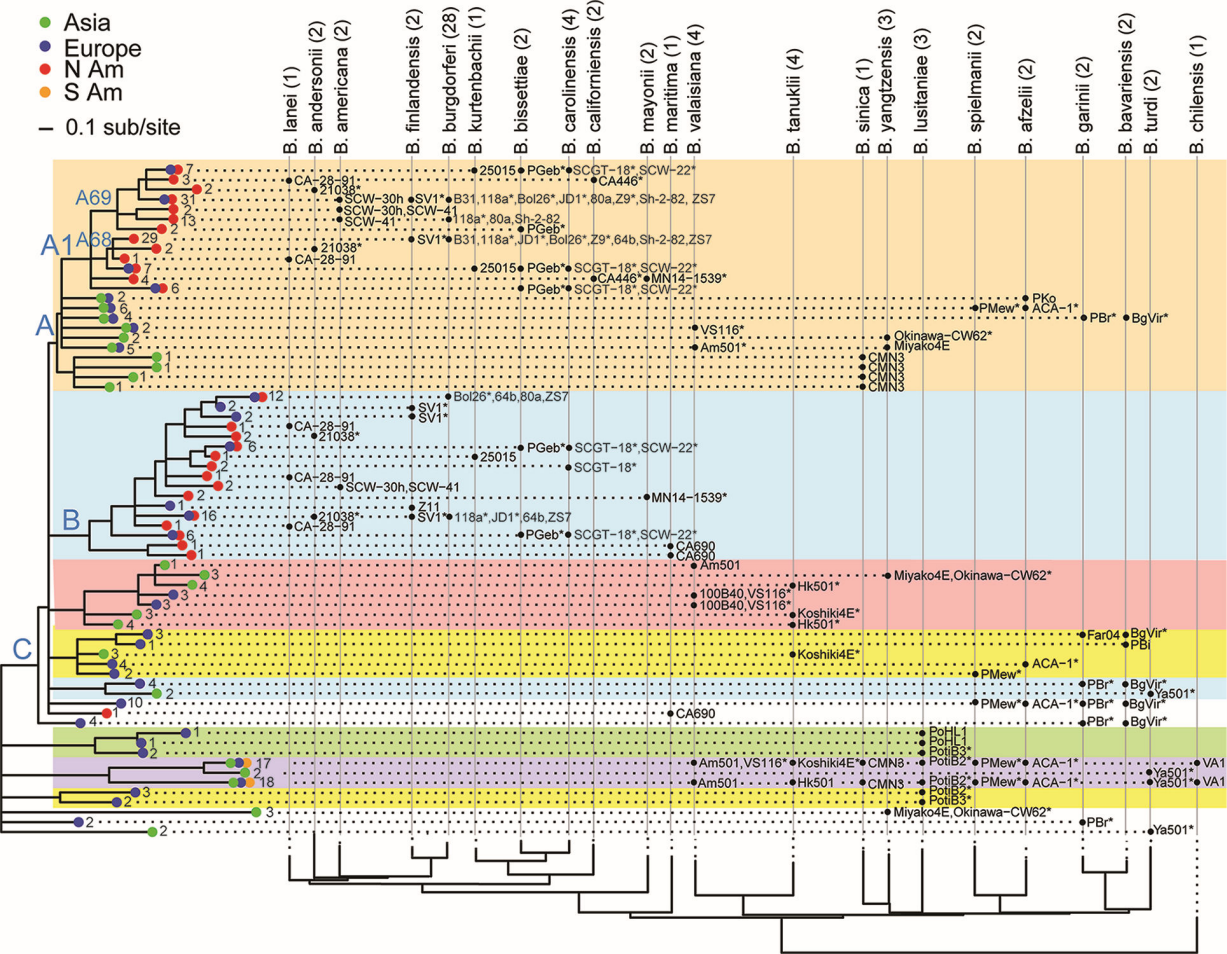
Major PFam54 protein variants from sequenced genomes. The tree of PFam54 “variable” region proteins contains 68 tips representing 324 “variable PFam54” genes found on 75 genomes. The tree is displayed as described in Fig. 7 except for the following: (i) branch tips with ≤0.30 amino-acid differences/site were merged, (ii) blue letters at the left mark clades discussed in the text, (iii) colored boxes highlight major branches with more than one member, (iv) numbers at the branch tips give the number of alleles merged at that tip, and (v) asterisks (*) indicate that there are similar isolates not shown in the figure as follows: 118a*+156a + 29,805 + 72a + 94a + B331 + CA-11.2A + MM1 + N40 + WI91-23; 21038* + MOD-5; ACA-1*+PKo + K78 + Tom3107; Am501* + 100B40 + VS116 + Tom4006; BgVir* + PBi; Bol26* + Bol29 + Fr-93-1 + NE5248 + NE5261 + NE5267 + NIH3 + NIH5 + NIH8; Hk501* + Koshiki4E + TanegashimaAS13 + TanegashimaAS9; JD-1* + SCW-9; Koshiki4E* + TanegashimaAS9; MN-1439* + MN-1440; Okinawa-C62* + Okinawa-C61; PBr* + Far04; PGeb* + DN127; PKo* + Tom3107; PMew* + A14S; PotiB2* + PotiB3 + PoHL1; PotiB3* + PotiB2; SCGT-18* + MI-3; SCW-22* + BULH-2; SV1* = Z11; VS116* + Tom4006; Ya501* + 0473; Z9* + 217_5 (the sequence of the 217_5 PFam54 array is incomplete, but the sequenced portion is very similar to that of Z9).

A total of 324 “variable region PFam54” protein sequences were obtained from 75 genomes and consolidated to 68 allele types (branch tips) in Fig. 9, each of which differs in amino acid sequence by ≥0.30 amino acid differences/site. The treemap contains a well-supported clade A (shaded with an orange background in Fig. 9) with a largely North American “A1” subclade. Cross-continental sharing of variant alleles in this subclade supports the relatively recent migrations of *B. burgdorferi* and *B. finlandensis* from North America to Europe. Within the A1 subclade, two lineages correspond to B31 A68 and A69 orthologous subclades, both of which exist in the genomes of *B. lanei*, *B. andersonii*, *B. finlandensis*, *B. burgdorferi*, *B. kurtenbachii*, *B. bissettiae,* and *B. carolinensis* suggesting the duplication of an ancestral gene to form A68 and A69 occurred in a common ancestor of these species. In the *B. americana* genomes, the A68 ortholog has been lost while the A69 ortholog has expanded. The A69 orthologs have also expanded in a subset of *B. burgdorferi* strains (e.g., 80 a, Sh-2-82, and 118a; see legend to Fig. 9 for full list). No obvious A68 and A69 orthologs are present in the two *B. mayonii* genomes or the two *B. californiensi*s genomes, but, along with *B. bissettiae* and *B. carolinensis,* they carry alleles in other A1 clade lineages suggesting a possible early separation or rapid evolution that makes orthology with A68 or A69 ambiguous. The Clade A PFam54 genes in species with chromosomes of European origin (in the right half of the figure) likely represent a divergence from a common A68 or A69 ancestor. PFam54 clade B homologs (shaded in blue in Fig. 9) represent an expansion within the North American chromosomal lineage, although it contains European *B. burgdorferi*, *B. bissettiae,* and *B. finlandensis* isolates due to dispersals (above).

The PFam54 homologs in the large clade defined by the parts of branch “C” (shaded in pink, yellow, blue, and white) that are outside clades A and B are Eurasia-specific variants except for one homolog in the *B. maritima* CA690 genome. Since the latter genome is the outgroup to all other North American species, this homolog may represent an ancestral lineage rather than a horizontal transfer from Eurasia to North America. Similarly, six PFam54 lineages connected directly to the root node (shaded in green, purple, gold, and white at the bottom of the gene tree) are exclusively Eurasian variants. We note that the PFam54 proteins of *B. lusitaniae* consist of seven variants in three of these lineages. Five variant types exist exclusively in this species, suggesting species-specific duplications and adaptation. Unlike the other 22 species, *B. lusitaniae* is unique in that it commonly uses reptile (lizard) hosts (125), which may have driven its unique OspC and PFam54 divergence.

Fairly recent PFam54 array gene duplications have occurred within several species, for example, *B. sinica* (within the orange clade), *B. tanukii* (within the pink clade), *B. valaisiana* (within orange and pink clades), and *B. lusitaniae* (green and lower yellow clades); in addition, there are a significant number of PFam54 pseudogenes in these arrays suggesting ongoing gene loss. Thus, in contrast to the largely single-copy *ospC*, *dbpA,* and “constant” PFam54 array loci, the variable portion of the PFam54 array contains multiple paralogous genes with duplications and losses having occurred frequently at various levels of species and strain diversification. Unambiguous examples of horizontal transfer between species are less common than duplication and losses, but they appear to have happened in a few cases. Members of a given species usually have arrays that are more similar to one another than those in the other species. For example, there are four closely related types that contain different combinations of five alleles in *B. burgdorferi*. Several of these are most closely related to alleles in *B. finlandensis, B. americana,* and/or *B. andersonii* (Fig. 9). These four types are indicated in Fig. S12, where there are several PFam54 inconsistencies with the overall lp54 SNV tree that suggest transfer of the array relative to the rest of lp54; for example, the lp54 64b/NIH5 branch is very divergent from B31 but has a B31-like PFam54 array, and Sh-2-82 and 156a lp54 plasmids are very divergent overall but have very similar PFam54 arrays.

The PFam54 array locus clearly evolves rapidly through lineage-specific duplications and losses, as well as by the maintenance of a large number of highly divergent variants within the same locus. The lineage-specific distribution of PFam54 variants is consistent with their putative functional roles in neutralizing complement-mediated killing in niche-specific host species, while the co-existence of a large repertoire of PFam54 variants within genomes may be associated with the largely host-generalist nature of these species (115, 116).

## DISCUSSION

Whole-genome sequencing and comparative analysis are essential in setting the groundwork for the identification of the genomic determinants of Bbsl phenotypic variability, including factors contributing to antigenic variations, host and vector preferences, as well as human pathogenicity (27, 31, 65, 82, 126–128). Currently, four Bbsl species are known to be the main cause of Lyme disease in humans worldwide (10, 27, 31). In the USA, *B. burgdorferi* causes the vast majority of human infections (8, 59, 129), with *B. mayonii* causing a few cases of human illness in the upper Midwest region, while in Europe and Asia, the main species causing Lyme disease are *B. afzelii*, *B. bavariensis*, *B. burgdorferi,* and *B. garinii* (128, 130). Bbsl species vary greatly with respect to their clinical manifestations in humans. For example, Lyme arthritis has been associated with infections with *B. burgdorferi,* acrodermatitis chronica atrophicans with *B. afzelii,* and neuroborreliosis with *B. garinii* and *B. bavariensis* (10, 27, 126, 131, 132). Although a specific subset of *B. burgdorferi* strains (e.g., ribosomal sequence type 1 and OspC sequence type A, among others) circulating in North America have been associated with disseminated human Lyme disease (109, 110, 133), and a number of genes required for tick or vertebrate host infection have been identified, the molecular basis of Lyme disease and its symptoms remain poorly understood. Until now, the relationship of the pathogenic species to species that are not known to cause disease in humans has been largely unexplored, as are questions about whether the non-pathogenic species might be a genetic reservoir for the future evolution of the pathogenic Bbsl species.

Similarly, the molecular basis of ecological niche adaptation by Bbsl species, including their genome evolution, the function of many of the plasmid-encoded genes, and the presence of pseudogenes, is poorly understood. For example, it is unclear whether Bbsl species ecology (e.g., association with non-human-biting vectors) may prevent frequent human contact (134). An exception is *B. valaisiana,* which is associated with a generalist vector and its prevalence is regionally comparable with other Bbsl species that cause human disease (e.g., *B. garinii*), but it has never been isolated from a human patient with Lyme disease, thus has been considered non-human pathogenic (135).

To build a firm foundation that will allow these questions to be addressed, we determined whole-genome sequences for 47 geographically diverse Bbsl isolates that include 13 species for which no genome sequence was known and that include all 23 species. Analysis of these genomes confirms that all 23 species have highly syntenic linear chromosomes with covalently closed hairpin telomeres, and all carry universally present and syntenic circular cp26 and linear lp54 and lp17 plasmids (one isolate, *B. americana* SCW-41 has no lp17, but it could have been lost during laboratory propagation). In addition, they carry between 3 and 17 other “variable” plasmids of at least 30 compatibility types that are sporadically present without a strong phylogenetic pattern.

Based on our diverse geographic and phylogenetic sampling, the genomic diversity revealed in the present study corroborates our earlier pan-genome analyses and leads us to conclude that the global biodiversity of the genus Bbsl is defined by an exceptionally tightly closed pan-genome (44, 49); very few new gene families were found after more than doubling the number of species for which genome sequences are available. Our analysis shows a complete lack of horizontally transferred DNA from other bacterial genera, suggesting a co-adapted gene pool reflective of an obligatory association with the tick and host. The different Bbsl species share similar (but not identical) plasmids. Thus, the plasmid types and their gene families are most likely primordial in origin, existing in the most recent common ancestral population predating the division of Pangea, estimated at 55–180 Myr ago.

The new sequence information was used to perform several types of whole-genome phylogenetic reconstructions to examine the history of global diversification within this genus. Removal of genomic sequences exhibiting high rates of intraspecies recombination and interspecies introgression led to strong statistical support for individual phylogenetic (SNV) tree branches (i.e., bootstrap values of 80% or more). This analysis provides a robust, up-to-date, and comprehensive view of within- and between-species diversity in the Bbsl group and allows an evaluation of the history of global diversification and dissemination of this genus; because of the geographic diversity in our samples, we were able to identify a number of past inter-continental lineage dispersal events.

Multispecies coalescent analysis provides additional demographic information, including effective population sizes and the possible timing of species divergence (91, 92). In the absence of bacterial fossil records, bacterial species divergence times are at best very crude guesses since population size, mutation rate, and generations/year could vary across the ages. With these limitations in mind, we estimated divergence time using the separation of the Pangea primordial supercontinent land mass that occurred between 180 and 55 Myr ago (1, 2). The mean age of the last common ancestor of all Bbsl species is estimated to be 117 ± 58 million years (Myr) (see Table S5). Furthermore, the estimated age of the last common ancestor of the Eurasian and North American clades in the MCC tree is 101 ± 50 Myr, consistent with the time of the opening of the North Atlantic Ocean, the last phase of the Pangea breakage, during the early Cenozoic era 66–23 Myr ago. Alternatively, we estimated divergence time based on an assumption of ~10^–12^ nucleotide substitutions per base pair per generation in Bbsl species (see METHODS). Although it is very uncertain, this divergence dating is consistent with the idea that ancestral Bbsl species were widely dispersed as early as 93 ± 87 Myr ago, a point in time during or predating the division of the Pangea supercontinent. A likely error in this calculation is an overestimation of the total number of generations; fewer generations/yr would push these dates even farther back in time. Table S5A lists the estimated divergence times for 23 Bbsl species. The ortholog-based species tree and the SNV-based chromosomal and plasmid phylogenies are quite consistent with one another and provide strong support for the division into two geographically associated clades, one of Eurasian origin and another of North American origin (Fig. 4 and 5) as previously reported and based on MLST data (56, 82). The estimated effective population sizes of these Bbsl species appear to have been relatively stable over time with no apparent demographic events, such as population bottlenecks or founder effects (Table S5A). European *B. bavariensis* isolates are, however, less diverse than Asian isolates, suggesting founder effects of its European populations (99, 100).

By removing deleterious mutations and generating novel adaptive genotypes, recombination is a powerful accelerating force for species adaptation (83, 136). Although *Borrelia* populations are predominantly clonal, our analysis of *B. burgdorferi* populations confirmed within-species recombination as a major contributor to the existing genomic diversity. Recombination facilitates adaptation, and without it, a population would be unable to sever the genetic linkages between deleterious and beneficial mutations (i.e., the Hill-Robinson Effect); this would eventually lead to loss of beneficial variations due to the inevitable accumulation of deleterious mutations elsewhere (i.e., Muller’s Ratchet) (136–138). We detected hotspots in *B. burgdorferi* genome regions that have been exchanged between species in the North American clade. These include cp26 *ospC* and lp54 *dbpAB,* which are hotspot loci, although recombination occurs at housekeeping loci throughout the main chromosome (Fig. 3). In addition, analysis of the “core plasmids” identified whole plasmids and plasmid loci that have been exchanged among the Bbsl species and lineages within species. In sum, the preferential maintenance of recombinant variants at these lipoprotein loci underscores the substantial fitness advantage for genetic variability at host-interacting loci, while recombinant variants at housekeeping loci are largely neutral.

We also examined the evolutionary history of three host-interacting, highly variable lipoproteins, OspC, DbpA, and the PFam54 proteins. Strikingly, our findings show that with one exception, the lack of PFam54 variable genes in *B. japonica*, these genes are universally conserved across all 23 species in the Bbsl genus, so they appear to be important and primordial conserved aspects of the Bbsl life cycle. At the *dbpA*, *ospC,* and PFam54 loci, there are relatively few unambiguous gene- and species-tree inconsistencies that would suggest horizontal gene exchange between species, consistent with the findings that recombination occurs more frequently within species than between species where Bbsl species coexist (139, 140).

### Limitations and future directions

The current study is, of course, not without limitations. First, the choice of strains for whole-genome sequencing and subsequent analysis was by design, not random; however, random strain choice is required for a perfectly unbiased population genetics analysis. Maximizing phylogenetic and geographic coverage, as we did, allows observation of a more nearly full extent of diversity, but it is likely to inflate the genetic diversity and recombination measures in *B. burgdorferi* in the presented analysis (Fig. 3). These biases are partially mitigated by the extraordinarily even distribution of different strains with different OspC types within *B. burgdorferi* populations (59). Second, the strains selected for whole-genome sequencing are not primary host or tick isolates but have gone through various levels of laboratory passage in culture (Table S1A). Genetic alternations such as plasmid loss may have occurred during culture in artificial media (141). Indeed, in the *B. valaisiana* 100B40 genome that we sequenced twice from different DNA preparations, plasmid lp25 plasmid was only present in one of the two sequences. On the other hand, plasmid and chromosome sequences *per se* appear to be quite stable in culture. Our experiences and those of other laboratories have shown that differences and rearrangements (ignoring changes in the *vls/vlsE* expression cassette) between independent cultures of the same isolate are uncommon. For example, (i) when the *B. burgdorferi* B31 genome or parts thereof have been resequenced from independent cultures, differences from the originally reported sequence are rare and (ii) in a specific current example, in our two independent 100B40 sequences determined by current PacBio methodology the 1,216,726 bp genome (without lp25 above) has only 30 differences scattered across the chromosome and plasmids. All of these are single bp differences, and all but four are possible sequencing errors in the lengths of long (4–10 bp) mononucleotide runs. Third, the non-core plasmids of the 47 genomes studied here were largely not included in this analysis. These sequences have been submitted to the public database but are still under annotation, so we have not yet performed, for example, an updated genome-wide pan-genome gene content analysis, which would identify strain- and species-specific gains and losses of genes (142). This also means that some universally present genes contributing to vertebrate host and tick invasion and persistence are not included in the present study. Notably, the within-species sequence diversity of the multi-copy *vls* cassettes rivals that of *ospC* (103, 143). Evolutionary analysis of the *vls* systems in these genomes is ongoing, including within- and between-species sequence diversity and the history of plasmid translocation. More comprehensive analyses of the non-core plasmids will be the subject of subsequent publications.

The new sequence information and analyses reported here provide a substantial and robust foundation for future research in the Lyme disease arena. In particular, the findings of recombination hotspots at host-interaction loci suggest that these regions are the primary candidates for identifying genomic variability associated with human virulence, tissue invasiveness, and differential clinical manifestations of the Lyme disease pathogens (127). A full understanding of introgression and recombination across this genus will require still more genome sequences from species other than *B. burgdorferi*, and the adaptive nature and functional consequences of core and non-core plasmid variability remain to be characterized in more detail by comparative and experimental studies. Ecologically and functionally important sequence variations may be distinguished by determining the rates of synonymous (*dS*) and non-synonymous (*dN*) substitution as a means to screen for genes evolving in response to positive, negative, and neutral constraints across the whole Bbsl genome, including non-core plasmids (55, 144–146).

## MATERIALS AND METHODS

### Strain selection and growth

Forty-seven Bbsl isolates were selected for whole-genome sequencing to maximize within- and between-species phylogenetic diversity coverage. One to five strains were chosen for each unsequenced Bbsl species according to an MLST (multi-locus sequence typing) analysis (25) and strain availability. Multiple strains from each species enable analysis of within-species genomic diversity. We also selected *B. burgdorferi* isolates from each of the unsequenced OspC-delineated clonal groups that were available (57, 67). The isolates were cultivated in BSK medium. Culture passage information is given in Table S1A and was less than 10 passages in culture in all cases. Our standard passage protocol is a 1 to 10 dilution from the previous culture. We point out that since Bbsl isolates are known to lose plasmids in culture, all of the genomes studied here could have lost plasmids between stain isolation and DNA preparation. Because of complexities in shipping live cultures across international borders, all non-North American isolate DNAs were purified in the country of origin and shipped to our sequencing facilities (see below). North American isolates were propagated and DNA was prepared in the laboratory of B. Luft. The isolates whose genomes were sequenced are listed in Table S1A.

### Whole-genome sequencing and assembly

Thirty-six of the genomes described here were sequenced with Pacific Biosystems technology at the Institute for Genome Sciences at the University of Maryland Medical Center (Baltimore, MD). One of these genomes, that of *B. valaisiana* 100B40, was sequenced independently a second time at the German National Reference Centre for *Borrelia*. Genomic DNA was isolated using the Qiagen DNeasy system as recommended by the manufacturer, and 10–60 Kbp libraries were prepared with an SMRTbell Express Template Prep Kit 2.0 and sequenced on the PacBio platform using P6-C4 chemistry, thus generating ~500 Mbp of sequencing reads per Bbsl isolate. Celera Assembler PBcR, MHAP, and the Hierarchical Genome Assembly Process (HGAP) developed by PacBio were used for the assembly of high-quality genomes using only PacBio reads (147, 148). HGAP used shorter PacBio reads to generate multiple alignments for error correction of longer reads. The resulting corrected long reads were assembled using the Celera Assembler, and the consensus sequence was polished using the PacBio Quiver module, an HMM-based algorithm for calling highly accurate consensus from multiple alignments of PacBio reads. Similarly, the Celera Assembler PBcR and MHAP used shorter PacBio reads for error correction followed by traditional overlap-layout-consensus assembly. The results and metrics from each set of assemblies for each isolate were compiled into a summary table for evaluation and comparison. The assemblies were evaluated using the QUAST and Hawkeye packages (149, 150), and optimal assemblies were selected based on a combination of metrics, including but not limited to contig count, contig N50, genome size, and predicted mis-assembly rate. Since complete reference genomes (strain B31) for Bbsl were available, we used QUAST to identify and classify the potential mis-assemblies (e.g., relocation, translocation, and inversion events) using a comparative alignment analysis. Potential mis-assembly events were manually reviewed to select the best assembly data set for each isolate for downstream analysis and data submission.

Eleven of the genomes presented here, those of isolates 21038, BUL-H-2, CA446, CMN3, MI-3, MOD-5, M7p, PoHL1, PotiB3, SCW-22, and SCW-30h, were sequenced at New England Biolabs (Ipswich, MA) using the Pacific Biosciences Sequel II system as described in Rudenko et al. (123). Barcoded random libraries were prepared using the PacBio SMRTbell express template kit 2.0 according to the manufacturer’s instructions. Sequencing was performed using one Sequel II cell for 4 to 6 barcoded genomes which resulted in a range from 1.4 to 76 Gb total sequence yielding 140 to 3,507 Mb high fidelity (HiFi) reads with a mean length of 5,700 to 8,500 bp and median quality values of QV37 to QV45. Genome assembly was performed with the “Microbial Assembly” tool in PacBio SMRTLink 10.2 using 60 Mb of the HiFi reads (30X downsample with 2 Mb expected genome size) with QV >30. The assembly was polished by the PacBio Arrow algorithm, and the telomere ends were examined manually in multiple individual HiFi reads.

The resulting sequences were manually curated as follows: When present, terminal “wraparound” inverted repeat sequences, generated by sequence reads that progress around the ends of the covalently closed hairpin telomeres of linear replicons, were trimmed from the ends of the covalently closed linear replicons; such wraparound sequences were typically between 5 and 15 kbp in length. Direct terminal repeats were merged to circularize contigs where appropriate, and clearly overlapping contigs were merged in a few cases. Linear replicons have a terminal telomere consensus sequence (tip-N_14_TAGTATA-3′; to be described in detail in a subsequent report; S. Casjens and S. Hepner, unpublished). In some cases, sequences from the older PacBio P6-C4 chemistry and HGAP assembly technology did not extend to the telomere of linear replicons and gave somewhat ambiguous assemblies of the family of closely related cp32 plasmids. Thus, in the latter genomes, some submitted cp32 sequences are incomplete, and the accuracy of some cp32 assemblies is suspected to be poor in some regions with relatively few long open reading frames. The P6-C4 chemistry method also resulted in some “orphan” assemblies of incomplete cp32-like sequences that carry no PFam32 gene and appear to have poor ORF quality; these were ignored.

Following chromosome and plasmid assembly and manual curation, genome annotation was performed using the NCBI prokaryotic genome annotation pipeline (151) during the genome submission process through the NCBI genome submission portal (https://submit.ncbi.nlm.nih.gov).

### Sequence analysis

#### SNV identification

SNVs (single-nucleotide variants) and indels in each genome with respect to the reference B31 chromosome, cp26, lp54, and lp17; AE000783, cp26 AE000792, lp54 AE000790, and lp17 AE000793, respectively, were identified using the program BWA (76). A custom BASH script was used to produce a variant call format (VCF) file per replicon based on BCFTools, VCFTools, and BpWrapper (121, 152). While indels can be useful phylogenetic markers (17), to simplify bioinformatics workflow we used only SNVs for the subsequent phylogenetic and recombination analysis.

#### Within species recombination

Intra-specific recombination analysis was performed using the 28 *B. burgdorferi* genome sequences using the bi-allelic SNVs on the main chromosome, cp26, lp54, and lp17. The focus on intra-specific sequences is based on the consideration that recombination occurs predominantly among the coexisting and con-specific strains within natural populations. Technically, the intra-specific sequences are minimally divergent (with 0.40% average sequence differences on the main chromosome) and thus less subject to false-positive results in the recombination analysis due to recurrent nucleotide substitutions at the same sites. At hypervariable loci, the high sequence variability maintained by diversifying selection is understood as a result of recombination, not elevated mutation rates (57, 79, 153). The following tests quantify recombination by identifying phylogenetic inconsistencies (or homoplasies) either at pairs of SNV sites (LDhat) or on consecutive SNVs (ClonalFrameML and ABBA-BABA tests).

Intra-species recombination rates for all pairs of bi-allelic SNPs were estimated using LDhat (77). The haplotype sequence alignment file was converted to LDhat phased data using the program “convert,” and the LDhat program “interval” was used to estimate the posterior mean recombination rate per site (*ρ = 2* Nr, where *N* is the effective population size and *r* is the recombination per site) with a setting of 10 million iterations, a gap penalty of 10 and sampling in every 5,000 iterations. Existing lookup tables from the LDhat package were used to perform the analysis. The first 10% of the iterations were dropped as burn-in, and the results were summarized by the LDhat program “stat.” The results were plotted in the R statistical computing environment to show sites with high recombination rates and recombination hotspots.

As an independent approach, we identified recombination tracks and estimated recombination relative to mutation rates (*ρ/θ*) using ClonalFrameML (v1.12) (81). ClonalFrameML runs were performed with the transition to transversion ratio of *κ* = 4 and 100 rounds of simulation to obtain parameter uncertainty. Recombination rates were estimated separately for the four core replicons (main chromosome, lp54, cp26, and lp17) using respective intra-specific replicon alignments and a maximum likelihood tree based on the SNVs on the main chromosome (see below). The chromosome, lp54, and cp26 alignments were reconstituted from the VCF files using the “consensus” program of the BCFTools with the corresponding B31 replicon as the reference sequence. The lp17 alignment was based on the ~12 kb syntenic region encompassing orthologs of *BB_D01* through *BB_D24* and aligned with MAFFT (154). Results of ClonalFrameML, including recombination tracks and a phylogenetic tree with branch lengths re-estimated by excluding recombination, were visualized with a custom R script.

#### Between-species introgression

Inter-species genetic exchange was analyzed with ABBA-BABA statistics (also known as the *D* statistics). This test detects introgression based on clusters of homoplasies (i.e., phylogenetically inconsistent SNVs) in a genomic region with respect to a species phylogeny consisting of three ingroup genomes (P1, P2, & P3) and an outgroup (O) genome [((P1, P2), P3), O] (85). To quantify introgression between species, we performed such scans of the chromosome, cp26, lp54, and lp17. Using Dfoil, four-taxon *D*-statistics were calculated by scanning the genome alignments in a locus-by-locus fashion with the following settings: one of the 20 North American *B. burgdorferi* strains as P1, sister species *B. finlandensis* strain SV1 as P2, one of nine other North American species as P3, and *B. chilensis* strain VA1 as outgroup O (84). Dfoil assesses the significance of a *D*-statistic by comparing it to the expectation under the assumption of random lineage sorting and performing a χ^2^ goodness-of-fit test. Genome-wide average *D*-statistic values for the chromosome, plasmid cp26, lp17, and lp54 were estimated from the combined summary statistics of all loci. Selected putative introgressed loci inferred by the *D*-statistics analysis were validated by comparing the sequence similarities between potential donor and recipient sequences.

### Phylogenetic reconstruction

#### SNV trees

For phylogenetic SNV tree analyses, regions with a nonhomologous sequence as well as high recombination and introgression rates (≥2 standard deviations from the mean rates) were removed from the above SNV alignments with BCFTools and VCFTools (152). Phylogenetic trees were inferred from these recombination-depleted SNV alignments using IQtree (version 2.1.2) with the best-fit model, ascertainment bias correction, and 1,000 rapid bootstrap replicates for each replicon (87). Each tree was rooted using *B. chilensis* VA1, a South American strain. Visualization of the inferred trees was done by the R software package *ggtree* (155).

#### Species tree and divergence times

To infer a Bbsl species tree, a data set consisting of 304 single-copy orthologous genes from the main chromosome of the 78 isolates in Table S1A and B was prepared. Gene orthology was determined based on a combination of BLAST (156) search, clustering by CD-HIT (157), and gene synteny as described previously (44). Orthologous genes that contain recombination hotspots or signatures of introgressions were excluded. The program BPP was used to build the tree; it is a Bayesian Markov chain Monte Carlo (MCMC) program, which uses DNA sequence alignments from multiple loci and multiple closely related species under the multispecies coalescent (MSC) model (93). Species phylogeny estimated by a multi-species coalescent model allows individual genes to have stochastic coalescent times following a single underlining species tree. To run BPP, a mapping file was prepared to designate the 78 isolates from 23 Bbsl species.

Briefly, we ran A11 (joint species delimitation and species tree inference) and A01 (species tree inference) analyses in BPP to infer the best species tree with species delimitation using the following parameters: inverse gamma prior with *α* = 3 and *β* = 0.015 for theta (*θ*), inverse gamma prior with *α* = 3 and *β* = 0.25 for tau (*τ*), fixed locus rate and local clock model where mutation rates change over branches independently among loci. The prior value of theta (*θ_0_*) was chosen based on the average sequence identity of ~0.5%. The prior tau value (*τ_0_*) was chosen based on the ~10% sequence divergence between the *B. chilensis* and other Bbsl species. BPP analyses were performed on DNA alignments of 304 orthologous genes. We ran two chains of 200,000 iterations with sampling in every two iterations. The first 10% of samples (20,000 iterations) were discarded as burn-in.

Once the species delimitation and species tree were inferred from A11 and A01 analyses, an A00 analysis was run with the A01-inferred species tree to estimate the parameters of species divergence times and population sizes. Likewise, A00 analysis was performed on the same data set with 200,000 iterations with sampling in every two iterations and 10% burn-in. Tracer v.1.7.1 was used to assess the convergence and to confirm the effective sample sizes were greater than 200 for each parameter (158). Species divergence times and effective population sizes were estimated using “*bppr,*” a helper R package for BPP (159). The species divergence time was estimated first based on a mutation rate of *m* = 1×10^−12^ substitutions per site per generation. This rate was based on the estimation of 10^−9^ substitutions per year and an average generation time of 7 to 20 h, or ~650 generations per year (51, 95, 96). Separately, the divergence time was estimated based on the age of 180–55 million years ago for the continental separation associated with the divergence of the Eurasian and North American clades. The effective population sizes were estimated based only on the same substitution rate.

### Biogeographic reconstruction and synteny analysis

BioGeoBears was used to infer the global geographic dispersal of species by reconstructing their ancestral geographic distributions (94). Geographic origins were recorded based on the contemporary distribution of species from four geographic locations: Asia, Europe, North America, and South America. To find the best fitting model, comparisons between different ancestral state reconstruction models were performed using BioGeoBears, and ancestral biogeographic states were inferred using the best model. Reconstructed global geography was visualized on the inferred BPP maximum clade credibility (MCC) tree.

Following the identification of orthologous gene groups in Bbsl core and non-core plasmids, we performed an analysis of gene gains and losses on the following Bbsl on the cp26, lp54, and lp17 plasmids. To update the results of earlier studies (55, 97), we expanded our analysis to 78 strains from 23 Bbsl species. Evidence for gene duplications, lineage-specific gene losses, and translocations were identified by genome synteny analysis using genoPlotR according to the chromosomal tree (160).

### Molecular evolution of lipoprotein gene families

Protein sequences homologous to OspC (BB_B19), DbpA (BB_A24), and CspA (BB_A68 and BB_A69) in the B31 genome were extracted from the annotated Genbank using BpWrapper (121). OspC and DbpA are both single-locus protein families, except for the 2 to 3 copies of *ospC* in each of the three *B. lusitaniae* genomes. The CspA homologs included only the variable portion of the PFam54 gene array on the lp54 plasmid and excluded the universally present PFam54 loci orthologous to BB_A64, BB_A65, BB_A66, and BB_A73 (Fig. 6).

For each gene family, the protein sequences were aligned with MUSCLE (161), and FastTree (162) was used to build a maximum likelihood tree rooted at the midpoint with the *biotree* utility of BpWrapper (121). To consolidate gene-tree tips sharing a high sequence identity (e.g., a threshold of ≤*d* amino-acid substitutions per site), a tip-trimming algorithm was developed and implemented in the *biotree* utility (with the “--trim-tips” option). Starting from the root and for each internal node, distances to all its descendant tips are calculated. If a distance to any descendant exceeds the threshold value (>*d*), the node is retained. Otherwise, the internal node and all its descendants are removed except for a randomly selected descendant tip. The operation is recursively applied to every internal node. The trimmed gene tree is further consolidated by removing branches with low (e.g., <0.9) bootstrap support. Effectively, this tip-trimming algorithm greatly reduces the number of tree tips sharing high sequence identity while retaining the overall tree topology and distances, highlighting major protein variants on a gene tree.

Custom R scripts based on *ggtree* (155) followed by manual editing with Adobe Illustrator were used for visualization of the consolidated gene trees while grouping variants based on species. The resulting visualization, called “treemap” here, allows co-visualization of the gene and species trees, facilitating visual analysis of strain migrations between continents and gene exchanges between species.

## Data Availability

The Bbsl genome sequencing project is archived in the NCBI database under BioProject PRJNA431102, and Biosample accessions are listed in Table S1. Raw sequencing reads are available in the NCBI SRA database. Fully assembled and annotated core genome sequences are available in the NCBI nucleotide database. Comparative genomic sequences and online tools for data retrieval and analysis are available at a companion website, BorreliaBase: https://borreliabase.org/. Alignments, trees, and computer codes are available in the Github repository at https://github.com/weigangq/Bbsl_2023, including the 304 nucleotide sequence alignments of chromosomal orthologs used for reconstructing the species tree.

## SUPPLEMENTAL MATERIAL

Supplemental material mbio.01749-24-s0001.pdf Tables S1 to S5; Figures S1 to S15.

ASM does not own the copyrights to Supplemental Material that may be linked to, or accessed through, an article. The authors have granted ASM a non-exclusive, world-wide license to publish the Supplemental Material files. Please contact the corresponding author directly for reuse.
