# Supplementary material for: Natural selection and recombination at host-interacting lipoprotein loci drive genome diversification of Lyme disease and related bacteria
Source: mBio. 2024 Aug 15;15(9):e01749-24. doi: 10.1128/mbio.01749-24 (PMC11389397; doi:10.1128/mbio.01749-24)
Supplement: Supplemental material — Tables S1 to S5; Figures S1 to S15. [file mbio.01749-24-s0001.pdf]

Table of contents

Table S1A	Page 2	Isolates whose genome sequences were determined in this study
Table S1B	Page 3	Other genomes used in this study
Table S2A	Page 4	Plasmids sequenced in this report (part 1)
Table S2B	Page 5	Plasmids sequenced in this report (part 2)
Table S3	Page 7	Loci with high recombination rates in the genus *Borreliella*
Table S4	Page 9	Introgressed loci analysis with four-taxon *D*-statistics
Table S5A	Page 10	Divergence times and ancestral population sizes
Table S5B	Page 11	Biogeographic models tested using BioGeoBears
Figure S1A	Page 12	*Borreliella* chromosome terminal extensions - North America
Figure S1B	Page 14	*Borreliella* chromosome terminal extensions - Eurasia
Figure S2	Page 15	*Borreliella* plasmid PFam32 protein neighbor-joining tree
Figure S3	Page 17	*B. californiensis* CA446 linear plasmid lp5
Figure S4A	Page 18	Recombination rates and hotspots on the *B. burgdorferi* chromosome and plasmids cp26, lp17 and lp54
Figure S4B	Page 20	Cross-species exchange (introgression) between *B. burgdorferi* and *B americana*
Figure S5	Page 22	Phylogeny of the circular plasmid cp26
Figure S6	Page 24	Phylogeny of the linear plasmid lp54
Figure S7	Page 26	Phylogeny of the linear plasmid lp17
Figure S8	Page 28	Loss of *guaAB* genes from cp26 in *B. sinica* and *B. andersonii*
Figure S9	Page 29	Comparison of chromosome and cp26 trees
Figure S10	Page 30	*Borreliella* lp54 terminal differences
Figure S11	Page 31	*B. japonica* lp54 structure
Figure S12	Page 33	Comparison of chromosome and lp54 trees
Figure S13	Page 34	Organizational subtypes of *B. burgdorferi* lp17's
Figure S14	Page 35	Comparison of chromosome and lp17 trees
Figure S15	Page 36	lp17 left end extensions
References	Page 37	

Table S1A. Isolates whose genome sequences were determined in this study

Isolate (T = species type strain)	Species (OspC type)	Isolation/ publication year	Isolation location	Isolation host	Biosample number (all in BioProject PRJNA431102)	Number of passages in culture	Strain isolation reference
SCW-30h	*B. americana*	1995	South Carolina, USA	*Ixodes minor*	SAMN10141353	<P5	Rudennko et al. (2014)
SCW-41(T)	*B. americana*	1995	South Carolina, USA	*Ixodes minor*	SAMN10141354	P3*	Rudenko et al. (2009b)
21038(T)	*B. andersonii*	1988	New York, USA	*Ixodes dentatus*	SAMN34060371	<P5	Anderson et al. (1988)
MOD-5	*B. andersonii*	1998	Missouri, USA	*Ixodes dentatus*	SAMN34060372	P5	Oliver et al. (1998)
PGeb	*B. bissettiae*	1996	Germany	*Human*	SAMN10141355	P6	Margos et al. (2016)
217_5	*B. burgdorferi* (L)	2008	Poland	*Ixodes ricinus*	SAMN10141360	P2	Qiu et al. (2008)
80a	*B. burgdorferi* (N)	2004	New York, USA	*Human*	SAMN10141361	P3	Qiu et al. (2004)
Bol29	*B. burgdorferi* (S)	2008	Italy	*Human*	SAMN10141362	P2	Qiu et al. (2008)
Fr-93-1	*B. burgdorferi* (Q)	2007	Poland	*Ixodes ricinus*	SAMN10141363	P2	Qiu et al. (2008)
NE5248	*B. burgdorferi* (S)	2010	Switzerland	*Ixodes ricinus*	SAMN10141364	<P5	Rudenko et al. (2013)
NE5261	*B. burgdorferi* (V)	2010	Switzerland	*Ixodes ricinus*	SAMN10141365	<P5	Rudenko et al. (2013)
NE5267	*B. burgdorferi* (V)	2010	Switzerland	*Ixodes ricinus*	SAMN10141366	<P5	Rudenko et al. (2013)
NIH3	*B. burgdorferi* (A)	2008	Virginia, USA	*Human*	SAMN10141357	P3	This report
NIH5	*B. burgdorferi* (A)	2008	Virginia, USA	*Human*	SAMN10141358	P3	This report
NIH8	*B. burgdorferi* (A)	2014	Maryland, USA	*Human*	SAMN10141359	P3	This report
SCW-9	*B. burgdorferi* (L)	1994	South Carolina, USA	*Sigmodon hispidus*	SAMN10141367	<P5	This report
Sh-2-82	*B. burgdorferi* (K)	1982	New York, USA	*Ixodes scapularis*	SAMN10141368	P4	Schwan et al. (1988)
Z9	*B. burgdorferi* (S)	1996	Austria	*Ixodes ricinus*	SAMN10141369	P2	Pierer et al. (1996)
CA443	*B. californiensis*	1995	California, USA	*Dipodomys californicus*	SAMN10141370	P6	Postic et al. (2007)
CA446(T)	*B. californiensis*	1995	California, USA	*Dipodomys californicus*	SAMN34060370	P6	Postic et al. (2007)
BUL-H-2	*B. carolinensis*	1999	Georgia, USA	*Neotoma floridana*	SAMN35100056	P2	This report
MI-3	*B. carolinensis*	1992	Florida, USA	*Sigmodon hispidus*	SAMN34060374	<P5	Oliver et al. (1995)
M7p	*B. carolinensis*	2013	Florida, USA	*Human*	SAMN10141356	<P5	Golovchenko et al. (2016)
SCGT-18	*B. carolinensis*	1996	South Carolina, USA	*Neotoma floridana*	SAMN10141371	<P5	Rudenko et al. (2009a)
SCW-22(T)	*B. carolinensis*	1994	South Carolina, USA	*Ixodes minor*	SAMN34060367	<P5	Rudenko et al. (2009a)
Z11	*B. finlandensis*	1996	Austria	*Ixodes ricinus*	SAMN10141372	P2	Pierer et al. (1996)
HO14(T)	*B. japonica*	1993	Hokkaido, Japan	*Ixodes ovatus*	SAMN10141373	P4*	Kawabata et al. (1993)
Miyazaki2E	*B. japonica*	2008	Miyazaki, Japan	*Apodemus speciosus*	SAMN10141374	P1	This report
25015(T)	*B. kurtenbachii*	1987	New York, USA	*Ixodes scapularis*	SAMN10141375	P4*	Anderson et al. (1990)
CA-28-91(T)	*B. lanei*	1991	California, USA	*Ixodes pacificus*	SAMN10141376	P5	Schwan et al. (1993)
PoHL1	*B. lusitaniae*	2004	Portugal	*Human*	SAMN34060369	P4*	Collares-Pereira et al. (2004)
PotiB2(T)	*B. lusitaniae*	1993	Portugal	*Ixodes ricinus*	SAMN10141377	P5*	Nuncio et al. (1993)
PotiB3	*B. lusitaniae*	1993	Portugal	*Ixodes ricinus*	SAMN34060368	P7*	Nuncio et al. (1993)
CA690(T)	*B. maritima*	2012	California, USA	*Ixodes spinipalpis*	SAMN10141390	P3*	Fedorova et al. (2014)
CMN3(T)	*B. sinica*	1997	Sichuan, China	*Niviventer confucianus*	SAMN34060373	?	Masuzawa et al. (2001)
PMew	*B. spielmanii*	1987	Germany	*Human*	SAMN10141378	P3	Fingerle et al. (2008)
Hk501(T)	*B. tanukii*	1993	Hokkaido, Japan	*Apodemus speciosus*	SAMN10141379	?	Fukunaga et al. (1996)
Koshiki4E	*B. tanukii*	2008	Kagoshima, Japan	*Apodemus speciosus*	SAMN10141380	P2	This report
TanegashimaAS9	*B. tanukii*	2008	Kagoshima, Japan	*Apodemus speciosus*	SAMN10141381	P1	Gokuden et al. (2006)
TanegashimaAS13	*B. tanukii*	2008	Kagoshima, Japan	*Apodemus speciosus*	SAMN10141382	P1	Gokuden et al. (2006)
047-3	*B. turdi*	2005	Kagoshima, Japan	*Ixodes turdus*	SAMN10141383	P3	Gokuden et al. (2006)
Ya501(T)	*B. turdi*	1996	Yamagata, Japan	*Ixodes turdus*	SAMN10141384	?	Fukunaga et al. (1996)
100B40†	*B. valaisiana*	?	Germany	*Ixodes ricinus*	SAMN10141385†	P6*	Becker et al. (2016)
Am501	*B. valaisiana*	1993	Aomori, Japan	*Ixodes columnae*	SAMN10141386	?	Nakao and Miyamoto (1993)
Miyako4E	*B. yangtzensis*	2008	Okinawa, Japan	*Rattus norvegicus*	SAMN10141389	P2	This report
OkinawaCW61	*B. yangtzensis*	2015	Okinawa, Japan	*Crocidura watasei*	SAMN10141387	P5	Margos et al. (2015)
OkinawaCW62(T)	*B. yangtzensis*	2015	Okinawa, Japan	*Crocidura watasei*	SAMN10141388	P4*	Margos et al. (2015)

† The 100B40 whole genome was sequenced twice and is also reported as BioSample SAMN05328503 of BioProject PRJNA327303
* Not incuding a small number of passages during isolation in the laboratories that isolated them
? Not known

Table S1B. Other genomes used in this study

Isolate name (T = species type strain)	*Borrelia* species	Isolation/ publication year	Isolation location	Isolation host	Accession number (Biosample number)	Genome sequence reference
ACA-1	*B. afzelii*	1984	Sweden	*Human*	SAMN02436235	Casjens et al. (2011b)
K78	*B. afzelii*	2014	Austria	*Human*	SAMN03024677	Schuler et al (2015)
PKo	*B. afzelii*	1986	Germany	*Human*	SAMN02604205	Casjens et al. (2011b) ; Glockner et al.
Tom3107	*B. afzelii*	2014	Siberia, Russia	*Ixodes persulcatus*	SAMN02378865	Kurilshikov et al. (2014)
BgVir	*B. bavariensis*	2012	Siberia, Russia	*Ixodes persulcatus*	SAMN02603389	Brenner et al. (2012)
PBi(T)	*B. bavariensis*	1993	Germany	*Human*	SAMN08918524	Glöckner et al. (2004);Margos et al. (2018)
DN127(T)	*B. bissettiae*	1990	California, USA	*Ixodes pacificus*	SAMN02604204	Schutzer et al. (2012)
29805	*B. burgdorferi*	1993	Connecticut, USA	*Ixodes scapularis*	SAMN02436321	Schutzer et al. (2011)
118a	*B. burgdorferi*	2004	New York, USA	*Human*	SAMN02436228	Schutzer et al. (2011)
156a	*B. burgdorferi*	2004	New York, USA	*Human*	SAMN02436325	Schutzer et al. (2011)
64b	*B. burgdorferi*	2004	New York, USA	*Human*	SAMN02436287	Schutzer et al. (2011)
72a	*B. burgdorferi*	2004	New York, USA	*Human*	SAMN02436171	Schutzer et al. (2011)
94a	*B. burgdorferi*	2004	New York, USA	*Human*	SAMN02436324	Schutzer et al. (2011)
B31(T)	*B. burgdorferi*	1982	New York, USA	*Ixodes scapularis*	SAMN02603966	Fraser et al. (1997); Casjens et al. (2000)
B331	*B. burgdorferi*	1997	New York, USA	*Human*	SAMN05763278	Unpublished
Bol26	*B. burgdorferi*	2008	Italy	*Ixodes ricinus*	SAMN02436166	Schutzer et al. (2011)
CA-11.2A	*B. burgdorferi*	1993	California, USA	*Ixodes pacificus*	SAMN02436176	Schutzer et al. (2011)
JD1	*B. burgdorferi*	1987	Massachusetts, USA	*Ixodes scapularis*	SAMN02604203;	Schutzer et al. (2011)
MM1	*B. burgdorferi*	1985	Minnesota, USA	*Peromyscus leucopus*	SAMN07452586	Jabbari et al. (2018)
N40	*B. burgdorferi*	1988	New York, USA	*Ixodes scapularis*	SAMN02604202	Schutzer et al. (2011)
WI91-23	*B. burgdorferi*	1993	Wisconsin, USA	*Melospiza melodia*	SAMN02436285	Schutzer et al. (2011)
ZS7	*B. burgdorferi*	1991	Germany	*Ixodes ricinus*	SAMN02603438	Schutzer et al. (2011)
VA1(T)	*B. chilensis*	2013	Chile	*Ixodes stilesi*	SAMN03166165	Huang et al. (2015)
SV1	*B. finlandensis*	2008	Finland	*Ixodes ricinus*	SAMN02436286	Casjens et al. (2011a)
Far04	*B. garinii*	1999	Denmark	*Fratercula*	SAMN02436278	Casjens et al. (2011b)
PBr	*B. garinii*	1986	Denmark	*Human*	SAMN02436232	Casjens et al. (2011b)
MN14-1420	*B. mayonii*	2016	Minnesota, USA	*Human*	SAMN04979181	Kingry et al. (2016)
MN14-1539	*B. mayonii*	2016	Minnesota, USA	*Human*	SAMN04979182	Kingry et al. (2016)
A14S	*B. spielmanii*	1999	The Netherlands	*Ixodes ricinus*	SAMN02436233	Schutzer et al. (2012)
Tom4006	*B. valaisiana*	2014	Siberia, Russia	*Ixodes persulcatus*	SAMN02374895	Kurilshikov et al. (2014)
VS116(T)	*B. valaisiana*	1992	Switzerland	*Ixodes ricinus*	SAMN02436326	Schutzer et al. (2012)

Table S2A. Plasmids sequenced in this report (part 1)

Table S2
Plasmids sequenced in this study

	Strain	cp26	lp54	cp9-1	cp32-1	cp32-3	cp32-4	cp32-5	cp32-6	cp32-7	cp32-8	cp32-9	cp32-10	cp32-11	cp32-12	cp32-13	cp32-14	lp5
B. americana	SCW-30h	Present	Present					Present							Present			
B. americana	SCW-41	Present	Present							Present*						Present		
B. andersonii	MOD-5	Present	Present	Present	Present	Fused							Present	Present		Fused		
B. andersonii	21038	Present	Present							Present		Present	Present			Present		
B. bissettiae	PGeb	Present	Present	Present	Present			Present	Present	Fused*		Fused		Fused*	Fused	Fused		
B. burgdorferi	217_5#	Present	Present*					Present		Present		Fused	Fused					
B. burgdorferi	80a	Present	Present	Present (2)				Present	Present		Present		Present	Present	Present			
B. burgdorferi	Bol29#	Present	Present*				Fused	Fused				Present	Fused	Present	Present*			
B. burgdorferi	Fr-93-1	Present	Present					Fused		Present			Present	Fused	Fused			
B. burgdorferi	NE5248	Present	Present				Fused	Fused				Present		Fused	Present			
B. burgdorferi	NE5261	Present	Present				Fused	Present				Present	Present	Fused	Present			
B. burgdorferi	NE5267	Present	Present					Present				Present	Present	Present	Present*	Present*		
B. burgdorferi	NIH3	Present	Present				Fused	Present	Present	Present		Fused	Fused†	Present				
B. burgdorferi	NIH5	Present	Present*	Present*	Present*	Fused	Present	Present*	Present*	Fused	Present	Present		Present				
B. burgdorferi	NIH8	Present	Present	Fused			Present	Present	Present	Present		Present	Fused†	Present	Present			
B. burgdorferi	SCW-9#	Present*	Present*	Present			Present*	Fused*	Present*	Present*			Present	Present*	Fused*			
B. burgdorferi	Sh-2-82	Present	Present	Present	Present	Present	Present	Present	Present	Present		Present		Fused	Fused			
B. burgdorferi	Z9#	Present	Present	Present*				Present		Fused*		Fused*		Fused*				
B. californiensis	CA443	Present	Present			Present	Present*	Present†	Present		Fused*			Fused*	Present*	Present		
B. californiensis	CA446	Present	Present			Present	Present	Present†	Present		Present			Present	Present	Present		Present
B. caroliniensis	BUL-H-2	Present	Present				Present	Present		Present		Present		Present		Present	Present	
B. caroliniensis	M7p#	Present	Present*		Present*		Present*					Fused	Fused	Present				
B. caroliniensis	MI-3	Present	Present			Present	Fused†	Present		Present		Present	Present	Present	Present			
B. caroliniensis	SCGT-18	Present	Present			Present	Present	Present	Present	Present		Present	Present	Present	Present			
B. caroliniensis	SCW-22	Present	Present			Present	Present	Fused	Present			Fused	Fused	Fused		Present		
B. finlandensis	Z11	Present	Present			Fused*									Fused*			
B. japonica	HO14	Present	Present^	Present	Fused	Present	Present	Present†	Present*	Present		Present		Fused				
B. japonica	Miyazaki2E	Present	Present^	Present	Fused*	Present	Present		Fused	Fused		Present†	Fused*	Present	Present			
B. kurtenbachii	25015	Present	Present	Present		Present	Present	Present	Present		Present	Present		Present		Present		
B. lanii	CA-28-91	Present	Present					Present	Present	Present			Present					
B. lusitaniae	PoHL1	Dimer fusion	Present															
B. lusitaniae	PotiB2	Dimer fusion	Present			Fused†									Fused			
B. lusitaniae	PotiB3	Dimer fusion	Present		Present										Fused			
B. maritima	CA690	Present	Present							Present @					Present			
B. sinica	CMN3	Present	Present	Present	Present	Present		Present				Present			Fused			
B. spielmanii	PMew	Present	Present	Present		Present	Present	Present	Present*	Present			Fused*		Fused*			
B. tanukii	Hk501	Present	Present			Present			Present	Present		Present	Present	Present*				
B. tanukii	Koshiki4E	Present	Present			Present	Fused*	Present							Fused*			
B. tanukii	TanegashimaAS13	Present	Present		Present	Present*		Present		Present*		Present*	Present*					
B. tanukii	TanegashimaAS9	Present	Present		Present	Present		Fused	Fused	Present*		Fused		Fused*	Fused*			
B. turdi	047-3	Present	Present		Present		Present		Present					Present				
B. turdi	Ya501	Present	Present	Present	Present									Present				
B. valaisiana	100B40	Present	Present			Present			Present					Present				
B. valaisiana	Am501	Present	Present	Present	Present	Fused	Fused		Fused	Fused		Present						
B. yangtzensis	Miyako4E	Present	Present		Present*	Present		Present	Present	Fused		Present	Fused		Present			
B. yangtzensis	OkinawaCW61	Present	Present	Present	Fused	Fused	Present	Present	Fused	Present		Fused	Present	Fused	Fused			
B. yangtzensis	OkinawaCW62	Present	Present		Fused*	Present	Fused	Fused	Fused	Present		Fused	Fused*	Fused	Fused			

Table S2B. Plasmids sequenced in this report (part 2)

Table S2
Plasmids sequenced in this study

	Strain	lp17	lp21	lp25	lp28-1	lp28-2	lp28-3	lp28-4	lp28-5	lp28-6	lp28-7	lp28-8	lp28-9	lp28-12	lp28-13	lp28-14	lp36	lp38	lp56
B. americana	SCW-30h	Present		Present													Present	Present	
B. americana	SCW-41	Present		Present		Present		Present*				Present						Present*	
B. andersonii	MOD-5	Present						Present								Present	Present		
B. andersonii	21038	Present						Present			Present	Present							
B. bissettiae	PGeb	Present		Present			Present*		Present		Present*								Present
B. burgdorferi	217_5#	Present						Present									Present		
B. burgdorferi	80a	Present					Present*	Present*			Present	Present*					Present^	Present	
B. burgdorferi	Bol29#	Present					Present*	Present*					Present				Present		
B. burgdorferi	Fr-93-1	Present				Present	Present*				Present*		Present				Present		
B. burgdorferi	NE5248	Present				Present^	Present	Present					Present				Present		
B. burgdorferi	NE5261	Present				Present^	Present	Present					Present				Present		
B. burgdorferi	NE5267	Present				Present^	Present	Present					Present				Present		
B. burgdorferi	NIH3	Present		Present	Present*	Present		Present									Present	Present	Fused
B. burgdorferi	NIH5	Present		Present*	Present*	Present	Present	Present	Present*								Present	Present	
B. burgdorferi	NIH8	Present	Fused	Present	Present	Present	Present	Present									Present	Present	Fused
B. burgdorferi	SCW-9#	Present*		Present*	Present*	Present	Present	Present	Present*	Present*		Present*					Present*		Present*
B. burgdorferi	Sh-2-82	Present		Present	Present		Present	Present	Present	Present							Present	Present	
B. burgdorferi	Z9#	Present						Present				Present					Present		Present
B. californiensis	CA443	Present		Present							Present								
B. californiensis	CA446	Present		Present				Present			Present								
B. caroliniensis	BUL-H-2	Present		Present				Present			Present						Present		
B. caroliniensis	M7p#	Present							Present*	Present									
B. caroliniensis	MI-3	Present		Present			Present										Fused		
B. caroliniensis	SCGT-18	Present		Present		Present		Present	Present								Present		
B. caroliniensis	SCW-22			Present		Present	Present	Present	Present		Present						Present		
B. finlandensis	Z11	Present		Present				Present									Present*		
B. japonica	HO14	Present						Present$			Present	Present	Present				Present	Present	
B. japonica	Miyazaki2E	Present		Present				Fused$*			Present	Present					Present		
B. kurtenbachii	25015	Present		Present			Present			Present							Present		
B. lanii	CA-28-91	Present		Present*			Present	Present				Present*							
B. lusitaniae	PoHL1	Present		Present				Present$				Present*							
B. lusitaniae	PotiB2	Present		Present*				Fused$				Present						Present	Fused
B. lusitaniae	PotiB3	Present		Present				Fused$				Present							
B. maritima	CA690	Present				Present		Present									Present		
B. sinica	CMN3	Present						Fused$		Present							Present		
B. spielmanii	PMew	Present					Present	Present				Present					Present	Present	
B. tanukii	Hk501	Present					Present	Present$			Present						Present		Present
B. tanukii	Koshiki4E	Present		Present*			Present	Present$*			Present	Present					Present		Present
B. tanukii	TanegashimaAS13	Present		Present			Present	Present$*					Present				Present		Present
B. tanukii	TanegashimaAS9	Present					Present	Present$									Present		Present
B. turdi	047-3	Present					Present	Present				Present		Present			Present	Present	
B. turdi	Ya501	Present					Present*	Present*						Present	Present*		Present*	Present	
B. valaisiana	100B40	Present		Present ¢			Present										Present	Present	
B. valaisiana	Am501	Present		Present				Fused$		Present*		Present						Present	
B. yangtzensis	Miyako4E	Present		Present				Present$		Present			Present*				Present		
B. yangtzensis	OkinawaCW61	Present		Present*		Present*	Present	Present$									Present		Present*
B. yangtzensis	OkinawaCW62	Present		Present*		Present	Present	Fused$					Present				Present		Present

Table S2 legend. Plasmids sequenced in this report

The table shows the presence and absence of the 35 plasmid types (columns) in the 47 *Borreliella* genomes sequences presented in this report (rows). Plasmid names lp28-10 and cp32-2 are not used for historical reasons; no lp28-11 plasmids were found in these genomes. Some cp9 and all lp5 plasmids carry no PFam32 gene, and the only partitioning protein that the lp5 plasmids encode is a PFam57 protein. *B. burgdorferi* isolate 80a carries two cp9's with different PFam57 proteins. The lp5 in isolate CA446 is about 17 kbp in length and encodes a cp32-like PFam57 protein that may impart different compatibility from lp5 itself.

Table S2 footnotes:

\# Chromosome is in multiple contigs.

* These plasmid sequences are not complete. The sequence does not extend to the tip of one or more telomeres (in most cases comparison to closely related plasmids suggests that less than 200 bp are missing from these linear plasmid termini. In other incomplete cases contigs from plasmids that were predicted to be circular (*e.g.,* cp32-like plasmids) had no telomere consensus and no terminal direct repeat overlap and so were not circularizable.

$ These plasmids have syntenic gene contents with the cp32 plasmid family; however, they encode an lp28-4 type PFam32 protein is usually encoded by on circular cp32 plasmid (see text).

† These plasmids carry a PFam32 gene that is typical of, and named for a circular cp32 type on a linear lp32 plasmid.

^ These are "inverted repeat plasmids" with two identical or nearly identical halves that are inverted relative to one another (see text).

@ This plasmid was not present in the CA690 culture sequenced in this project, but was present in the CA690 culture sequenced independently by Margos *et al.* (2020) (accession number NZ_CP044542) where it was called "cp32-2" instead of "cp32-7".

¢ This plasmid was not present in the 100B40 genome sequenced in Maryland.

Table S3. Loci with high recombination rates among the North American *Borreliella species*

Locus	Replicon	Gene Description (gene name)
BB_0004	Chromosome	phosphoglucomutase
BB_0014	Chromosome	primosomal protein N
BB_0029	Chromosome	YaiI/YqxD family protein
BB_0030	Chromosome	signal peptidase I (*lepB-1*)
BB_0031	Chromosome	signal peptidase I (*lepB-2*)
BB_0036	Chromosome	DNA topoisomerase II (N-region) domain protein
BB_0081	Chromosome	efflux ABC transporter permease protein
BB_0100	Chromosome	glutamate racemase (*murI*)
BB_0101	Chromosome	asparaginyl-tRNA synthetase (*asnS*)
BB_0127	Chromosome	ribosomal protein S1
BB_0142	Chromosome	outer membrane efflux protein
BB_0152	Chromosome	glucosamine-6-phosphate isomerase (*nagB*)
BB_0153	Chromosome	superoxide dismutase
BB_0154	Chromosome	preprotein translocase SecA subunit (*secA*)
BB_0155	Chromosome	lipoprotein putative
BB_0156	Chromosome	conserved hypothetical protein
BB_0157	Chromosome	conserved hypothetical protein
BB_0158	Chromosome	S2 lipoprotein
BB_0201	Chromosome	UDP-N-acetylmuramyl-tripeptide synthetase
BB_0218	Chromosome	phosphate ABC transporter ATP-binding protein (*pstB*)
BB_0283	Chromosome	flagellar hook protein (*flgE*)
BB_0319	Chromosome	putative exported protein
BB_0330	Chromosome	bacterial extracellular solute-binding protein family 5
BB_0335-BB_0337	Chromosome	intergenic
BB_0342	Chromosome	glu-tRNA amidotransferase, subunit A (*gluA*)
BB_0348	Chromosome	pyruvate kinase (*pyk*)
BB_0401	Chromosome	glutamate transporter putative
BB_0457-BB_0459	Chromosome	intergenic
BB_0550	Chromosome	flagellar protein (*fliS*)
BB_0551	Chromosome	chemotaxis protein (*cheY*)
BB_0598	Chromosome	UDP-N-acetylenolpyruvoylglucosamine reductase (*murB*)
BB_0607	Chromosome	ATP-dependent DNA helicase
BB_0608	Chromosome	aminoacyl-histidine dipeptidase
BB_0608-BB_0610	Chromosome	intergenic
BB_0619	Chromosome	DHH domain protein
BB_0623	Chromosome	transcription-repair coupling factor (*mfd*)
BB_0632	Chromosome	exodeoxyribonuclease V alpha subunit (*recD*)
BB_0633	Chromosome	exodeoxyribonuclease V beta subunit (*recB*)
BB_0635	Chromosome	nicotinate phosphoribosyltransferase (*pncB*)
BB_0636	Chromosome	glucose-6-phosphate 1-dehydrogenase (*zwf*)
BB_0664	Chromosome	putative lipoprotein

Table S3 cont.

Locus	Replicon	Gene Description (gene name)
BB_0684	Chromosome	isopentenyl-diphosphate delta-isomerase type 2 (*fni*)
BB_0796	Chromosome	conserved hypothetical protein
BB_0797	Chromosome	DNA mismatch repair protein (*mutS*)
BB_0807	Chromosome	conserved hypothetical integral membrane protein
BB_0808	Chromosome	putative permease YjgP/YjgQ family
BB_0841	Chromosome	arginine deiminase (*arcA*)
tRNA	Chromosome	RNA gene
tRNA	Chromosome	RNA gene
BB_A09	lp54	conserved hypothetical protein
BB_A10	lp54	conserved hypothetical protein
BB_A11	lp54	conserved hypothetical protein
BB_A12	lp54	holin BlyA family
BB_A24	lp54	decorin-binding protein A (*dbpA*)
BB_A25	lp54	decorin-binding protein B (*dbpB*)
BB_B17	cp26	inosine-5'-monophosphate dehydrogenase (*guaB*)
BB_B18	cp26	GMP synthase (*guaA*)
BB_B19	cp26	outer surface protein C (*ospC*)
BB_B22	cp26	putative guanine/xanthine permease
BB_B26	cp26	conserved hypothetical protein
BB_D13	lp17	conserved hypothetical protein
BB_D21	lp17	plasmid partition protein

Table S4. Introgressed loci analysis with four-taxon *D*-statistics

**Locus**	**Replicon**	**Species P1**	**Species P3**	***D* Total**	***P* value**
BB_0082	Chromosome	N40	SCW-41	59	1.57 e-14
BB_0098	Chromosome	N40	SCW-41	31	2.58 e-08
BB_0122	Chromosome	29805, N40	SCW-41	32	1.54 e-08
BB_0122	Chromosome	B31, 64b, CA382, NIH3, NIH5, NIH8	SCW-41	34	5.51 e-09
BB_0127	Chromosome	B331	SCW-41	32	1.54 e-08
BB_0201	Chromosome	29805	SCW-41	42	9.12 e-11
BB_0201	Chromosome	N40	SCW-41	31	2.58 e-08
BB_0217	Chromosome	CA8	SCW-41	32	1.54 e-08
BB_0218	Chromosome	CA8	SCW-41	35	3.29 e-09
BB_0220	Chromosome	CA8	25015	40	2.53 e-10
			DN127	40	2.53 e-10
			SCGT-18	44	3.28 e-11
BB_0236	Chromosome	118a, 156a, 29805, 72a, 80a, 94a, MM1, N40	SCW-41	33	9.21 e-09
BB_0252	Chromosome	80a	SCW-41	32	1.54 e-08
BB_0341	Chromosome	CA8	SCW-41	56	7.25 e-14
BB_0342	Chromosome	CA8	SCW-41	64	1.22 e-15
BB_0342	Chromosome	72a, 94a, MM1, 80a	SCW-41	56	7.24 e-14
			SCW-41	54	2.0 e-13
BB_0348	Chromosome	29805, N40	CA690	38	7.97 e-10
BB_0369	Chromosome	118a, 156a, 29805, 72a, 80a, CA-11-2A, JD1, N40, Sh-2-82	SCW-41	33	9.21 e-09
BB_0389	Chromosome	B331, CA8	CA-28-91, DN127, MN14-1420, MN14-1539, SCW-41	32	1.54 e-08
BB_0436	Chromosome	156a, 29805, 94a, CA382, CA8, JD1, MM1, N40, Sh-2-82	CA690	31	2.58 e-08
BB_0680	Chromosome	CA382	DN127	31	2.58 e-08
BB_0684	Chromosome	156a, 29805, 80a, N40	SCW-41	37	1.81 e-09
BB_0685	Chromosome	29805, JD1, N40	SCW-41	33	9.21 e-09
BB_0830	Chromosome	80a, 94a, CA8, JD1, MM1	25015	31	2.58 e-08

[a] Loci with definitive P1 and P3 species candidates and significant *D*-statistics

Table S5A. Divergence times and ancestral population sizes

Species	Mean divergence time from MRCA (95% HPD) (Ma, μ= 1x10^{-12} per/site/generation)	Mean divergence time from MRCA (95% HPD) (Ma, Root calibration=180-55Ma)	Mean Effective population size (95% HPD) (N_e, μ = 1x10^{-12} per/site/generation)
B. afzelii	56.4 (3.73-144.7)	71.4 (34.2-106.5)	1.4 x10^{9} (3.4 x10^{8}-3.2 x10^{9})
B. americana	30.7 (1.94-78.8)	38.9 (18.5-58.3)	4.6 x10^{9} (1.1 x10^{9}- 1x10^{10})
B. andersonii	38.8 (2.6-99.7)	49.1 (23.5-73.2)	3.2 x10^{9} (8 x10^{8}-7.3 x10^{9})
B. bavariensis	16.3 (1.03-41.8)	20.7 (9.9-31)	1.4 x10^{10} (3.1 x10^{9}-3.2 x10^{10})
B. bissettiae	13.9 (0.99-3.59)	17.7 (8.49-26.4)	2.5 x10^{8} (5.8 x10^{7}-5.6 x10^{8})
B. burgdorferi	14.8 (0.96-37.9)	18.7 (8.98-28)	2.4 x10^{9} (5.7 x10^{8}-5.3 x10^{9})
B. californiensis	40.6 (2.9-104.4)	51.4 (24.5-76.4)	8.8 x10^{7} (2.1 x10^{7}-2 x10^{8})
B. carolinensis	13.9 (0.99-3.59)	17.7 (8.49-26.4)	4.9 x10^{9} (1.2 x10^{9}-1 x10^{10})
B. chilensis	92.8 (6.14-238.4)	117.6 (58.4-177.1)	N/A
B. finlandensis	14.8 (0.96-37.9)	18.7 (8.98-28)	2.8 x10^{9} (6.5 x10^{8}-6.3 x10^{9})
B. garinii	16.3 (1.03-41.8)	20.7 (9.9-31)	2.5 x10^{9} (6.1 x10^{8}-5.7 x10^{9})
B. japonica	67.3 (4.08-172.3)	85.2 (41.1-127.2)	2.3 x10^{9} (5.6 x10^{8}-5.1 x10^{9})
B. kurtenbachii	25.3 (1.74-6.5)	32 (15.3-47.7)	N/A
B. lanei	39.5 (2.5-101.3)	50 (23.9-74.5)	N/A
B. lusitaniae	71.3 (5.06-183.5)	90.3 (43.6-134.8)	3.4 x10^{9} (8.1 x10^{8}-7.6 x10^{9})
B. mayonii	55.5 (3.74-142.4)	70.2 (33.9-104.8)	9.9 x10^{7} (2.3 x10^{7}-2.2 x10^{8})
B. maritima	77.8 (4.7-199.4)	98.5 (47.1-146.6)	N/A
B. sinica	67.3 (4.08-172.3)	85.2 (41.1-127.2)	N/A
B. spielmanii	56.4 (3.73-144.7)	71.4 (34.2-106.5)	1.2 x10^{8} (2.8 x10^{7}-2.7 x10^{8})
B. tanukii	59.1 (4.14-152)	74.9 (36.3-112)	1.7 x10^{9} (5.7 x10^{8}-5.3 x10^{9})
B. turdi	69.9 (4.15-179.1)	88.5 (42.5-132)	3.6 x10^{9} (4 x10^{8}-3.7 x10^{9})
B. valaisiana	36 (2.37-92.4)	45.6 (22-68.1)	5.8 x10^{9} (1.4 x10^{9}-1.3 x10^{10})
B. yangtzensis	36 (2.37-92.4)	45.6 (22-68.1)	2.5 x10^{9} (6 x10^{8}-5.6 x10^{9})

Table S5B. Biogeographic models tested using BioGeoBears

Model	LnL	# parameters	AIC	AICc
DEC	-36.53	2	77.05	77.65
DEC+J	-35.49	3	76.97	78.24
DIVALIKE	-42.02	2	88.04	88.64
DIVALIKE+J	-37.91	3	81.81	83.08
BAYAREALIKE	-43.22	2	90.44	91.04
BAYAREALIKE+J	-37.91	3	81.81	83.08

Figure S1. *Borreliella* chromosome terminal extensions

S1A. North American clade chromosomal extensions

S1B. Eurasian clade chromosomal extensions

Maps of the chromosome left and right end regions of newly sequenced genomes are shown with genes indicated by boxes with a pointed end that indicates the direction of transcription. Yellow, orange and light blue genes at the left ends are homologs of *B. burgdorferi* strain B31 genes *bb_001, bb_002* and *bb_003*, respectively, and pink and blue-green genes at the right ends are homologs of *B. burgdorferi* strain B31 genes *bb_842* and *bb_843*, respectively. Light green genes are those that are present in the terminal extensions. Isolate names are given in center of the maps. Large asterisks indicate that the sequence extends to the tip of closed hairpin telomere (or sequence end where telomere has not been sequenced) and the numbers at the ends of the chromosomes are the distances from the telomere tip to the *bb_001* gene homolog on the left (except in *B. valaisiana* VS116 and 100B40 where it is to *bb_002*) and between telomere tip and *bb_843* (including its stop codon) on the right. Kbp scales are indicated above with bp 1 as the start of the homologs of genes *bb_003* and *bb_842,* for the left and right ends, respectively. Numbers above the genes are gene lengths (bp) that include the stop codon. Paralogous protein families (PF) (Casjens *et al*., 2000; 2012) are indicated above the genes in the terminal extensions where small asterisks indicate pseudogenes. Backslashes indicate frameshift differences that could be either accurate pseuodgenes or sequencing errors. The constant region of the *Borreliella* chromosome typically (except in some *B. valaisiana* isolates, above) extends from *bb_001* through *bb_843* (strain B31 gene names).

In addition to the newly sequenced isolates, *B. burgdorferi* strain B31and *B. valaisiana* VS116 and are shown for comparison in panels **A** and **B**, respectively. No new *B. burgdorferi* sensu stricto right end extension types were found among the newly sequenced genomes; they have the following extension types (see type names figure 6 of Casjens *et al.* 2017): S1 type, 80a; M-1 type, NIH3, NIH5, NIH8, Bol29, Fr-93-1, NE_5248, NE_5261 and NE_5267; type M-3; Z9, 217_5 and SCW-9 (the latter has about 300 bp of unique DNA at its tip); type L-2, Sh-2-82. The *B. carolinensis* M7p chromosome sequence did not reach either telomere and is not shown in the figure.

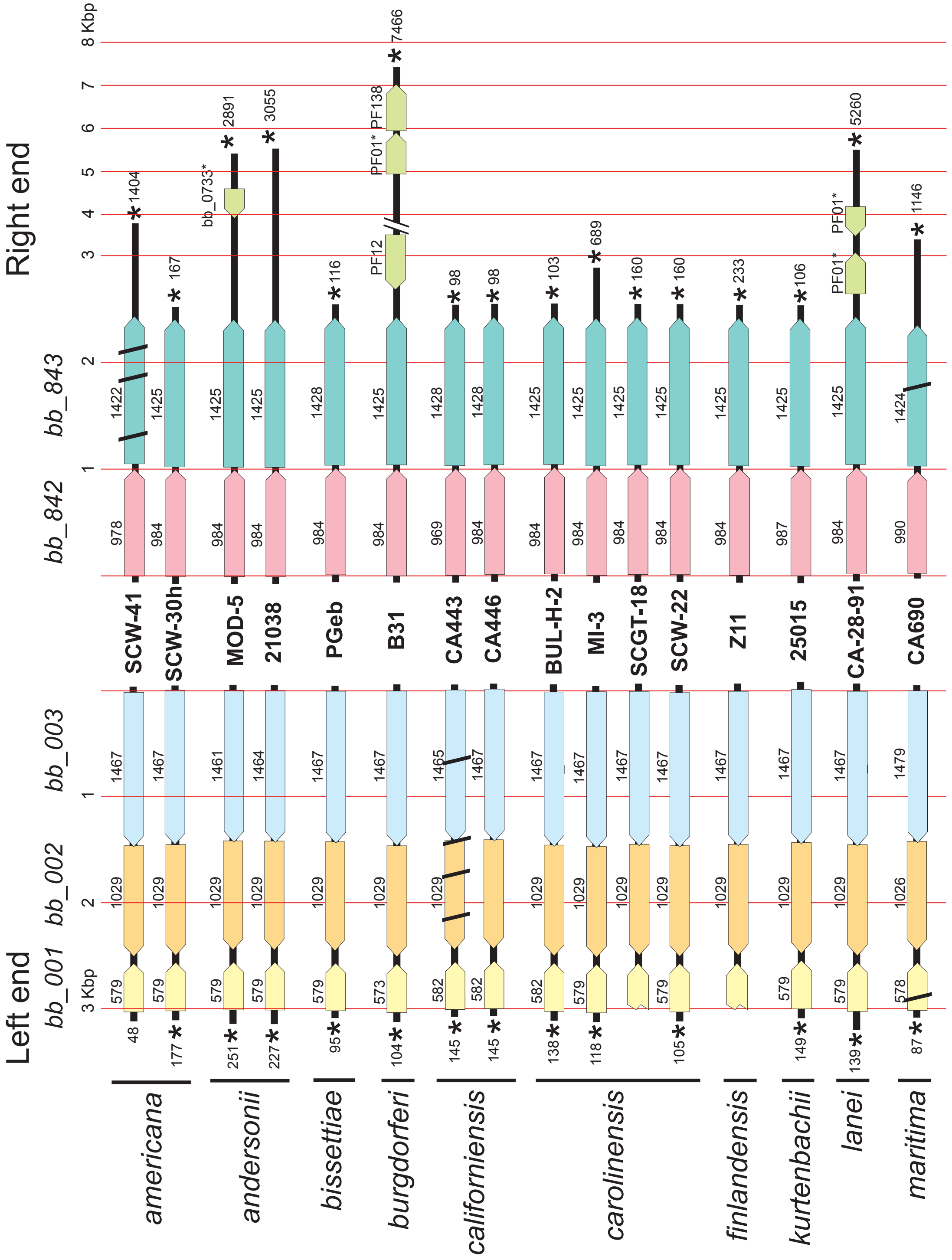
Right end
Left end
bb_843
bb_842
bb_003
bb_002
bb_001
8 Kbp
3 Kbp
SCW-41
SCW-30h
MOD-5
21038
PGeb
B31
CA443
CA446
BUL-H-2
MI-3
SCGT-18
SCW-22
Z11
25015
CA-28-91
CA690
americana
andersonii
bissettiae
burgdorferi
californiensis
carolinensis
finlandensis
kurtenbachii
lanei
maritima
bb_0733*
PF01*
PF138
PF12

Figure S1A

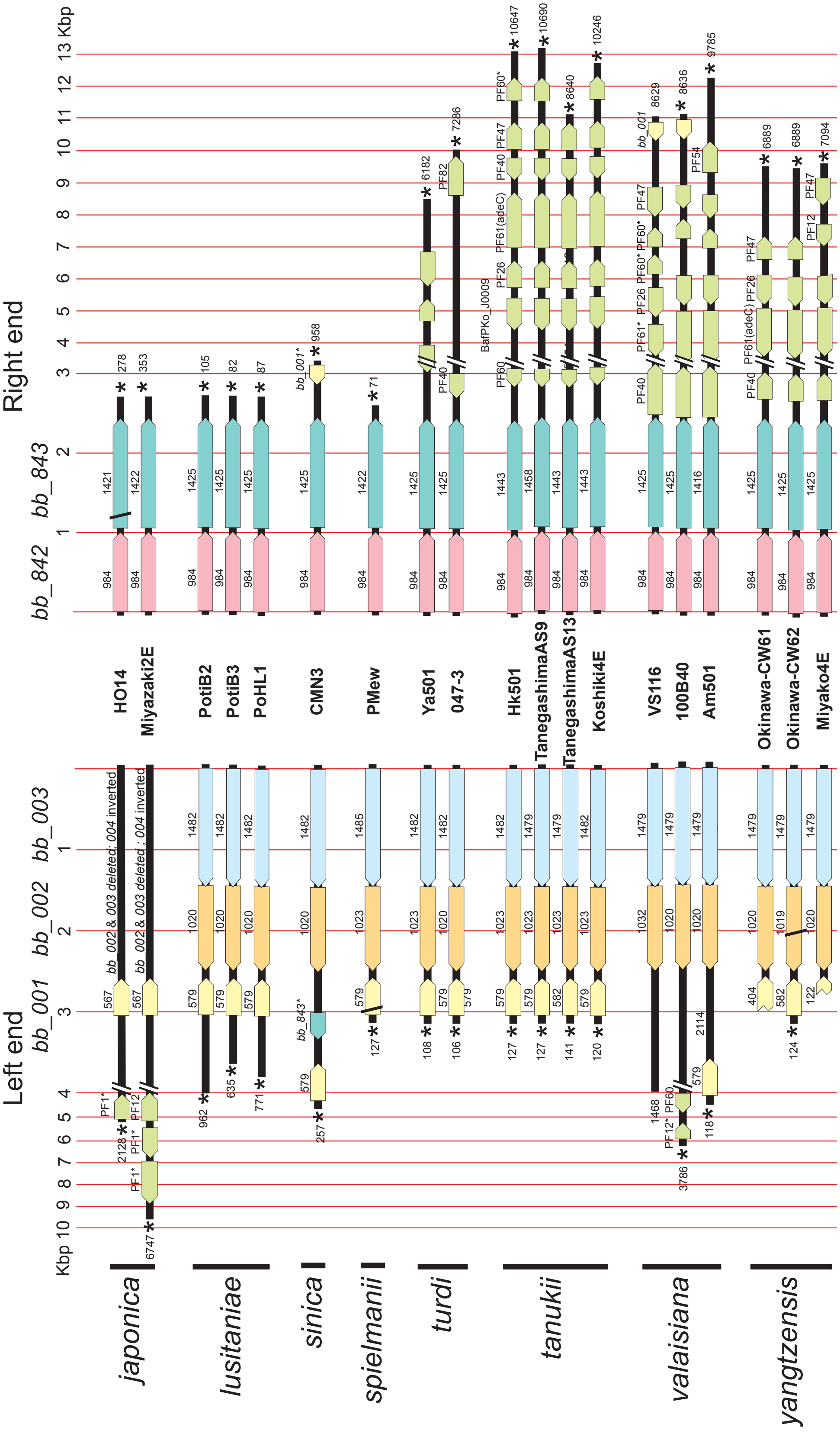
Right end
Left end
bb_842
bb_843
bb_001
bb_002
bb_003
13 Kbp
Kbp 10
HO14
Miyazaki2E
PotiB2
PotiB3
PoHL1
CMN3
PMew
Ya501
047-3
Hk501
TanegashimaAS9
TanegashimaAS13
Koshiki4E
VS116
100B40
Am501
Okinawa-CW61
Okinawa-CW62
Miyako4E
bb_002 & 003 deleted; 004 inverted
japonica
lusitaniae
sinica
spielmanii
turdi
tanukii
valaisiana
yangtzensis

Figure S1B

Figure S2. *Borreliella* plasmid PFam32 protein neighbor-joining tree

Selected amino acid sequences from all the currently known *Borreliella* plasmid PFam32 protein types were aligned, and an unrooted neighbor-joining tree was constructed by Clustal X (Larkin *et al.*, 2007). The different PFam32 types are highlighed with different background colors. Bootstrap values from 1000 trials are shown above the branches, and branches with bootstrap values below 950 were collapsed to multi-branch points. A fractional distance scale bar is shown at the upper left. Plasmid names are indicated at the right of each branch in large text, and the selected *Borreliella* isolates carrying them are indicated in small text at the branch tips; red isolate names denote those with different geometry (linear or circular) from others in the same PFam32 type. Red stars mark the four new PFam32 types, cp32-14, lp28-12, -13 and -14 discovered in this study. The green star marks lp28-11 whose PFam32 relationship not been previously published (see Schwartz *et al.,* 2021); it is the same type as the protein encoded by the second “orphan” PFam32 gene on strain B31 lp28-1 that was previously not given a PFam32 type name.

We also note that in the over *Borreliella* 900 plasmids that we have sequenced (this report and Casjens *et al.*, 2017 and 2018) there is only one instance of two *apparently intact* PFam32 genes of the same type in the same genome. In this unique and unsubstantiated instance *B. yangtzensis* Okinawa-CW61 contains two cp32-11 type PFam32 genes. Given the extreme rarity of such an occurrence, we suggest that the cp32-11 PFam32 gene or its expression in this isolate’s cp32-9+11 plasmid might have been inactivated by point mutation, since this plasmid also carries a cp32-9 type PFam32 gene which could mediate its partition.

Figure S2

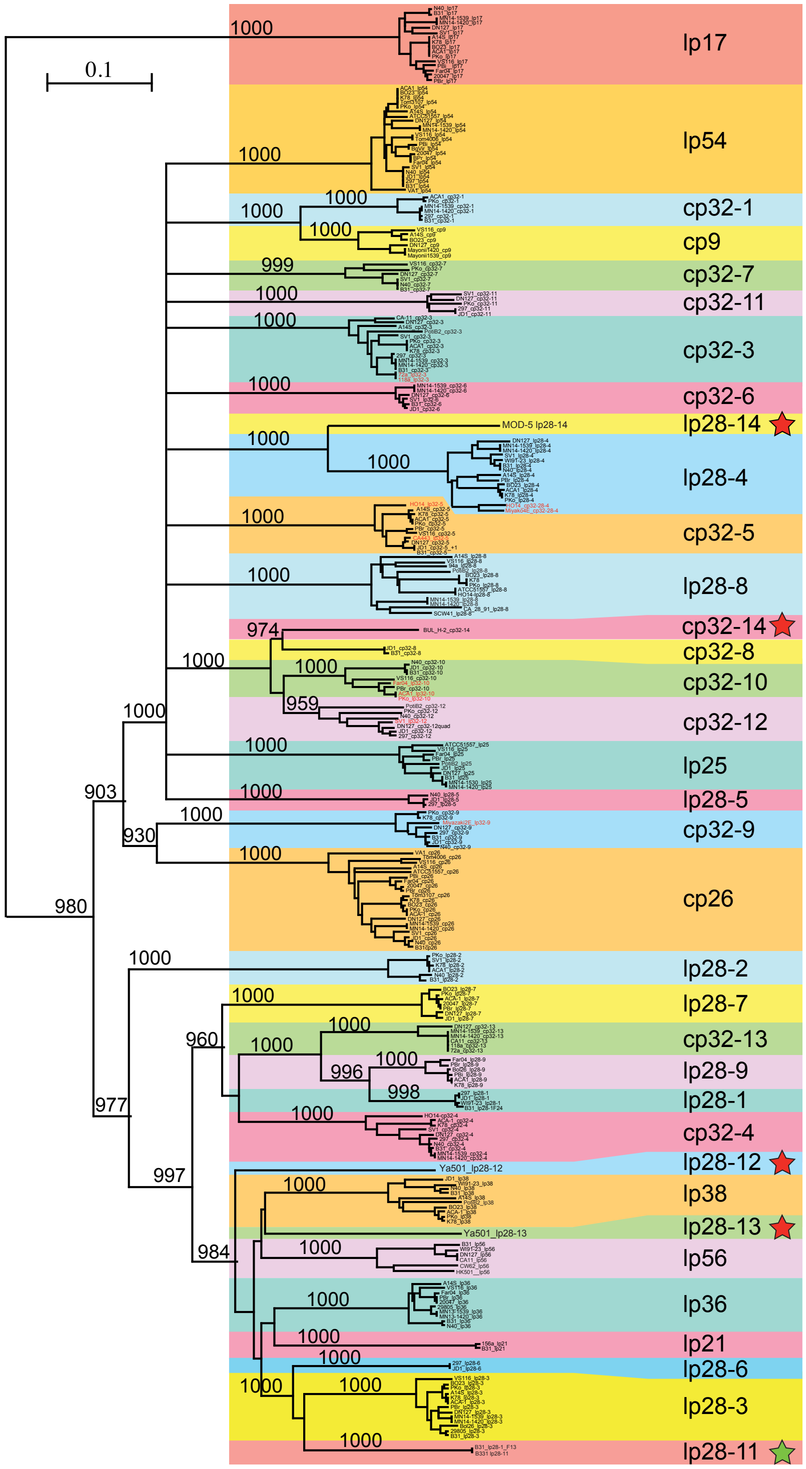
0.1
lp17
lp54
cp32-1
cp9
cp32-7
cp32-11
cp32-3
cp32-6
lp28-14
lp28-4
cp32-5
lp28-8
cp32-14
cp32-8
cp32-10
cp32-12
lp25
lp28-5
cp32-9
cp26
lp28-2
lp28-7
cp32-13
lp28-9
lp28-1
cp32-4
lp28-12
lp38
lp28-13
lp56
lp36
lp21
lp28-6
lp28-3
lp28-11
MOD-5 lp28-14
Ya501_lp28-12
Ya501_lp28-13

Figure S3. *B. californiensis* CA446 linear plasmid lp5

A reading frame map of *B. californiensis* isolate CA446 lp5 is shown above with the five apparently intact large reading frames indicated by green filling. Stop codons are indicated by vertical lines that span the frame rectangle, and potential start codons are indicated by short vertical lines. The maps were created with DNA Strider (Douglas, 1994). PFam numbers are shown above, where asterisks (*) mark truncated pseudogenes. The protein encoded by the ORF marked "BLA32_05550" is a rare gene type in *Borreliella* that is 93% identical to a protein with that locus_tag encoded by an unnamed plasmid in *B. afzelii* isolate BO23 (accession number CP018269). Below a dotplot shows the two complex repeat regions.

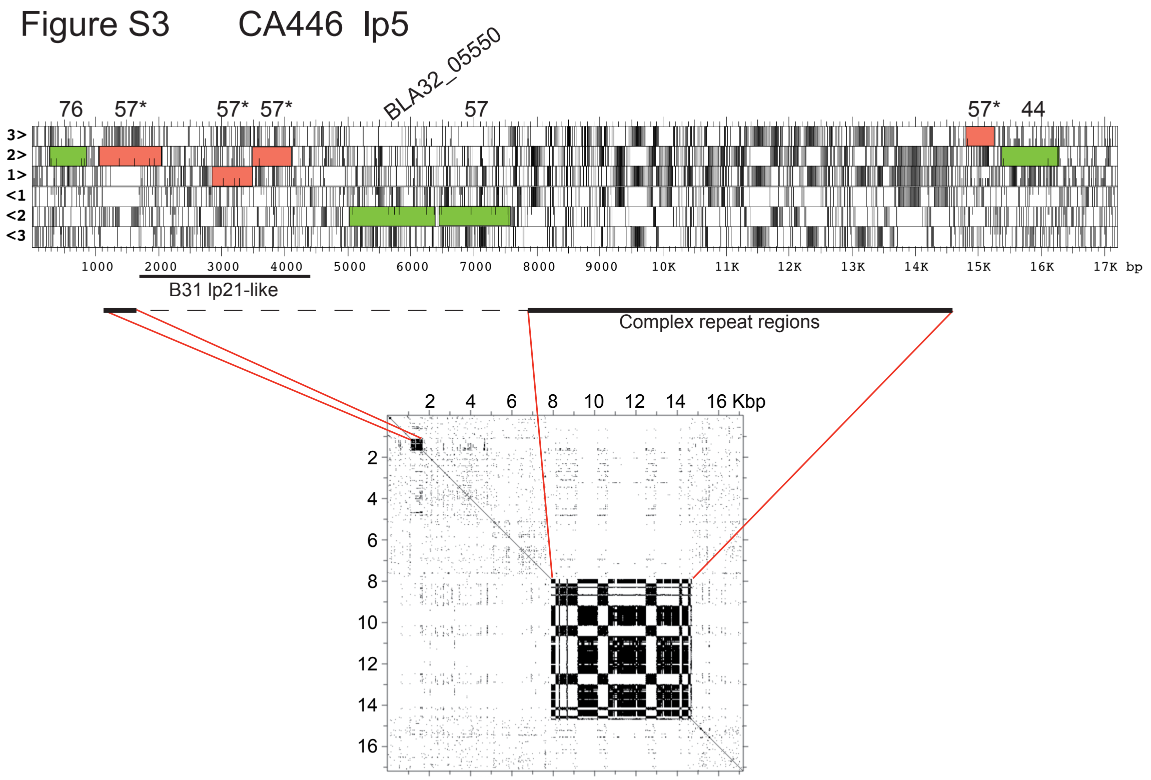

Figure S4A. Recombination rates and hotspots on the *B. burgdorferi* chromosome and plasmids cp26, lp17 and lp54

In the left panels black lines show the posterior mean per-site recombination rates (y-axis) estimated with LDhat (McVean *et al*., 2002) based on bi-allelic SNV segregation plotted against the location in the genome (x-axis) (see text), and red dotted lines show the 95% confidence intervals. In this figure, seven loci and one intergenic region (BB_0608-BB_0610) from chromosome, three loci from cp26, one locus from lp17 and two loci from lp54 with high mean recombination rates peaks are highlighted in orange color. Table S3 lists all the loci with high mean recombination rates located on the four replicons.

The right panels are boxplots that show distributions of per-site recombination rates (y-axis) at bi-allelic SNV sites on the four replicons (x-axis). Significant difference in the mean recombination rates observed between the chromosome and the plasmids (chromosome vs cp26: p = 1.37e-07, chromosome vs lp17: p = 1.46e-31, chromosome vs lp54: p = 1.38e-83, all by *t*-tests) and between pairs of plasmids (cp26 vs lp17: p = 1.51e-10, cp26 vs lp54: p = 2.08e-77, lp17 vs lp54: p = 1.03e-104, all by t-tests). Sites with high recombination rates (outliers of each boxplot) were excluded from subsequent phylogenetic analyses of *B. burgdorferi* genomes.

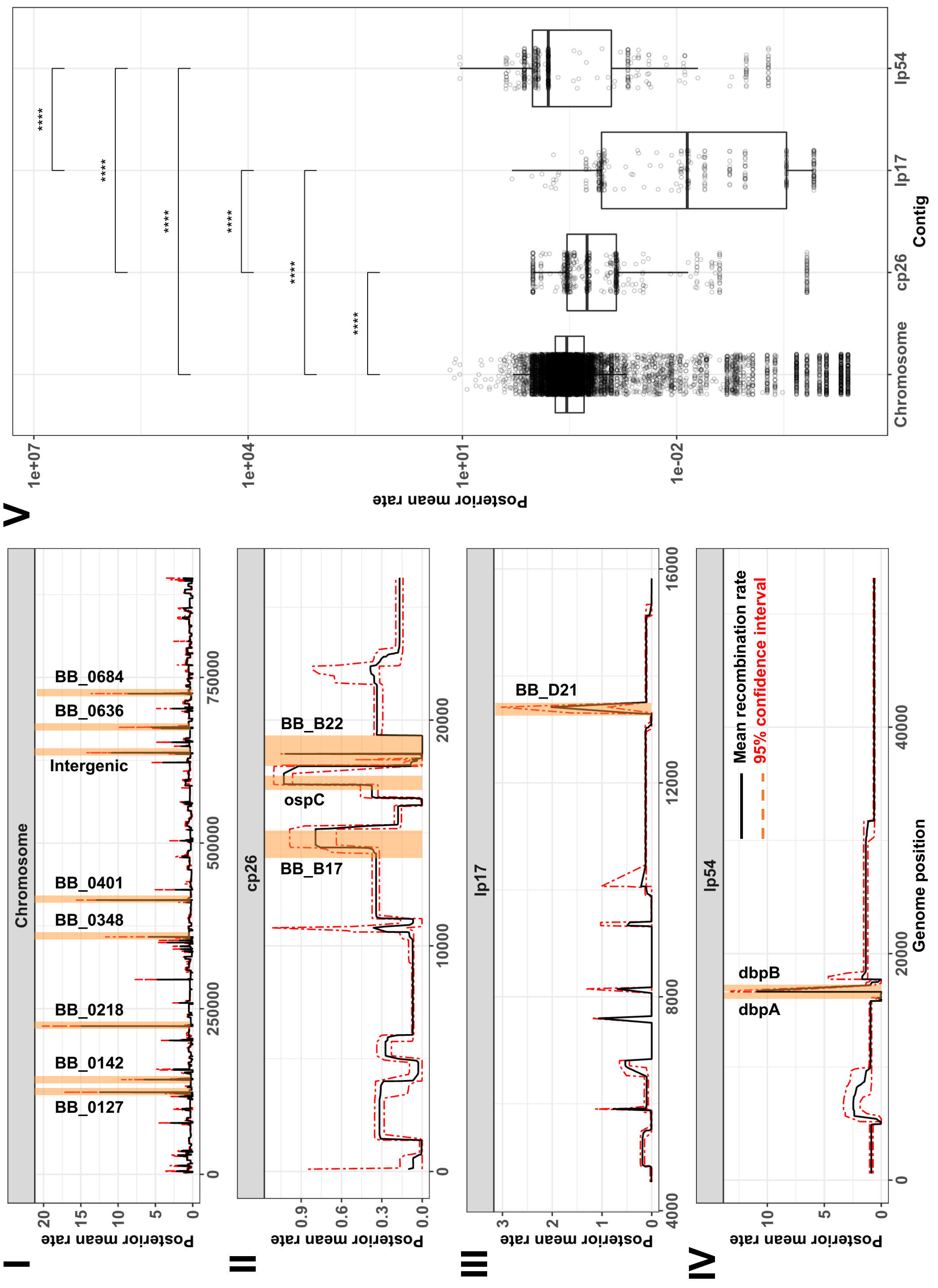
I
II
III
IV
V
Chromosome
BB_0127
BB_0142
BB_0218
BB_0348
BB_0401
Intergenic
BB_0636
BB_0684
cp26
BB_B17
ospC
BB_B22
lp17
BB_D21
lp54
dbpA
dbpB
Mean recombination rate
95% confidence interval
Posterior mean rate
Genome position
Contig
Chromosome
cp26
lp17
lp54
****
1e+07
1e+04
1e+01
1e-02

Figure S4B. A cross-species genetic exchange (introgression) between species *B. burgdorferi* and *B. americana*

The figure shows a partial alignment of North American *Borreliella* genomes at *bb_0082* (B31 gene coordinates 1130 to 1189) on the chromosome. Dots (.) in sequences in the figure indicate the same nucleotide as the corresponding site in the B31 sequence. Isolates B31, NE5621, N40 and JD1 represent species *B. burgdorferi*, SV1 represents *B. finlandensis*, SCW-30h and SCW-41 represent *B. americana*, DN127 represents *B. bissettiae*, SCW-22 represents *B. carolinensis*, 25015 represents *B. kurtenbachii*, CA443 represents *B. californiensis*, MN14-1539 represents *B. mayonii*, and CA690 represents the North American basal species *B. maritima*. All other available orthologous sequences in the public database from *B. burgdorferi* isolates are identical to that of B31, except those from isolates 88a and 167bjm (Qui *et al.*, 2004) which are identical to that of N40. Only the *bb_0082* gene has been sequenced from the latter two isolates (88a and 167bjm), but we suggest that their whole chromosomes are likely to be very close relatives of N40 since their OspC proteins are identical to that of N40 (Qui *et al.*, 2004) and overall, OspC types are closely associated with chromosomal sequence type (see article text). *B. burgdorferi* N40 displays an apparent phylogenetic inconsistency, showing a higher sequence identity to the *B. americana* isolates than to con-specific *B. burgdorferi* isolates (highlighted by red boxes), suggesting a single introgression event from *B. americana* to the N40 strain lineage.

The four-taxon D-statistical analysis (see text) using isolate N40 as P1 (*B. burgdorferi*), SV1 as P2 (*B. finlandensis*), SCW-41 as P3 (*B. americana*) and VA1 as P0 (*B. chilensis*; sequence not shown in figure) successfully identified the signature of cross-species hybridization between P1 and P3, two North American species with a p=1.57E-14 (Table S4).

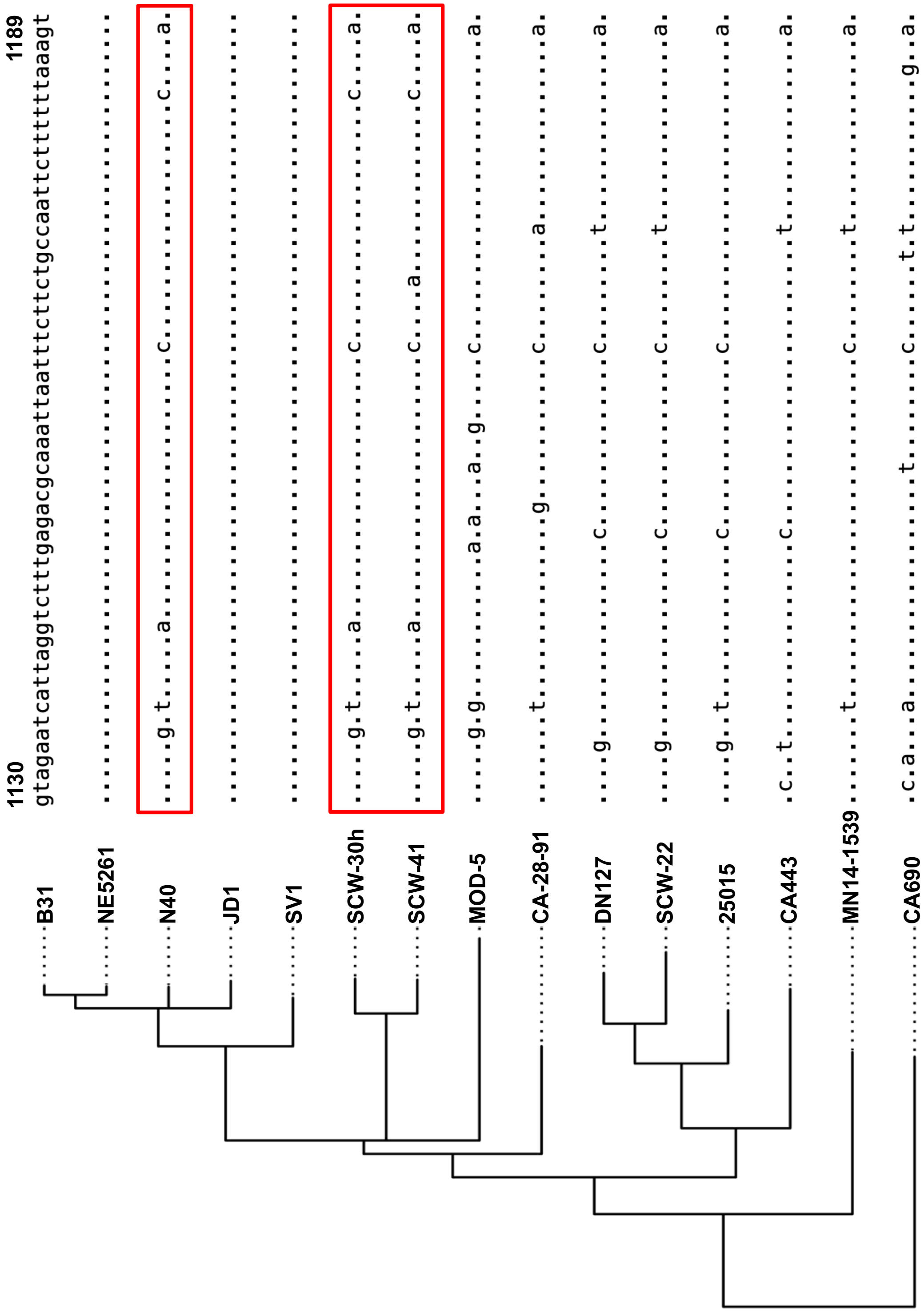
1130
1189
B31
NE5261
N40
JD1
SV1
SCW-30h
SCW-41
MOD-5
CA-28-91
DN127
SCW-22
25015
CA443
MN14-1539
CA690

Figure S5. Phylogeny of the circular plasmid cp26

A. A phylogenic tree of cp26 plasmid sequences from 23 *Borreliella* species.

The tree was constructed by IQ-tree as described in the text and MATERIALS AND METHODS. *B. burgdorferi* is represented by a single cp26 (strain B31) for clarity of presentation. All branches shown are supported by a bootstrap value of 80% or higher. The tree was rooted with the *B. chilensis* VA1 cp26 as the outgroup. The branch tip circles are colored according to genome sequencing status (present here or previously sequenced), and the heatmap on the right indicates the geographic origins of the sequenced strains. Branches are labeled with species designations. Only three *B. burgdorferi* are shown, but see part B below. Asterisks (*) indicate a new OspC type which we name "NE". Also note that isolate ZS7 OspC is indicated by "B", although it is a European restricted subtype of B called B2 (Qiu et al., 2008) or Bb (Travinsky et al., 2010). For the dimerized cp26 plasmids in the three *B. lusitaniae* genomes (see text), the variant-call pipeline maps of the cp26 monomer that was the closest to the B31 cp26 reference sequence was used.

B. A phylogenetic tree of cp26 sequences from 28 *B. burgdorferi* isolates. *B. burgdorferi* isolates were extracted from the tree described in figure S5A. The first and second columns of the heatmap on the right indicate the geographic origins of the isolates and the within-species *B. burgdorferi* SNV groups, respectively. The scale bars indicate changes per site.

Figure S5

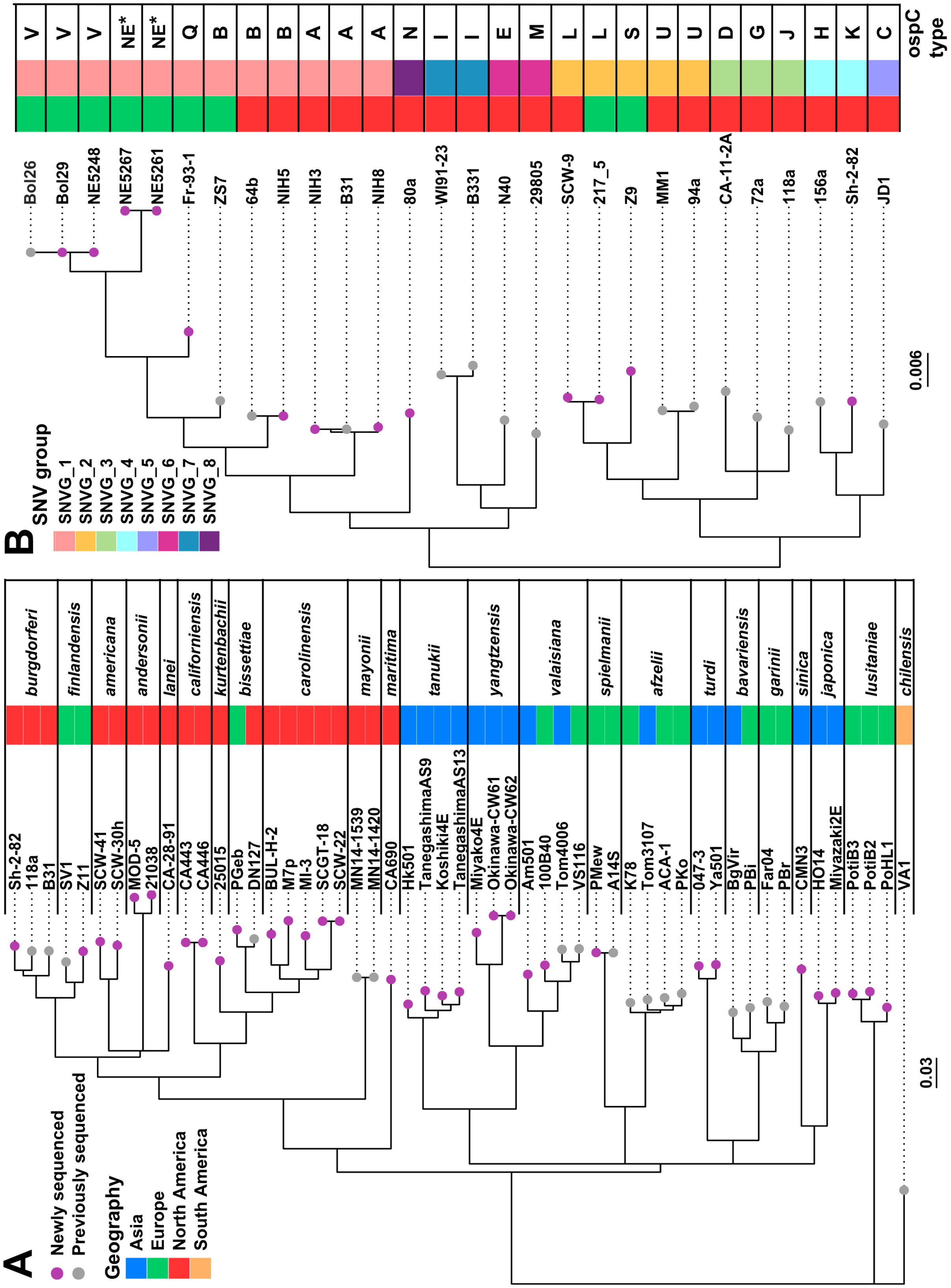
A
Newly sequenced
Previously sequenced
Geography
Asia
Europe
North America
South America
0.03
Sh-2-82
118a
B31
SV1
Z11
SCW-41
SCW-30h
MOD-5
21038
CA-28-91
CA443
CA446
25015
PGeb
DN127
BUL-H-2
M7p
MI-3
SCGT-18
SCW-22
MN14-1539
MN14-1420
CA690
Hk501
TanegashimaAS9
Koshiki4E
TanegashimaAS13
Miyako4E
Okinawa-CW61
Okinawa-CW62
Am501
100B40
Tom4006
VS116
PMew
A14S
K78
Tom3107
ACA-1
PKo
047-3
Ya501
BgVir
PBi
Far04
PBr
CMN3
HO14
Miyazaki2E
PotiB3
PotiB2
PoHL1
VA1
burgdorferi
finlandensis
americana
andersonii
lanei
californiensis
kurtenbachii
bissettiae
carolinensis
mayonii
maritima
tanukii
yangtzensis
valaisiana
spielmanii
afzelii
turdi
bavariensis
garinii
sinica
japonica
lusitaniae
chilensis
B
SNV group
SNVG_1
SNVG_2
SNVG_3
SNVG_4
SNVG_5
SNVG_6
SNVG_7
SNVG_8
0.006
Bol26
Bol29
NE5248
NE5267
NE5261
Fr-93-1
ZS7
64b
NIH5
NIH3
B31
NIH8
80a
WI91-23
B331
N40
29805
SCW-9
217_5
Z9
MM1
94a
CA-11-2A
72a
118a
156a
Sh-2-82
JD1
V
V
V
NE*
NE*
Q
B
B
B
A
A
A
N
I
I
E
M
L
L
S
U
U
D
G
J
H
K
C
ospC type

Figure S6. Phylogeny of the linear plasmid lp54

A. phylogenetic tree of lp54 plasmids from 23 *Borreliella* species.

The tree was constructed and presented as described in the legend to figure S5A. The heatmap to the right represents the geographic origins of the sequenced strains. Branches are labeled with species designations. Inconsistencies with the chromosomal tree (figure 4 in this report) are discussed in the text of the paper.

B. A phylogenetic tree of lp54 sequences from 29 *B. burgdorferi* isolates.

The tree was constructed and presented as described in the legend to figure S5A. The first and second columns of the heatmap on the right indicate the geographic origins of the sequenced strains and the within-species SNV groups, respectively.

Figure S6

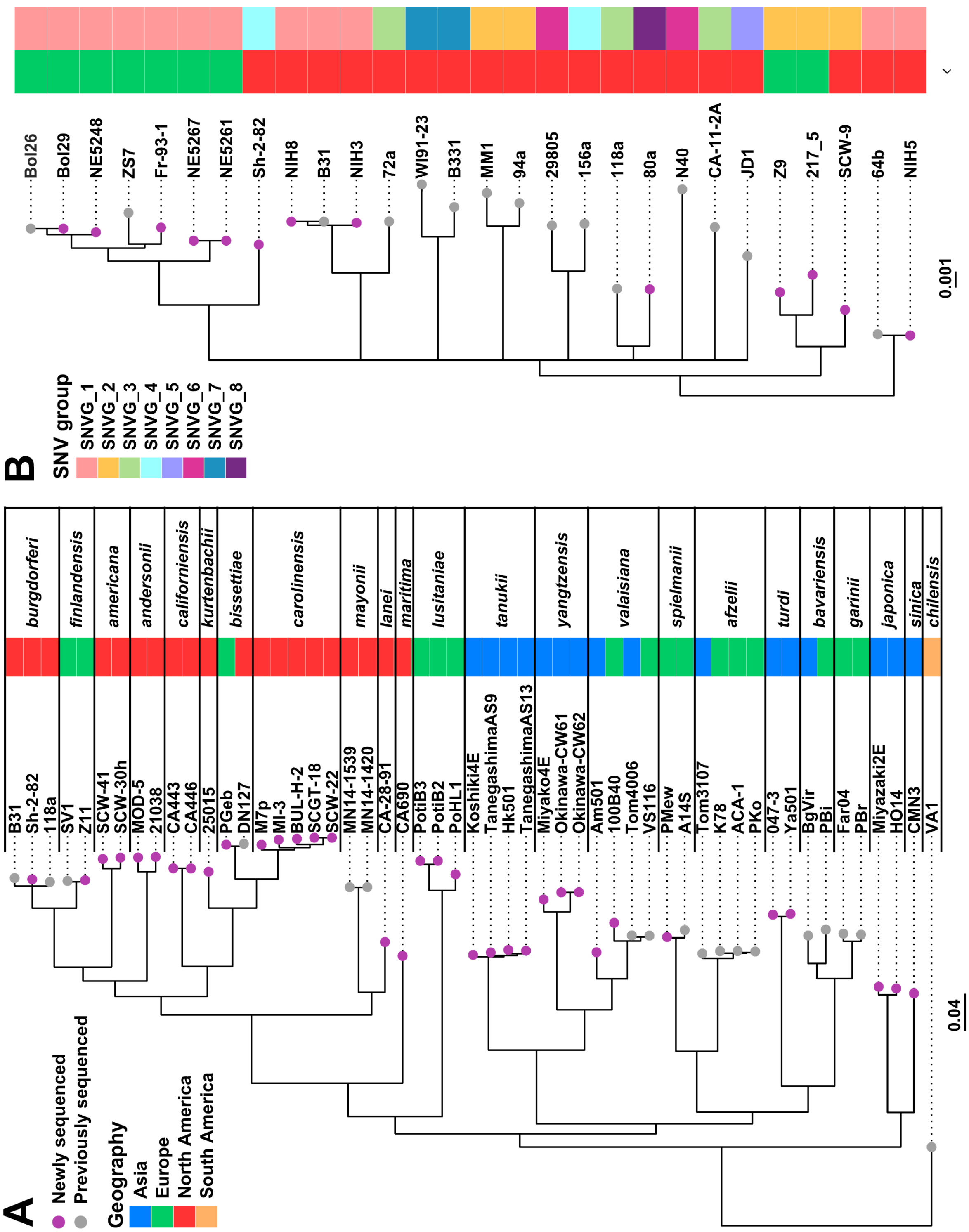
A
Newly sequenced
Previously sequenced
Geography
Asia
Europe
North America
South America
burgdorferi
finlandensis
americana
andersonii
californiensis
kurtenbachii
bissettiae
carolinensis
mayonii
lanei
maritima
lusitaniae
tanukii
yangtzensis
valaisiana
spielmanii
afzelii
turdi
bavariensis
garinii
japonica
sinica
chilensis
B31
Sh-2-82
118a
SV1
Z11
SCW-41
SCW-30h
MOD-5
21038
CA443
CA446
25015
PGeb
DN127
M7p
MI-3
BUL-H-2
SCGT-18
SCW-22
MN14-1539
MN14-1420
CA-28-91
CA690
PotiB3
PotiB2
PoHL1
Koshiki4E
TanegashimaAS9
Hk501
TanegashimaAS13
Miyako4E
Okinawa-CW61
Okinawa-CW62
Am501
100B40
Tom4006
VS116
PMew
A14S
Tom3107
K78
ACA-1
PKo
047-3
Ya501
BgVir
PBi
Far04
PBr
Miyazaki2E
HO14
CMN3
VA1
0.04
B
SNV group
SNVG_1
SNVG_2
SNVG_3
SNVG_4
SNVG_5
SNVG_6
SNVG_7
SNVG_8
Bol26
Bol29
NE5248
ZS7
Fr-93-1
NE5267
NE5261
Sh-2-82
NIH8
B31
NIH3
72a
WI91-23
B331
MM1
94a
29805
156a
118a
80a
N40
CA-11-2A
JD1
Z9
217_5
SCW-9
64b
NIH5
0.001

Figure S7. Phylogeny of the linear plasmid lp17

A. A phylogenetic tree of 42 lp17 plasmids from 22 *Borreliella* species.

The tree was constructed and presented as described in the legend to figure S5A. Branches are labeled with species designations. Inconsistencies with the chromosomal tree (figure 4 in text of this report) are discussed in the text of the paper.

B. A phylogenetic tree of lp17 sequences from 29 *B. burgdorferi* isolates.

The tree was constructed and presented as described in the legend to figure S5A, except that the analysis was performed with on the constant region of the plasmid (see text). The first and second columns of the heatmap on the right indicate the geographic origins of the sequenced strains and the within-species SNV groups, respectively.

Figure S7

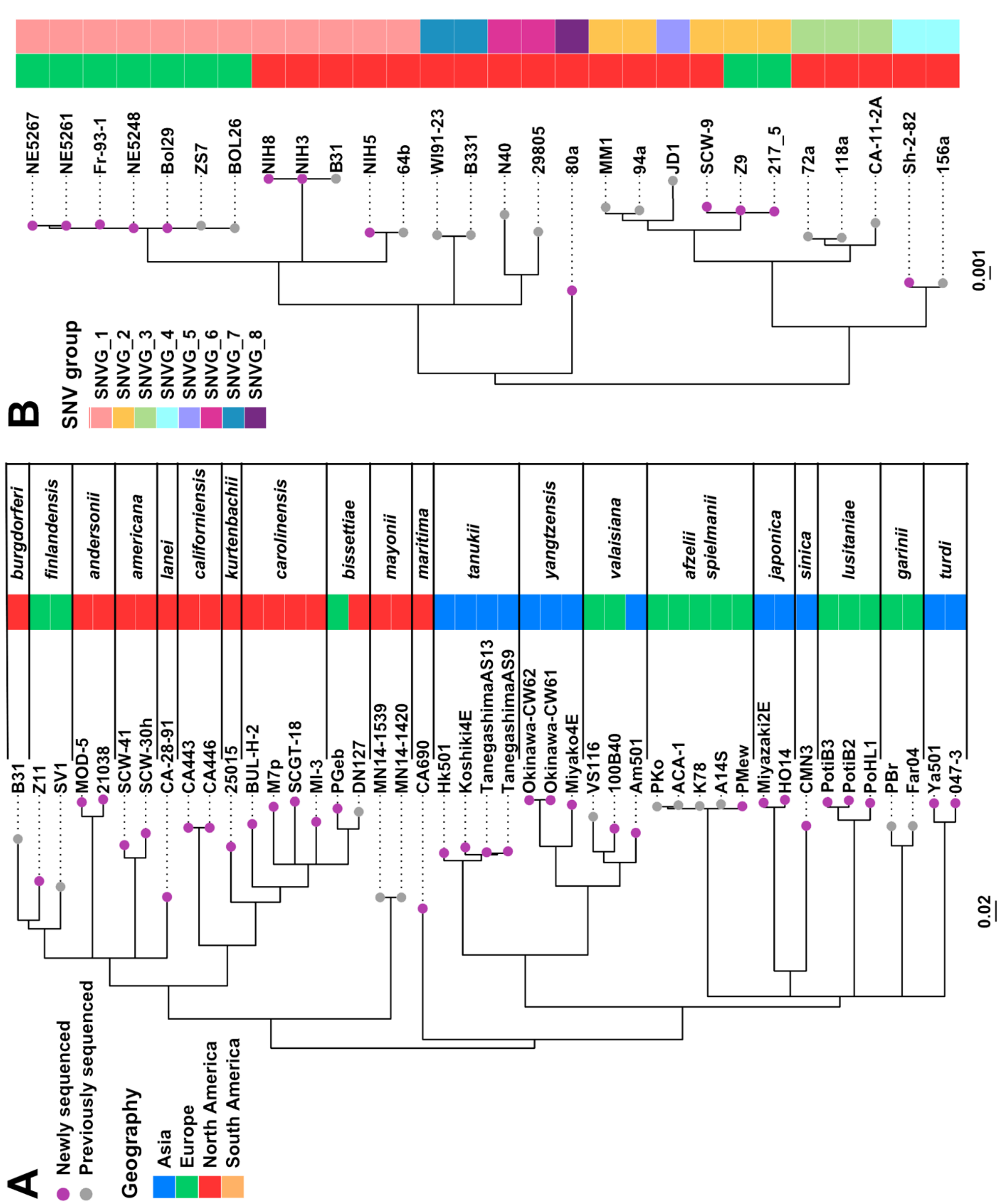
A
Newly sequenced
Previously sequenced
Geography
Asia
Europe
North America
South America
B31
Z11
SV1
MOD-5
21038
SCW-41
SCW-30h
CA-28-91
CA443
CA446
25015
BUL-H-2
M7p
SCGT-18
MI-3
PGeb
DN127
MN14-1539
MN14-1420
CA690
Hk501
Koshiki4E
TanegashimaAS13
TanegashimaAS9
Okinawa-CW62
Okinawa-CW61
Miyako4E
VS116
100B40
Am501
PKo
ACA-1
K78
A14S
PMew
Miyazaki2E
HO14
CMN3
PotiB3
PotiB2
PoHL1
PBr
Far04
Ya501
047-3
0.02
burgdorferi
finlandensis
andersonii
americana
lanei
californiensis
kurtenbachii
carolinensis
bissettiae
mayonii
maritima
tanukii
yangtzensis
valaisiana
afzeli
spielmanii
japonica
sinica
lusitaniae
garinii
turdi
B
SNV group
SNVG_1
SNVG_2
SNVG_3
SNVG_4
SNVG_5
SNVG_6
SNVG_7
SNVG_8
NE5267
NE5261
Fr-93-1
NE5248
Bol29
ZS7
BOL26
NIH8
NIH3
B31
NIH5
64b
WI91-23
B331
N40
29805
80a
MM1
94a
JD1
SCW-9
Z9
217_5
72a
118a
CA-11-2A
Sh-2-82
156a
0.001

Figure S8. Loss of *guaAB* genes from cp26 in *B. sinica* and *B. andersonii*

Genes in the maps are indicated by pointed boxes that show the direction of transcription (green, apparently intact; red, pseudogene). Gene names from *B. burgdorferi* strain B31 are shown on the B31 map. *B. andersonii* 21038 cp26 is very similar to that of MOD-5 shown in the figure, but it has slightly different disruptions in the *guaAB* region.

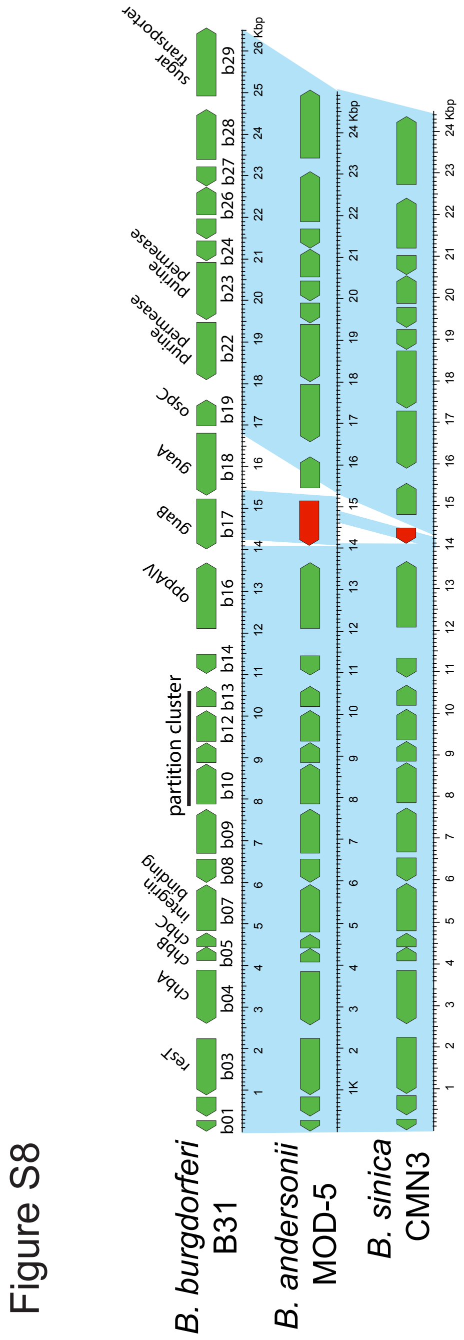

Figure S9. Comparison of chromosome and cp26 trees

Equivalent branches were switched to attain maximum alignment of the chromosome and cp26 SNV trees in Figures 4 and S5, respectively, and green horizontal lines connect the same isolate in the two trees.

The top and bottom major branches show convincing parallel branching, indicating that there has been no transfer of cp26 between these lineages. Since the middle branches (blue lines) were collapsed to the leftmost multi-branch node in the chromosome tree (see legend to Figure 4), it is not possible to conclude that there was no transfer in these isolates, but the alignment shown is consistent with no transfer.

Figure S9

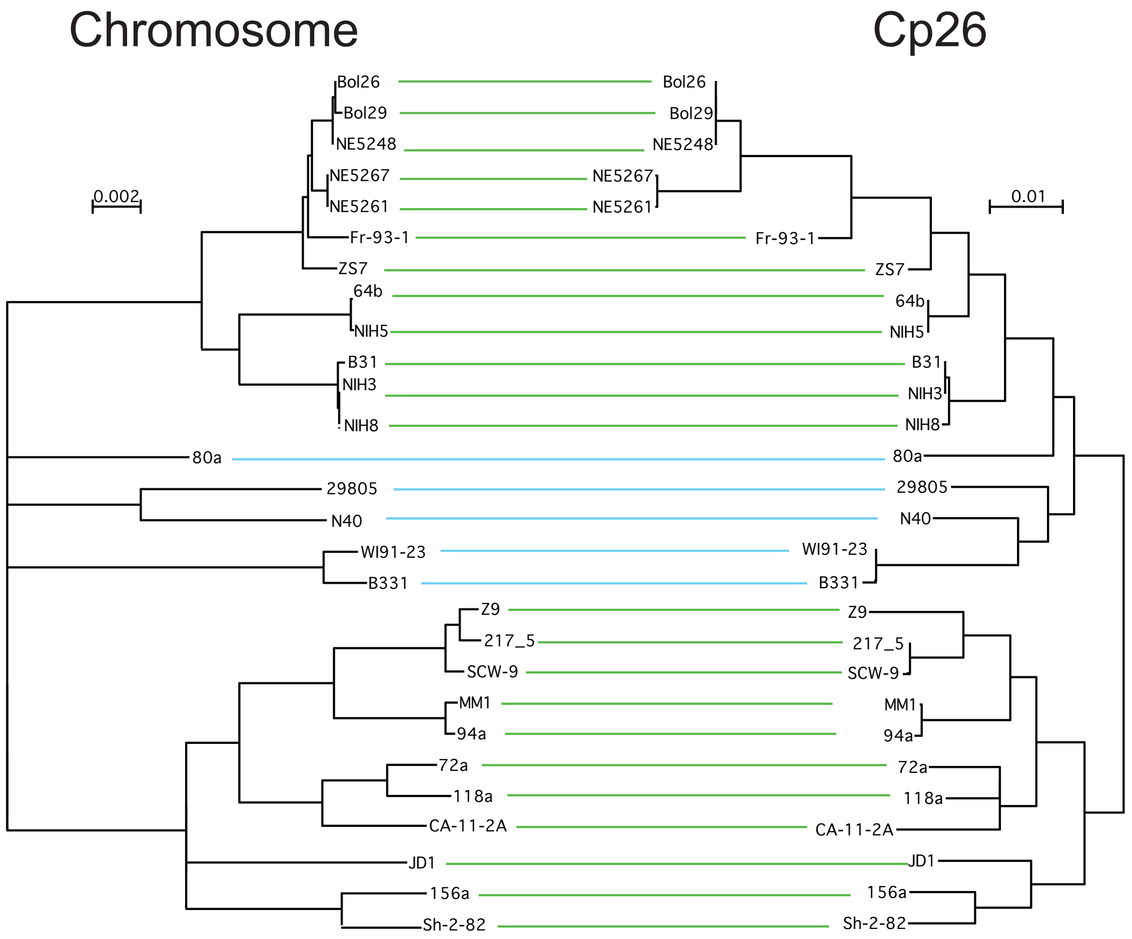

Figure S10. Lp54 terminal differences

Left and right end extensions of newly sequenced lp54 plasmids relative to the lp54 of *B. burgdorferi* are shown with genes indicated by boxes with a pointed end that indicates the direction of transcription. The constant region of the lp54s is shown in black, and homologous terminal extensions have the same color. Genes in the terminal extensions are indicated by blue boxes with their *Borreliella* protein family PFam numbers (see text) if they belong to such a family. Homologs of *B. burgdorferi* strain B31 lp54 "constant region" genes *A01, A03, A74* and *A76*, respectively are shown as orange boxes. lp54s were examined from all the genomes of isolates in Tables S1A and S1B. In species listed without an isolate name, all isolates examined have very similar lp54s. (#) *B. lusitaniae* strain PoHL1 has only three PFam60 genes at its left end. Large asterisks indicate that one or more lp54s of that type have a complete telomere sequence that marks a *bona fide* plasmid end, and small asterisks mark pseudogenes.

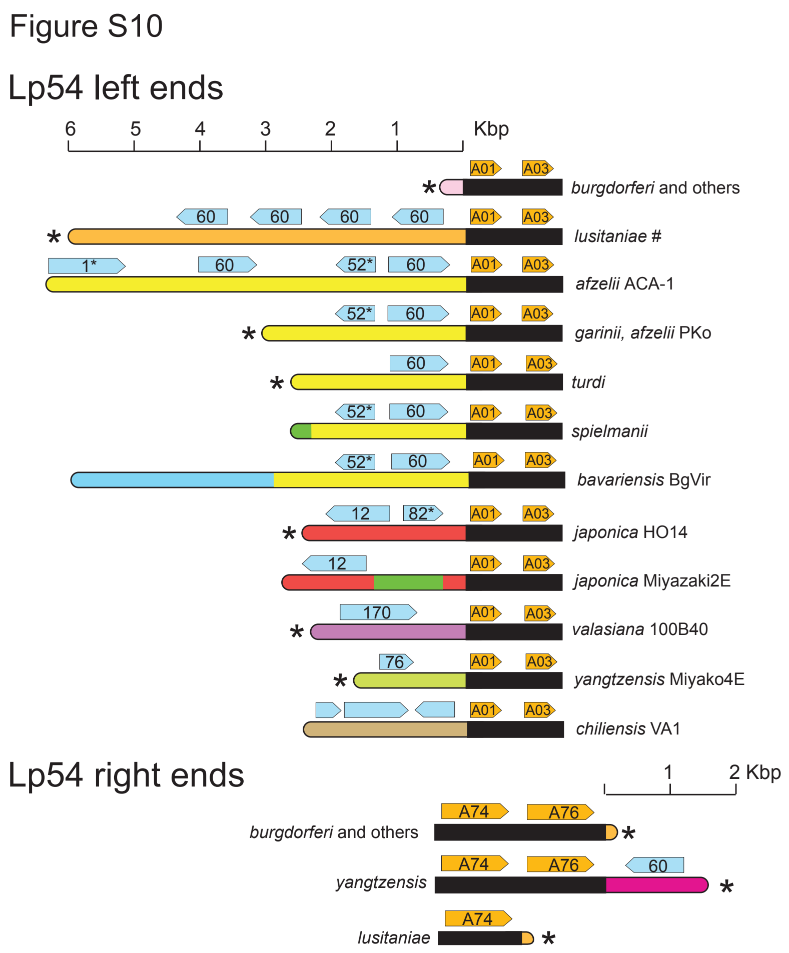

Figure S11. *B. japonica* lp54 structure

Plasmid reading frame maps of lp54 linear plasmids are shown in the format described in figure S3). Strain names are shown at the left, and selected gene names are shown above the B31 map. Green shading between maps indicates homologous regions.

B. japonica HO14 and Miyazaki2E lp54 plasmid sequences contain a left portion (with yellow shading) that is syntenic with B31 lp54. On the right, the map regions with no shading (white) are nearly perfect inverted repeats of the left (yellow) portions. Blue horizontal arrows denote these inverted repeats. The center of the repeat has 78 and 24 bp of unique DNA that are not part of the inverted repeats in HO14 and Miyazaki4E, respectively (vertical red bars). The repeats in HO14 and Miyazaki4E have different center points; it is not known if this is due to independent origins or different deletions that occurred after a single ancestral duplication event.

In the assembled contigs of these sequences the right arms of the repeats are not full length. This is presumably due to the assembly algorithm which cannot separate the two very similar or identical arms of the inverted repeat. Long PacBio sequencing runs across the center of the inverted repeat did not reach further into the right arm. The HO14 lp54 assembly has 11833 bp of inverted repeat right arm sequence, and Miyazaki has 10836 bp. Here the right arm is trimmed to show about seven kbp of the inverted repeat. Most lp54 genes in our HO14 and Miyazaki lp54 assemblies have the same length and are the same length as their orthologs in B31. However there are some differences that appear to be damage in one of the two arms. It is not known if such differences between the two arms are the result of decay of one member of some of the duplicated genes or, perhaps more likely, of sequencing errors that accumulate in one or the other of the two arms during the assembly process.

Marconi *et al.* (1996) previously showed with Southern blot/restriction analysis that HO14 lp54 is in fact a head-to-head inverted repeat dimer, and as discussed above our sequence agrees with this observation. In that previous study we mapped nine restriction enzyme cleavage sites (symmetrically present in both halves of the repeat) made by six different enzymes that agree perfectly with the locations of these sites in the HO14 lp54 sequence. This is strong evidence that the inverted repeat in the HO14 lp54 sequence is not an assembly artifact, and that it accurately reflects the actual plasmid structure. Marconi *et al.* (1996) also showed that isolate IKA2 lp54 was also the size of an expected dimer, suggesting that this peculiar lp54 inverted repeat structure may well be a common feature of all *B. japonica* isolates.

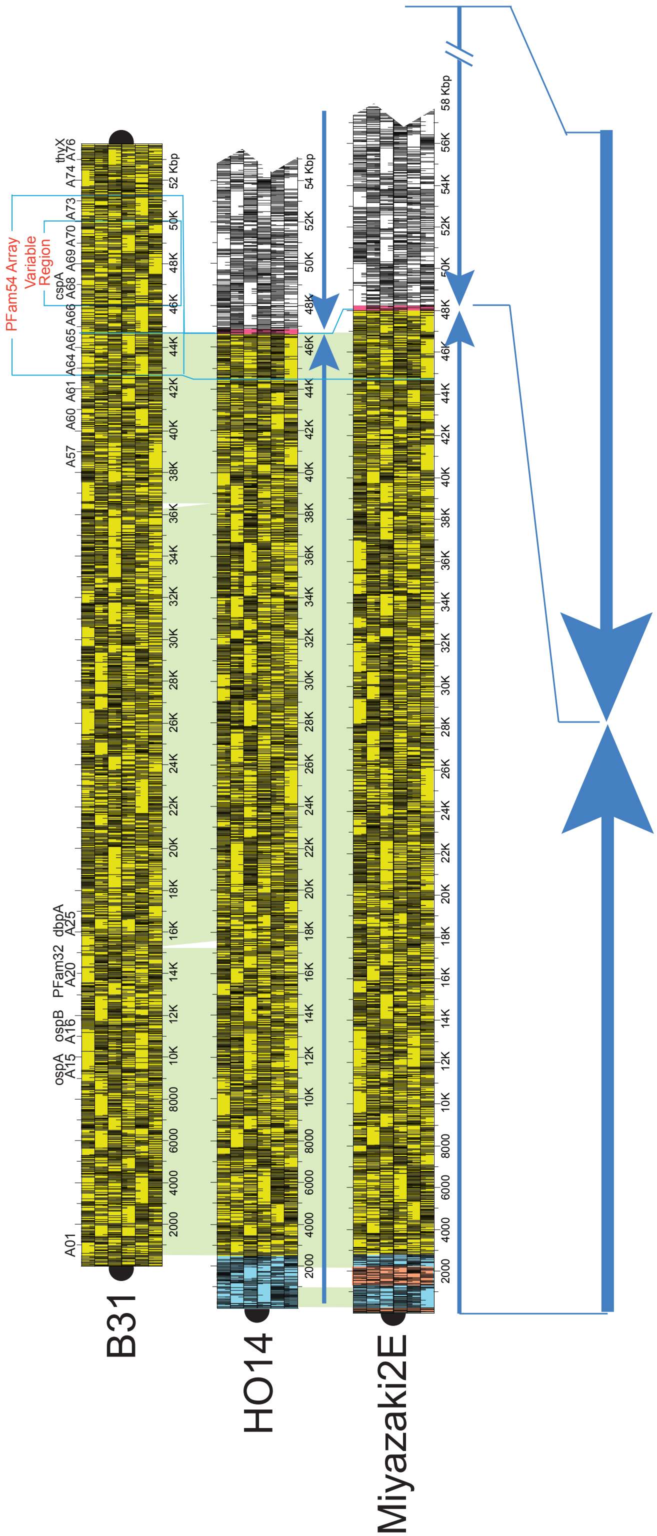
B31
HO14
Miyazaki2E
PFam54 Array
Variable Region
thyX
A74 A76
A73
A69 A70
ospA
A68
A66
A65
A64
A61
A60
A57
dbpA
A25
PFam32
A20
ospB
A16
ospA
A15
A01
52 Kbp
54 Kbp
58 Kbp

Figure S11

Figure S12. Comparison of chromosome and lp54 trees

Equivalent branches were switched to attain alignment of the chromosome and lp54 SNV trees from Figures 4 and S6, respectively. Green horizontal lines connect the same isolate in cases where the branching order in the two trees is consistent. Although positional flexibility of the collapsed branches introduces some uncertainty, six robust and irreconcilable branching order inconsistencies between the two trees are marked by red lines and red stars. Blue numerals in right tree mark the type of PFam54 array carried by the indicated branch as follows: 1, B31like; 2, N40-like; 3, JD1-like; and 4, 80a-like (the sequence of the 217_5 PFam54 array is not complete, but the sequenced portion is very similar to that of Z9 and SCW-9).

Figure S12

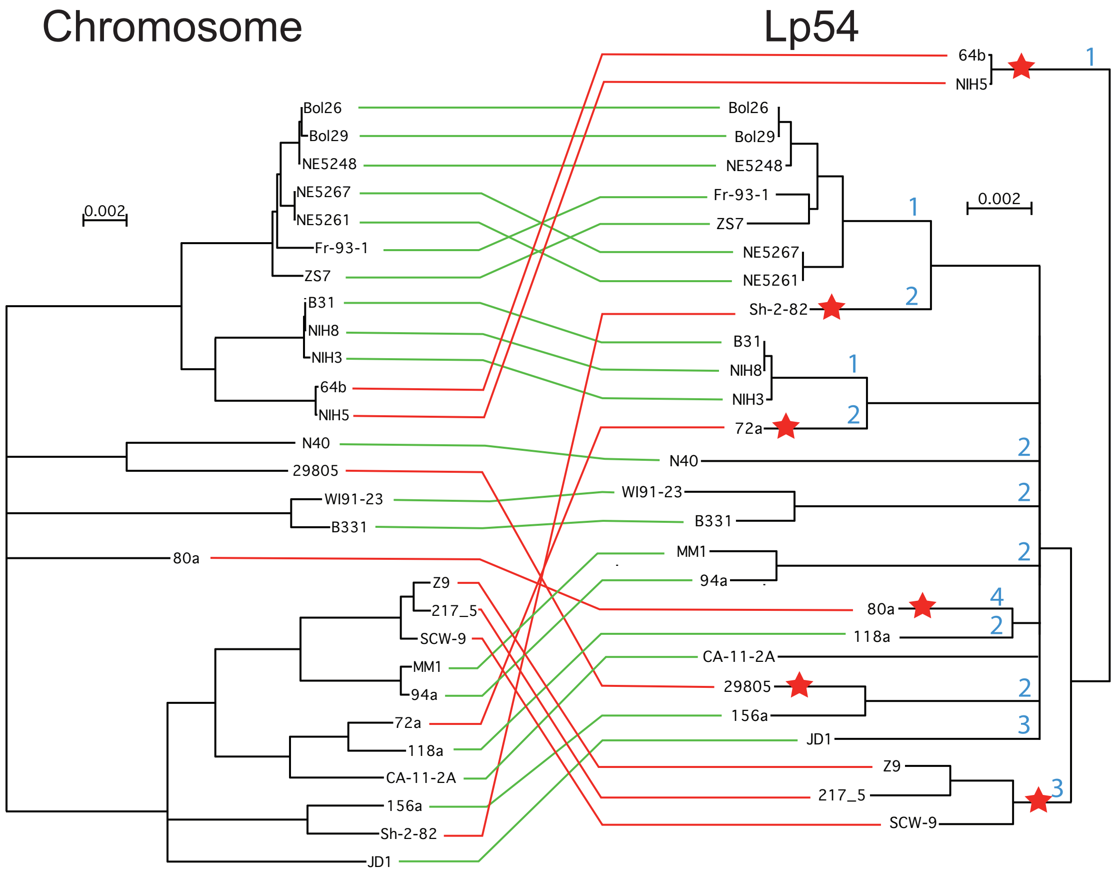

Figure S13. Organizational subtypes of *B. burgdorferi* lp17's

Six-frame ORF maps (formatted as described in figure S3) are shown for the *B. burgdorferi* lp17 plasmids. A typical isolate of each subtype is indicated on the left, and complete membership in each subtype is given in the key below (names in red are those sequences reported here). Selected apparently intact ORFs and pseudogenes are indicated in red and green, respectively. Background colors and green shading between maps indicate similar sequence.

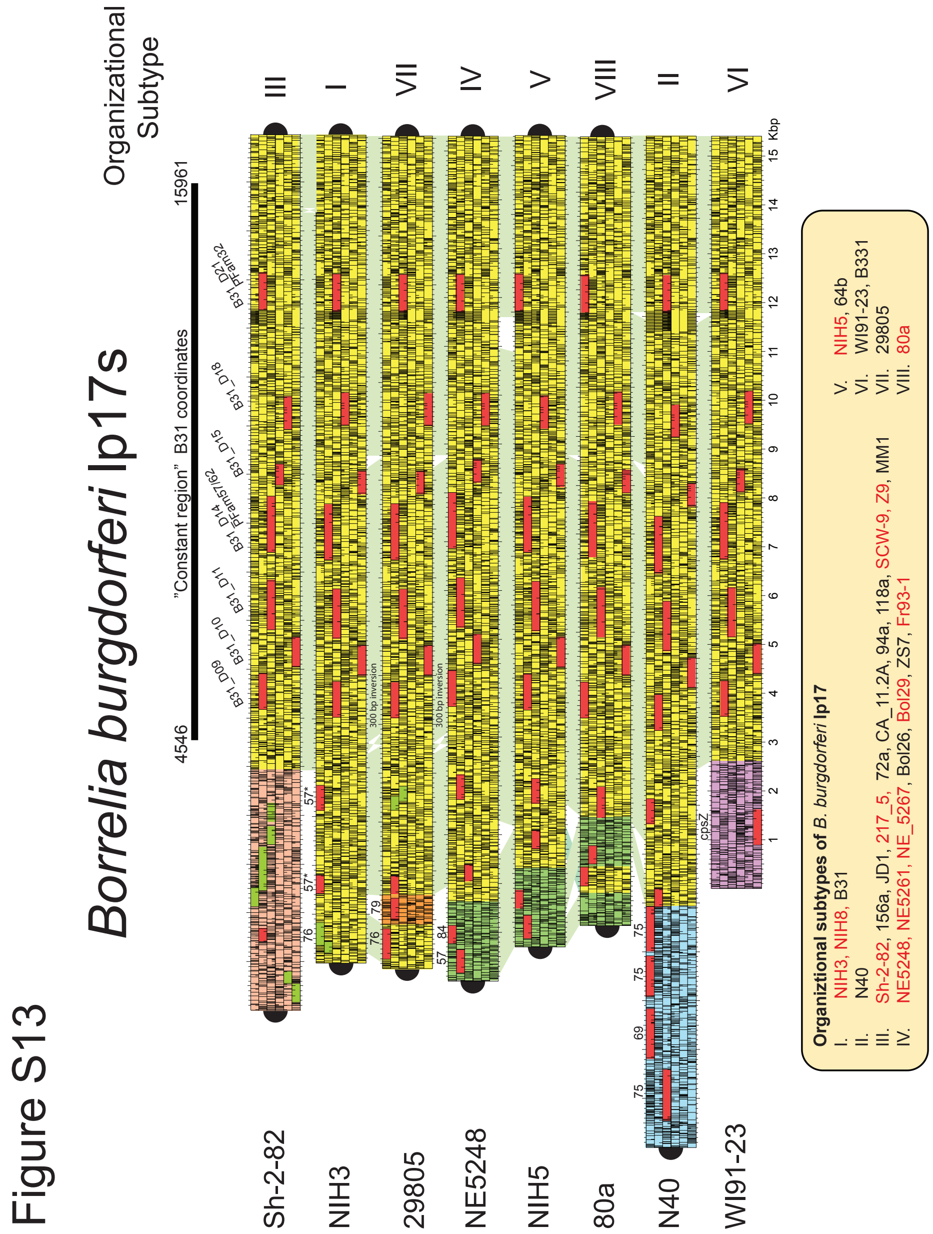

Figure S14. Comparison of chromosome and lp17 trees

Equivalent branches were switched to attain alignment of the chromosome and lp17 SNV trees from Figures 4 and S7, respectively. Green horizontal lines connect the same isolate in cases where the branching order in the two trees is consistent.

Although positional flexibility of the collapsed branches introduces some uncertainty, there are no irreconcilable branching order inconsistencies between the two trees. The red Roman numerals indicate the lp17 organizational subtypes of the various branches.

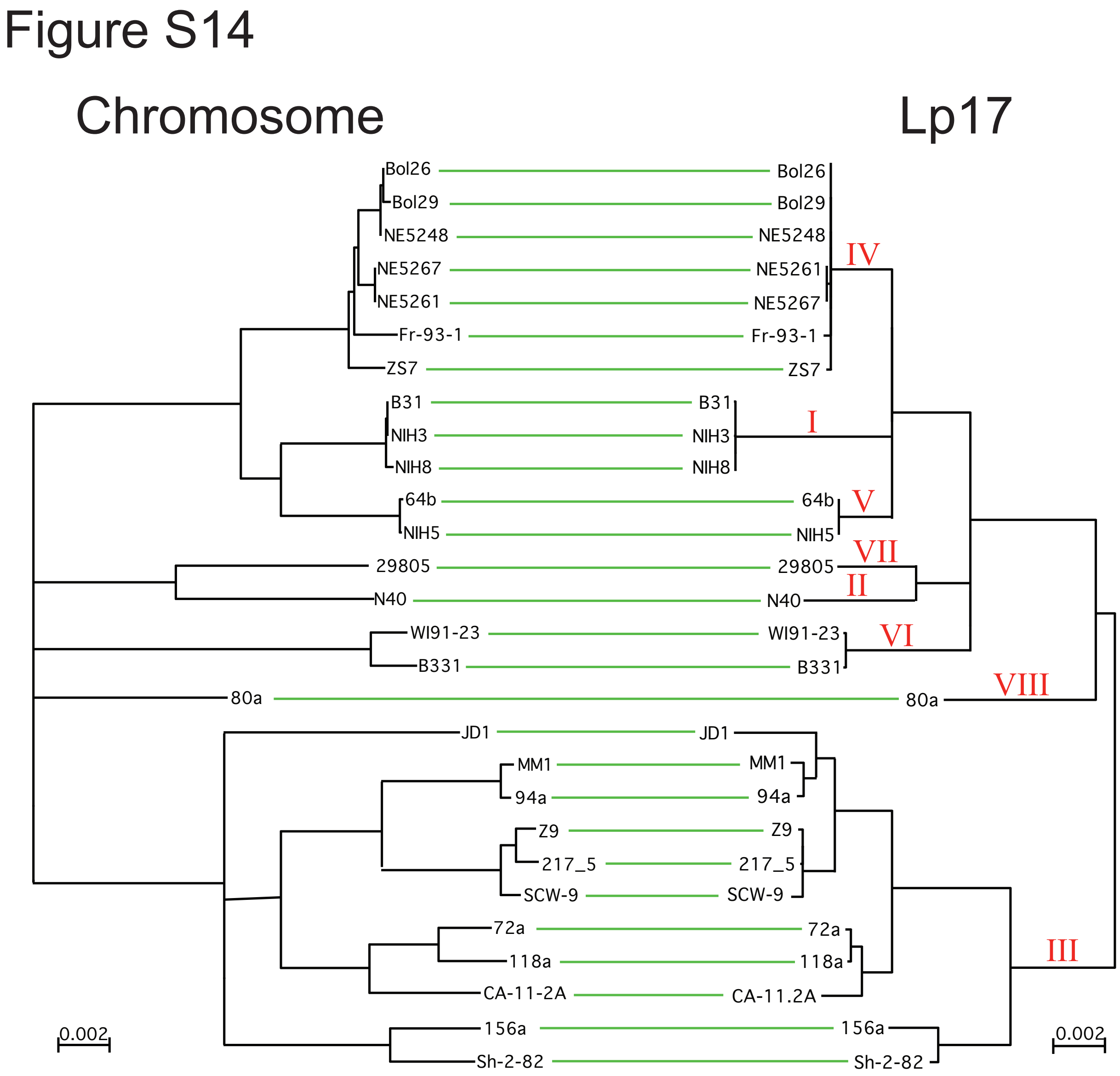

Figure S15. lp17 left end extensions

Six-frame ORF maps (formatted as described in figure S3) are shown for selected lp17 plasmids. Isolate names are on the left, and species are indicated on the right . Background colors and green shading between maps indicate similar sequence. B31 lp17 gene names are given above the constant (yellow) region, and PFam names are given for selected ORFs in the left end extensions. Red asterisks mark the novel sequence joint between the constant region and left end extension (see text).

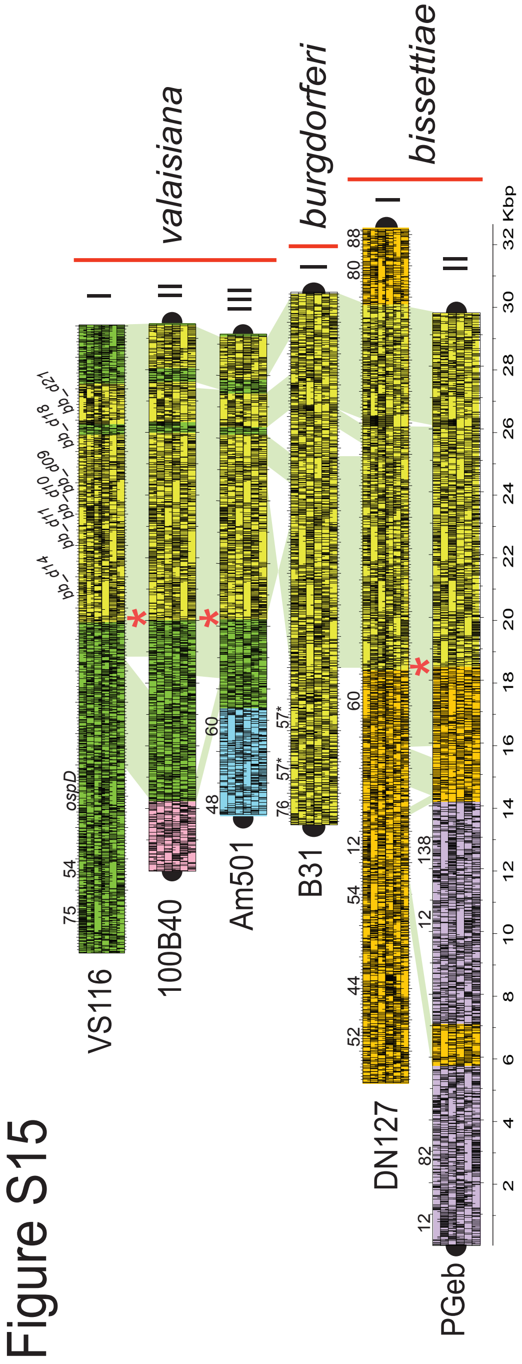

Supplementary Material References

Anderson *et al.* (1988) J. Clin. Microbiol. 26:2209
Anderson *et al.* (1990) J. Clin. Microbiol. 28:2693
Becker *et al.* (2016) BMC Genomics (2016) 17:734
Brenner *et al.* (2012) J. Bacteriol. 194:5713
Casjens *et al.* (2000) Molec. Microbiol. 35:490
Casjens *et al.* (2011a) J. Bacteriol. 193:1489
Casjens *et al.* (2011b) J. Bacteriol. 193:6995
Casjens *et al.* (2012) PLoS One 7:e33280
Casjens *et al.* (2017) BMC Genomics 18:165
Casjens *et al.* (2018) BMC Genomics 19:2018
Collares-Pereira *et al.* (2004) J. Clin. Microbiol. 42:1316
Douglas (1994) Methods Mol. Biol. 25:181
Fedorova *et al.* (2014) Ticks Tick Borne Dis. 5:951
Fingerle *et al.* (2008) Int. J. Med. Microbiol. 298:279
Fraser *et al.* (1997) Nature 309:580
Fukunaga *et al.* (1996) Appl. Env. Microbiol. 62:2338
Glöckner *et al.* (2004) Nucleic Acids Res. 32: 6038
Glöckner *et al.* (2006) BMC Genomics 7:211
Gokuden *et al.* (2006) Annual reports of Kagoshima Prefectural Institute for Environmental Research and Public Health. 6:67 (in Japanese)
Golovchenko *et al.* (2016) Parasites Vectors 9:68
Haven *et al.* (2011) Genetics 189:951
Huang *et al.* (2015) Genome Announc. 3:e01535-14
Jabbari *et al.* (2015) PLoS One 13:e0198135
Kawabata *et al.* (1993) Microbiol. Immunol. 37:843
Kingry *et al.* (2016) PLoS One 11:e0168994
Kurilshikov *et al.* (2014) Genome Announc. 2:e01315-14
Larkin *et al.* (2007) Bioinformatics 23:2947
Marconi *et al.* (1966) J. Bacteriol. 178:3357
Margos *et al.* (2015) Int. J. Syst. Bacteriol. 65:3836
Margos *et al.* (2016) Int. J. Syst. Bacteriol. 66:1447
Margos *et al.* (2017) BMC Genomics 18:422
Margos *et al.* (2018) PLoS One 13:e0208432
Margos *et al.* (2020) Int. J. Syst. Evol. Microbiol. 2020;70:849
Masuzawa *et al.* (2001) Int. J. Med. Microbiol. 51:1817
McVean *et al.* (2002) Genetics 160:1231
Nakao and Miyamoto (1993) Jpn. J. Sanit. Zool. 44:49 (in Japanese)
Nuncio *et al.* (1993) Rev. Port. Doenqas Infec. 16:175
Oliver *et al.* (1995) J. Parasitol. 81:30
Oliver *et al.* (1998) J. Clin. Microbiol. 36:1
Pierer *et al.* (1996) Acta Dermatovenerologica A.P.A. 5:93
Postic *et al.* (2007) Int. J. Med. Microbiol. 297:263
Qiu *et al.* (2004) Proc. Nat'l. Acad. Sci., USA 101:14150
Qiu *et al.* (2008) Emerging Inf. Dis. 14:1097
Rudenko *et al.* (2009a) J. Clin. Microbiol. 47:134
Rudenko *et al.* (2009b) J. Clin. Microbiol. 47:3875

Rudenko *et al.* (2013) Appl. Environ. Microbiol. 79:1444
Rudenko *et al.* (2014) Parasites Vectors 7:4
Schüler *et al.* (2015) PLoS One 10:e0120558.
Schutzer *et al.* (2011) J. Bacteriol. 193:1018
Schutzer *et al.* (2012) J. Bacteriol. 194:545
Schwan *et al.* (1988) Infect. Immun. 56:1831
Schwan *et al.* (1993) J. Clin. Micro. 31:3096
Schwartz *et al.* (2021) Curr. Issues Mol. Biol. 42:409
Travinsky et al. (2010) Emerg. Inf. Dis. 16:1147
